# Reversible *N‐*Heterocyclic Carbene‐Induced α‐H Abstraction in Tungsten(VI) Imido Dialkyl Dialkoxide Complexes

**DOI:** 10.1002/chem.202000840

**Published:** 2020-06-25

**Authors:** Janis V. Musso, Mathis J. Benedikter, Dongren Wang, Wolfgang Frey, Hagen J. Altmann, Michael R. Buchmeiser

**Affiliations:** ^1^ Institute of Polymer Chemistry University of Stuttgart Pfaffenwaldring 55 70569 Stuttgart Germany; ^2^ German Institutes of Textile and Fiber Research (DITF) Denkendorf Körschtalstr. 26 73770 Denkendorf Germany; ^3^ Institute of Organic Chemistry University of Stuttgart Pfaffenwaldring 55 70569 Stuttgart Germany

**Keywords:** alpha-hydrogen elimination, alkylidene, N-heterocyclic carbenes, tungsten

## Abstract

The first reversible *N‐*heterocyclic carbene (NHC) induced α‐H abstraction in tungsten(VI) imido‐dialkyl dialkoxide complexes is reported. Treatment of W(*N*Ar)(CH_2_Ph)_2_(O*t*Bu)_2_ (Ar=2,6‐dichlorophenyl, 2,6‐dimethylphenyl, 2,6‐diisopropylphenyl) with different NHCs leads to the formation of complexes of the type W(*N*Ar)(CHPh)(NHC)(CH_2_Ph)(O*t*Bu) in excellent isolated yields of up to 96 %. The highly unusual release of the *tert‐*butoxide ligand as *t*BuOH in the course of the reaction was observed. The formed alkylidene complexes and *t*BuOH are in an equilibrium with the NHC and the dialkyl complexes. Reaction kinetics were monitored by ^1^H NMR spectroscopy. A correlation between the steric and electronic properties of the NHC and the reaction rates was observed. Kinetics of a deuterium‐labeled complex in comparison to its non‐deuterated counterpart revealed the presence of a strong primary kinetic isotope effect (KIE) of 4.2, indicating that α‐H abstraction is the rate‐determining step (RDS) of the reaction.

## Graphical abstract

**Time to say good bye**: The *N*‐heterocyclic carbene‐induced α‐H elimination in tungsten(VI) imido dialkyl dialkoxide complexes offers access to tungsten(VI) imido alkylidene monoalkyl monoalkoxide complexes in excellent yields of up to 96 %.

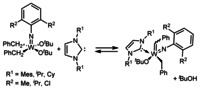

Since the characterization of the first tungsten‐carbene complexes by Fischer in 1964^1^ the number of group 6 carbene complexes has steeply increased and yielded a variety of highly applicable developments,^2^, ^3^ one of the most prominent being the discovery of olefin metathesis‐active homogeneous Group 6 catalysts by Schrock.^4^ The key step in the synthesis of those catalysts is the formation of an alkylidene complex which is—among alternative routes^5^, ^6^, ^7^—usually achieved by the intramolecular abstraction of an α‐hydrogen atom of an alkyl ligand and elimination of the hydrogen‐accepting ligand.^8^, ^9^ This process is believed to be induced by a sterically crowded coordination sphere around the metal atom.^10^, ^11^ Nomura and Zhang reported the successful use of NHCs for the α‐H abstraction of vanadium(V) complexes of the type V(*N*R)(CH_2_SiMe_3_)_3_ to the corresponding alkylidene complexes V(*N*R)(CHSiMe_3_)(NHC)(CH_2_SiMe_3_) [R=1‐adamantyl, 2,6‐Me_2_C_6_H_5_, NHC=1,3‐bis(2,6‐diisopropylphenyl)imidazole‐2‐ylidene] (Scheme 1).^12^ Those results encouraged us to initiate studies towards the NHC‐induced α‐H abstraction in tungsten(VI) imido dialkyl complexes in order to find a more direct route to highly metathesis‐active neutral and cationic tungsten imido alkylidene NHC complexes similar to those that have previously been synthesized by our group.^13^ To our delight, the reaction of W(*N*‐2,6‐Cl_2_C_6_H_5_)(CH_2_Ph)_2_(O*t*Bu)_2_ (**1**) with 1,3‐dimesitylimidazol‐2‐ylidene (IMes) led to the rapid formation of the alkylidene complex W(*N*‐2,6‐Cl_2_C_6_H_5_)(CHPh)(IMes)(CH_2_Ph)(O*t*Bu) (**2**) in benzene at room temperature, which was obtained as a 1:1 mixture of the corresponding *syn*‐ and *anti‐*isomer (^1^ *J*_CH_(*syn*)=118.9 Hz, ^1^ *J*_CH_(*anti*)=140.1 Hz, Scheme 2).

**Scheme 1:**
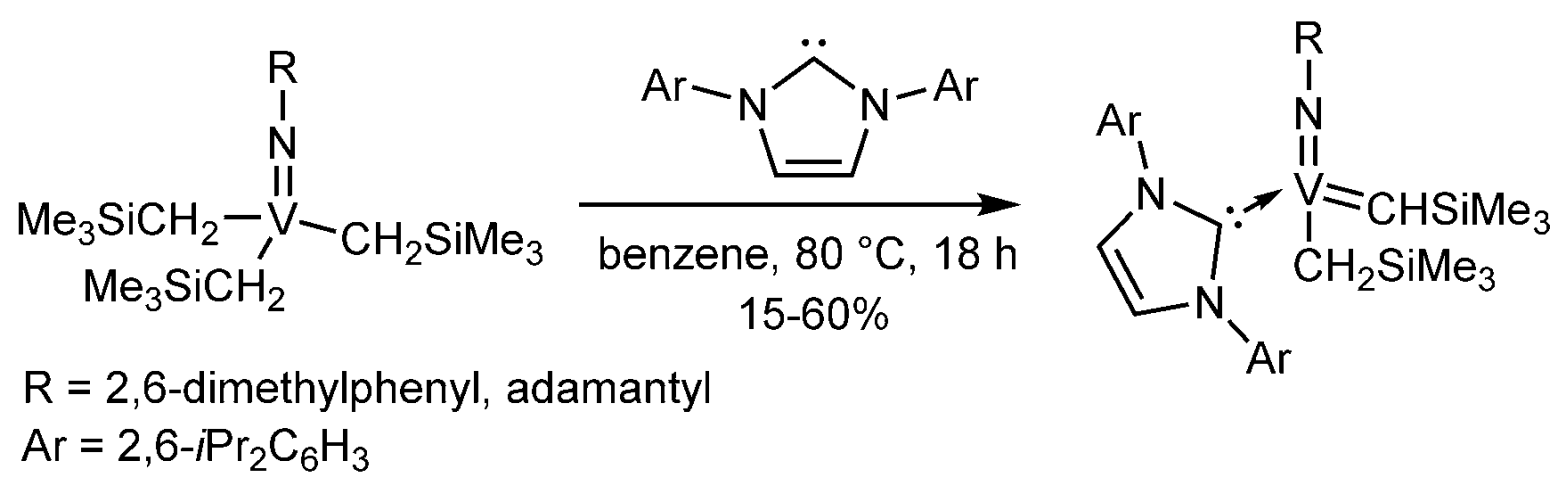
Nomura′s NHC‐induced α‐H abstraction in vanadium(V) complexes.

**Scheme 2:**
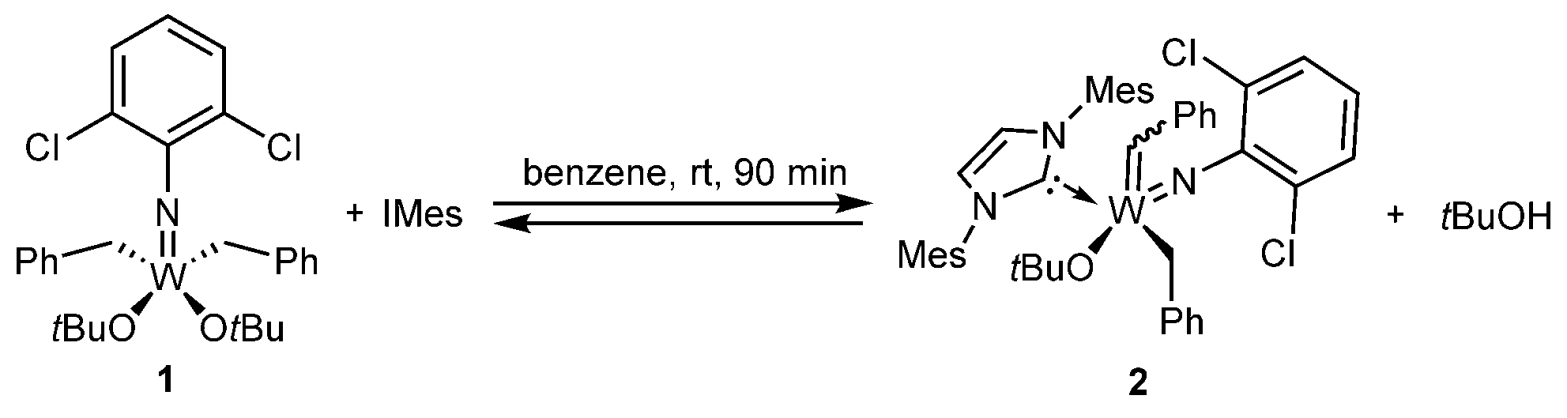
Reaction of **1** with IMes to **2** under concomitant elimination of *t*BuOH.

At a first glance, the involvement of one of the *t*BuO ligands in the α‐H abstraction instead of the alkyl group and its elimination as *t*BuOH appears surprising. However, the rather shielded α‐carbon in the dialkyl species (*δ*=66.1 ppm) point towards a low alkylidene character of the M−C bond and a full involvement of the p*‐*orbital in the M−C, C−H and C−C bonds. In turn, this leads to a lower basicity compared to the fully developed lone pair of the alkoxide.^14^ Notably, for a quantitative description, the values for *δ*_11_, *δ*_22_ and *δ*_33_ would have to be determined. Although the elimination of alkoxides in silica‐supported metathesis catalysts has been proposed as a possible deactivation pathway in computational studies,^15^, ^16^ to the best of our knowledge, this is the first case in organometallic chemistry in which the elimination of an alkoxide ligand is exploited synthetically while the benzyl ligand does not act as an acceptor for the eliminated hydrogen as observed in α‐H abstraction reactions of other tungsten(VI) imido dialkyl dialkoxide complexes.^9^

The reaction of **1** with IMes was conducted again in C_6_D_6_ and monitored by ^1^H NMR spectroscopy. At an initial concentration of 37 mm of IMes and **1**, the reaction reached its equilibrium state at 40 % conversion after 90 min. We hypothesize that the eliminated *t*BuOH is evaporated to a large extent in course of the work‐up procedure, thereby shifting the equilibrium towards the product side, which serves as an explanation for the isolated yield of 78 %. To prove this assumption, *t*BuOH was added to the isolated alkylidene complex **2**, which indeed resulted again in the described equilibrium.

Single crystals of **2** were grown from a pentane solution at −35 °C. A thermal ellipsoid drawing of the structure is depicted in Figure 1. Compound **2** crystallizes in the monoclinic space group, *P*2_1_/*n*, *a*=1031.21(3) pm, *b*=2104.93(8) pm, *c*=1896.91(5) pm, *α*=*γ*=90°, *β*=91.105(2)°, *Z*=4. In the solid state, **2** adopts a square pyramidal (SP) configuration with the alkylidene ligand occupying the apical position and all other ligands laying in the equatorial plane. The sterical bulk of the mesityl groups leads to a high degree of distortion of the SP configuration, which is indicated by a calculated *τ*_5_ value^17^ of 0.34. The NHC takes the position *trans* to the benzyl ligand, which shows a rather long bond distance for a W−C single bond of 231.2(6) pm compared to other tungsten alkyl complexes.^9^ This can be explained by a structural *trans* effect induced by the strong σ‐donor properties of the NHC.

**Figure 1:**
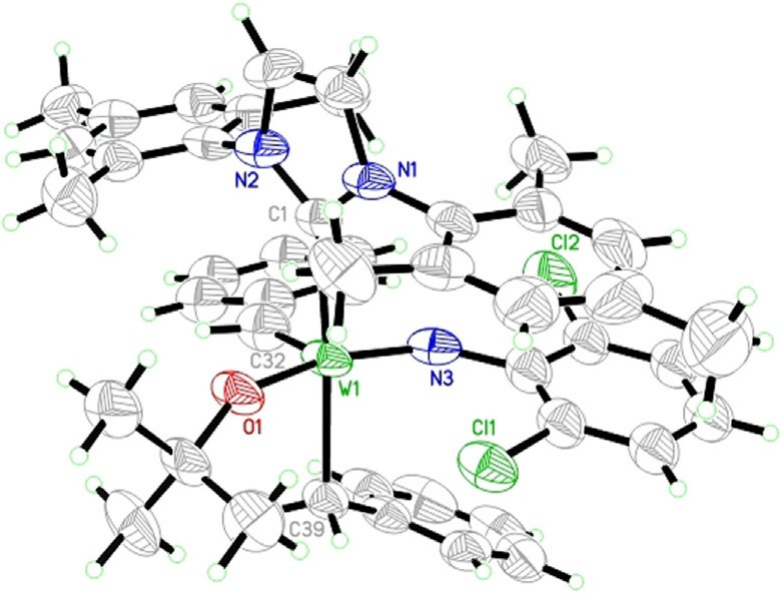
Single crystal X‐ray structure of **2** displayed as a thermal ellipsoid plot (50 % probability). Relevant bond lengths (pm) and angles (°): W(1)−N(3) 1.704(4), W(1)−O(1) 1.920(4), W(1)−C(32) 1.911(5), W(1)−C(39) 2.250(7), W(1)−C(1) 2.312(6), C(1)−N(1) 1.373(9), C(1)−N(2) 1.371(9); N(3)‐W(1)‐O(1) 148.9(3), N(3)‐W(1)‐C(32) 104.9(4), O(1)‐W(1)‐C(32) 106.1(3), N(3)‐W(1)‐C(39) 91.7(2), O(1)‐W(1)‐C(39) 87.3(2), C(32)‐W(1)‐C(39) 90.0(4), N(3)‐W(1)‐C(1) 94.3(2), O(1)‐W(1)‐C(1) 83.1(2), C(32)‐W(1)‐C(1) 96.8(3), C(39)‐W(1)‐C(1) 169.5(2), N(1)‐C(1)‐N(2) 102.5(5).

A screening of different NHCs and tungsten complexes with a variation of the imido ligands is depicted in Scheme 3. With the exception of **9**, all complexes were obtained in good to excellent isolated yields between 69 % (**10**) and 96 % (**7**). Reactions were conducted in benzene at room temperature and gave access to the desired products in short reaction times of—in most cases—only several minutes to a few hours. We assume that a 2,6‐substitiution pattern of the imido‐ligand contributes to a crowded coordination sphere and is therefore required for an efficient α‐H abstraction, although we have no experimental evidence, since the preparation of the analogous W(*N*‐3,5‐dichlorophenyl)(CH_2_Ph)_2_(O*t*Bu)_2_ and W(*N*‐3,5‐dimethylphenyl)(CH_2_Ph)_2_(O*t*Bu)_2_ complexes failed. While the reaction of the analogous W^VI^ imido dineophyl dialkoxide complexes with various NHCs also resulted in the desired α‐H abstraction, the products turned out to be unstable at room temperature and readily decomposed before full characterization could be completed.

**Scheme 3:**
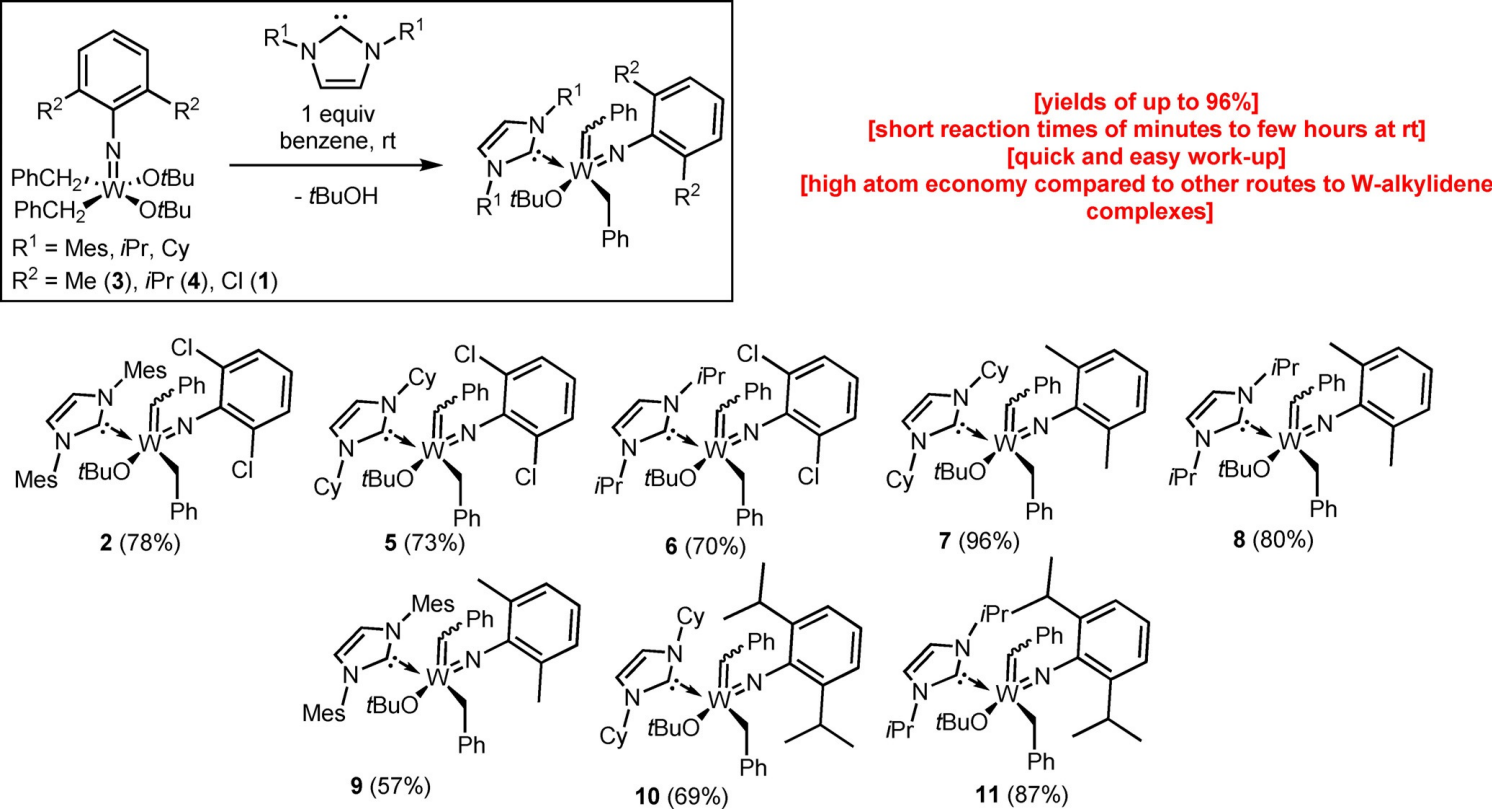
Scope of NHC‐induced α‐H abstraction.

It was observed that both the reaction rates and yields highly depend on the combination of the imido ligand the NHC used. Thus, the reaction of IMes and **6** required several days to reach equilibrium and resulted only in a moderate yield of 58 % whereas no conversion was observed for the analogous reaction with the more sterically demanding 1,3‐diisopropylimidazol‐2‐ylidene (IPr).

Similar to **2**, in the solid state, **11** adopts a SP configuration and crystallizes in the monoclinic space group, *P*2_1_/*c*, *a*=2053.71(9) pm, *b*=961.56(4) pm, *c*=2024.87(9) pm, *α*=*γ*=90°, *β*=112.431(2)°, *Z*=4 (Figure 2). The τ_5_ value was 0.016, indicating an almost perfect SP configuration. It can be assumed that the lower degree of distortion originates from the lower steric demand of the isopropyl groups of the NHC compared to **2**. The lower steric bulk of IPr is reflected by higher reaction rates compared to IMes for R^2^=Me (Table 1).

**Figure 2:**
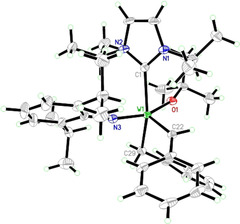
Single crystal X‐ray structure of **11** displayed as a thermal ellipsoid plot (50 % probability). Relevant bond lengths (pm) and angles (°): W(1)−N(3) 1.7756(19), W(1)−O(1) 1.9049(16), W(1)−C(22) 1.912(2), W(1)−C(29) 2.225(2), W(1)−C(1) 2.279(2), C(1)−N(1) 1.359(3), C(1)−N(2), N(3)‐W(1)‐O(1) 154.72(8), N(3)‐W(1)‐C(22) 101.39(10), O(1)‐W(1)‐C(22) 103.70(9), N(3)‐W(1)‐C(29) 94.02(9), O(1)‐W(1)‐C(29) 86.79(8), C(22)‐W(1)‐C(29) 95.31(10), N(3)‐W(1)‐C(1) 87.05(8), O(1)‐W(1)‐C(1) 82.20(8), C(22)‐W(1)‐C(1) 108.30(9), C(29)‐W(1)‐C(1) 155.70(9), N(1)‐C(1)‐N(2) 103.7(2).

**Table 1:** Reaction kinetics of NHC‐induced α‐H abstraction.

*k*_25 °C_ (L mol^−1^ s^−1^)	R^1^=Cy	R^1^=^*i*Pr^	R^1^=Mes
R^2^=Cl	2.72±0.06×10^−1^	9.24±0.19×10^−2^	6.31±1.3×10^−3^
R^2^=Me	1.12±0.01×10^−2^	4.93± 0.09×10^−3^	4.93±0.15×10^−4^
R^2^=*i*Pr	7.51±0.07×10^−4^	4.93±0.15×10^−4^	n.d.^[a]^

[a] No conversion.

It was anticipated that the reaction kinetics correlate with the Tolman electronic parameter (TEP)^18^, ^19^ and the percent buried volume (%*V*_bur_)^20^, ^21^ of the NHC. The kinetics of several reactions were therefore monitored by ^1^H NMR and compared to the steric and electronic properties of the NHCs used as summarized in the literature (Table 2).^18^, ^21^ The combination of the comparably small and electron‐withdrawing 2,6‐dichlorophenylimido ligand with 1,3‐dicyclohexylimidazol‐2‐ylidene (ICy) and IPr, both having a %*V*_bur_ of 27.5 and a TEP of 2049.5 cm^−1^ and 2050.3 cm^−1^, respectively, resulted in fast reaction rates with *k*_25 °C_=2.27×10^−1^ L mol^−1^ s^−1^ and *k*_25 °C_=9.24×10^−1^ L mol^−1^ s^−1^ that were pushing the limits of reaction monitoring by ^1^H NMR. The rate constant for the reaction of the same complex with the more sterically demanding IMes (36.5 %*V*_bur_) was determined as *k*_25 °C_=6.31×10^−3^ L mol^−1^ s^−1^. This trend continues in the reactions of **3**. With ICy, the highest reaction rate of *k*_25 °C_=1.12×10^−2^ L mol^−1^ s^−1^ was observed, while the reaction rates with IPr and IMes were determined as *k*_25 °C_=4.93×10^−3^ L mol^−1^ s^−1^and *k*_25 °C_=4.93×10^−4^ L mol^−1^ s^−1^. Lastly, the reactions of **4** with IPr and ICy, respectively, revealed smaller rate constants compared to the analogous reactions of **3**, which can be explained by the higher steric bulk of the isopropyl groups. This can also be considered the reason for the poor reactivity of **4** and IMes, for which no conversion was observed. No intermediates were observed by NMR In all the monitored reactions, however, their formation could not be fully ruled out at that stage. Therefore, two reaction pathways seemed plausible (Scheme 4).

**Table 2:** Buried volumes (%*V*_bur_) and Tolman electronic parameters (TEP) of the NHCs used.^18^, ^21^

	%*V*_Bur_ (@ 2 Å, AuCl(NHC))	TEP (cm^−1^)
R^1^=*i*Pr	27.5	2050.3
R^1^=Cy	27.5	2049.5
R^1^=Mes	36.5	2049.6

**Scheme 4:**
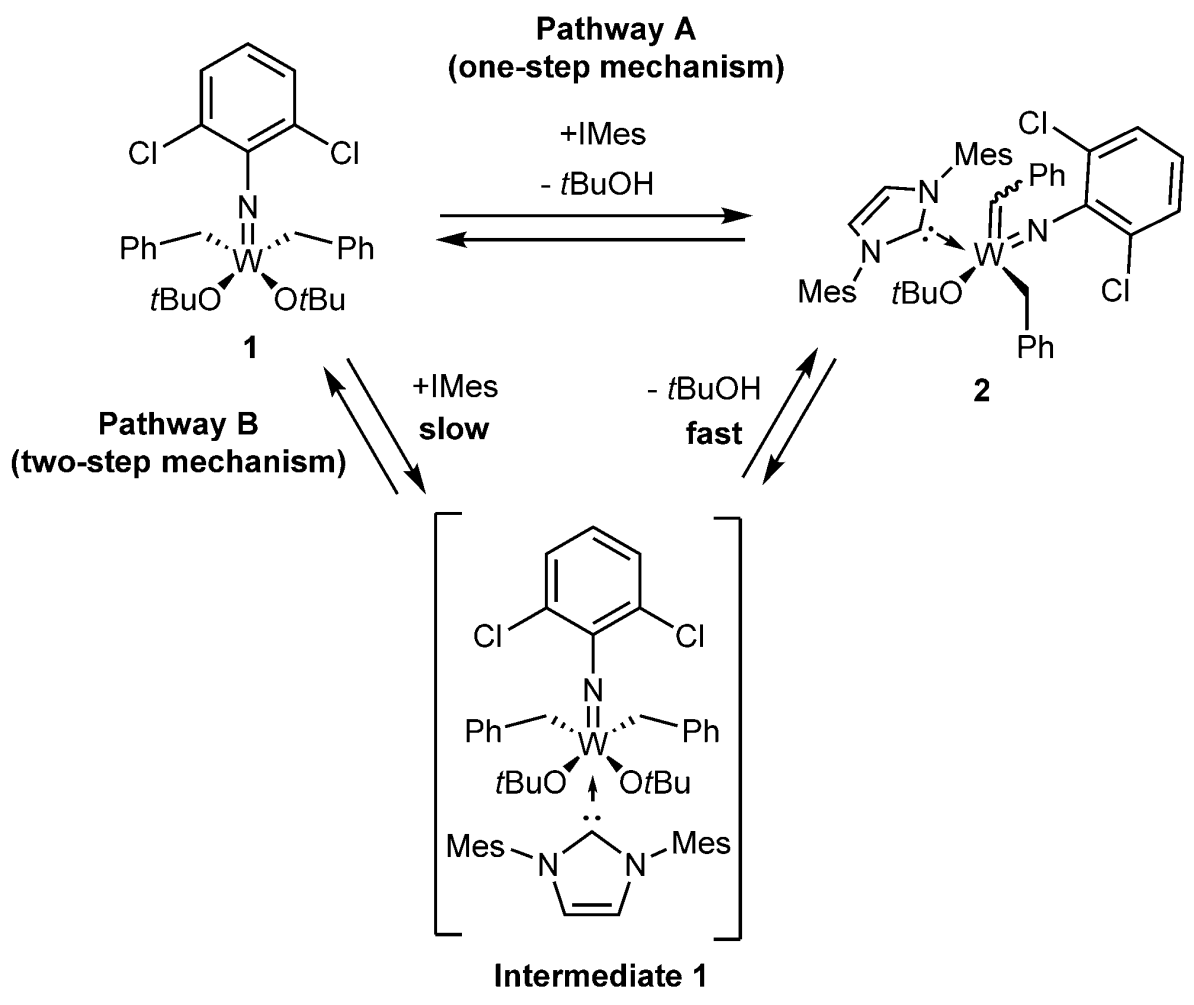
Plausible reaction pathways for α‐H abstraction.

One feasible pathway proceeds via a *concerted* mechanism (**Pathway A**). The NHC binds to the metal and at the same time *t*BuOH is eliminated without the formation of intermediates. In this case, C−H bond cleavage is involved in the rate‐determining step (RDS). On the contrary, a two‐step mechanism is plausible, in which the binding of the NHC results in the formation of **Intermediate 1** which, in a consecutive step, undergoes α‐H abstraction (**Pathway B**). The first step has to be slow and therefore the RDS since the buildup of **Intermediate 1** was not observed by NMR. As shown by *Schrock* et al.; deuterium‐labeling can be an efficient tool for the elucidation of the mechanistic details of the formation of tungsten alkylidene complexes.^22^ Therefore, the deuterium‐labeled complex **1 d** was synthesized from commercially available toluene‐d_8_ in a radical chlorination reaction using sulfuryl chloride,^23^ subsequent synthesis of benzylmagnesium chloride‐d_7_,^24^ which in the reaction with W(*N*‐2,6‐dichlorophenyl)Cl_2_(O*t*Bu)_2_(THF) gave access to **1 d**. The kinetics of the reaction of **1 d** and IMes were monitored with the aforementioned technique to obtain a rate constant of *k*_25 °C_=1.49(±0.02)×10^−3^ L mol^−1^ s^−1^ (Scheme 5). A comparison of the kinetics of the non‐deuterated complex **1** and the deuterated complex **1 d** revealed a kinetic isotope effect (KIE) of k_H_/k_D_=4.2. This can be considered a strong primary KIE^25^ and is an indication that C−H bond cleavage is involved in the RDS.^26^ Consequently, coordination of the NHC in pathway B cannot be rate determining. Together with the mechanistic considerations and the observed absence of intermediates in ^1^H NMR, we conclude that a concerted mechanism (Pathway A) is operative.

**Scheme 5:**
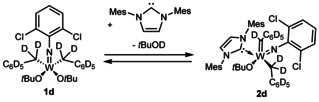
Reaction of deuterated complex **1 d** with IMes.

Based on the data outlined above, another reaction mechanism that comprises the deprotonation of the dialkyl complex by the NHC (Scheme 6) is also highly unlikely since signals of intermediary imidazolium salts could not be observed by ^1^H NMR spectroscopy.

**Scheme 6:**
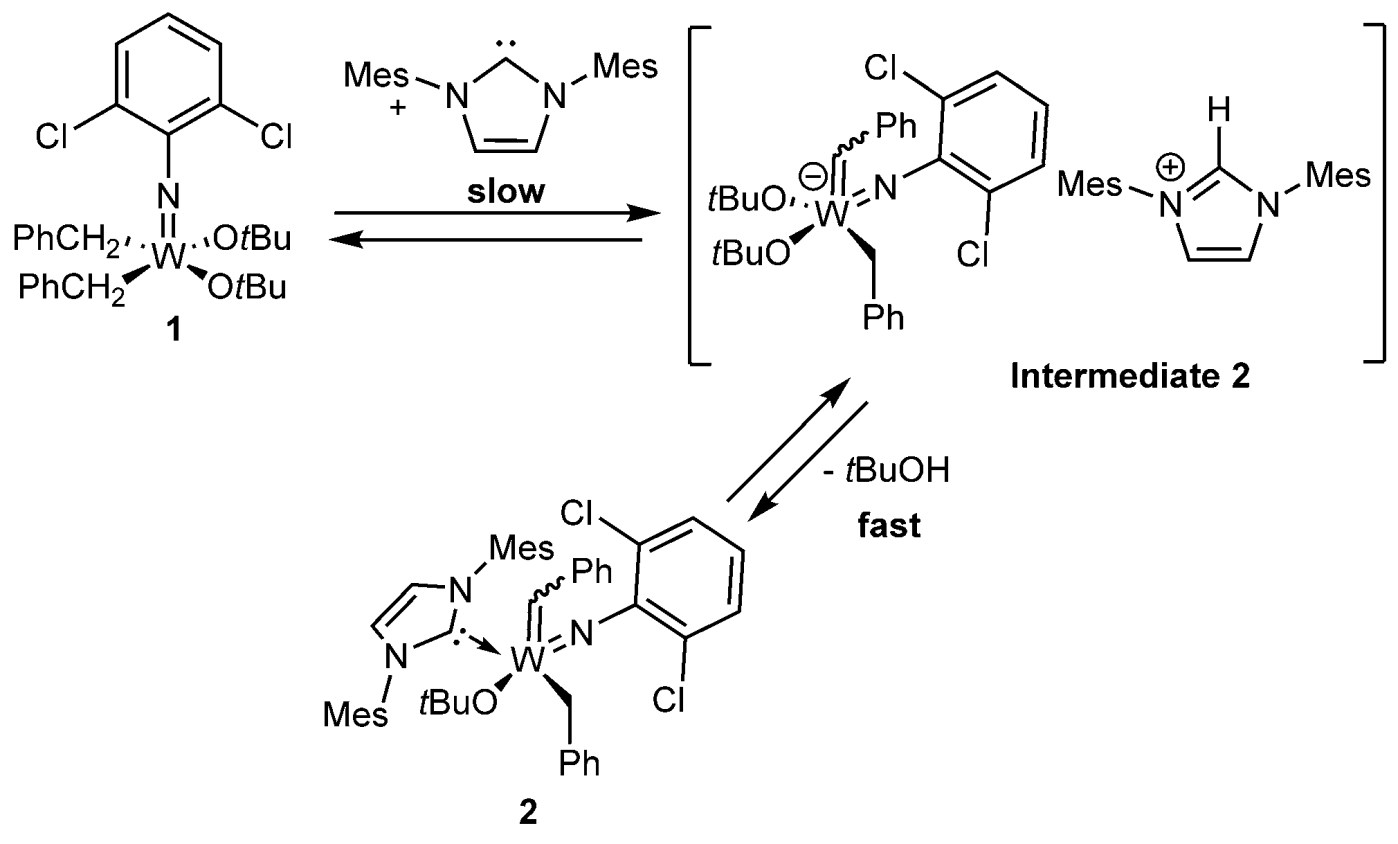
Reaction mechanism involving an imidazolium salt.

In this hypothetical case, the deprotonation step of the dialkyl complex **1** to **intermediate 2** has to be slow and therefore rate‐determining. The subsequent protonation of an O*t*Bu‐ligand, on the other hand, would have to be fast since **intermediate 2** was not observed by ^1^H NMR. There are several indications, that this mechanism is not operative. However, Nomura′s investigations on the NHC induced α‐H abstraction of vanadium(V) trialkyl complexes show that, in some cases, the extremely basic NHCs^27^, ^28^ with p*K*_b_ values in the range of −6.8 to −11.2^18^ are interchangeable with only mildly basic phosphines such as PMe_3_ 29 having a p*K*_b_ of 5.35 (p*K*_b_ values calculated from the p*K*_a_ values of the conjugated acids in aqueous solution).^30^, ^31^ In addition, our kinetic measurements suggest that the rate constant is in correlation with the σ‐donor properties of the used NHCs whereas no link to basicity was found. The combination of those considerations again leads us to conclude that a concerted mechanism is present.

In summary, the first reversible NHC induced α‐H abstraction in tungsten(VI) imido dialkyl complexes has been accomplished in excellent isolated yields of up to 96 %. The reaction sequence can be considered highly atom‐economic compared to previously published routes to W‐alkylidene complexes^6^, ^9^, ^32^, ^33^ since the only byproducts in the 4‐step sequence from W(O)Cl_4_ are CO_2_, LiCl, MgCl_2_ and *t*BuOH. We found that the reaction kinetics correlate with the electronic and steric properties, both of the imido ligand and the NHC, and the α‐H abstraction is the RDS of the reaction. The formed alkylidene complexes are excellent candidates for the synthesis of highly metathesis active cationic complexes similar to those that have been published previously by our group^13^, ^34^, ^35^, ^36^ since they already contain the NHC that is required for the delocalization of the positive charge.^37^ We are currently working on those cationic complexes; results will be reported in due course.

## Experimental details and characterization data

The Supporting Information is available free of charge from the publisher.

CCDC  1981737 (**2**), 1981738 (**5**) and 1981739 (**11**) contain the supplementary crystallographic data for this paper. These data are provided free of charge by The Cambridge Crystallographic Data Centre.

## Conflict of interest

The authors declare no conflict of interest.

## Supporting information

As a service to our authors and readers, this journal provides supporting information supplied by the authors. Such materials are peer reviewed and may be re‐organized for online delivery, but are not copy‐edited or typeset. Technical support issues arising from supporting information (other than missing files) should be addressed to the authors.

Supplementary
