# Supplementary material for: Reversible N‐Heterocyclic Carbene‐Induced α‐H Abstraction in Tungsten(VI) Imido Dialkyl Dialkoxide Complexes
Source: Chemistry. 2020 Jun 25;26(40):8709–13. doi: 10.1002/chem.202000840 (PMC7496809; doi:10.1002/chem.202000840)
Supplement: Supplementary file 1 — Supplementary [file CHEM-26-8709-s001.pdf]

Chemistry–A European Journal

Supporting Information

Reversible *N*-Heterocyclic Carbene-Induced α-H Abstraction in Tungsten(VI) Imido Dialkyl Dialkoxide Complexes

Janis V. Musso,[a] Mathis J. Benedikter,[a] Dongren Wang,[a] Wolfgang Frey,[c]
Hagen J. Altmann,[a] and Michael R. Buchmeiser*[a, b]

Table of contents

Table of contents	Page
General	2
Synthesis of Compounds	2
Reversibility of α-H Abstraction	10
General Procedures for Kinetic Measurements	10
Kinetic Measurements	12
NMR Spectra	26
X-Ray Data	40
References	76

General: All reactions were conducted under the exclusion of air by standard Schlenk techniques. Reactions involving the synthesis of metal complexes were performed in an N_2 filled glove box (MBraun Labmaster 130). Glassware was either stored at 120 °C for 2 h and cooled in an evacuated antechamber or dried at 550 °C under high vacuum (0.05 mbar).

$W(O)Cl_4$[1], W(*N*-2,6-diisopropylphenyl)$Cl_2(O^tBu)_2$(THF)[2] and W(*N*-2,6-dimethylphenyl)$Cl_2(O^tBu)_2$(THF)[3] were synthesized according to literature procedures.

NMR: ^{1}H-NMR and ^{13}C-NMR spectra were recorded using a Bruker Avance III 400 (400 MHz for ^{1}H, 101 MHz for ^{13}C) spectrometer. Chemical shifts are reported in ppm relative to tetramethylsilane with the solvent resonance resulting from residual solvent protons (C_6D_6 7.16 ppm, CD_2Cl_2 5.32 ppm, DMSO-d6 2.50 ppm) as reference. Data are reported as follows: chemical shift, multiplicity (s = singlet, d = doublet, t = triplet, q = quartet, quint = quintet, sept = septet, br = broad, m = multiplet), integration and coupling constants (Hz). ^{13}C Spectra were recorded on a Bruker Avance III 400 (100 MHz for ^{13}C) spectrometer. Chemical shifts of ^{13}C-NMR spectra of are reported in ppm downfield from tetramethylsilane with the solvent resonance (C_6D_6 128.4 ppm, CD_2Cl_2 54.0 ppm, DMSO-d6 39.5 ppm) as reference.

Elemental analyses: Elemental analyses were carried out by the Institute of Inorganic Chemistry, University of Stuttgart, Germany.

Solvents: Toluene, CH_2Cl_2, THF, diethyl ether and pentane were dried by using an MBraun SPS-800 solvent purification system with alumina drying columns and stored over 4 Å Linde type molecular sieves. Deuterated solvents were filtered over activated alumina and stored over 4 Å Linde type molecular sieves inside a dry box.

Benzyl Chloride-d7

Benzyl chloride-d7 was synthesized according to a modified literature procedure[4] starting from commercially available deuterated toluene. Analytical data were in accordance with the literature.[5-6]

Toluene-d7 (21.7 g, 216 mmol, 2.00 equiv) and sulfuryl chloride (14.6 g, 108 mmol, 1.00 equiv) were placed in Schlenk flask equipped with a reflux condenser. Dibenzoylperoxide (100 mg, 310 µmol, 0.003 equiv) was added and the reaction mixture was heated to reflux for 25 min. The crude mixture was purified by fractionated distillation to furnish the product as colorless liquid (7.75g, 54%).

Benzylmagnesium Chloride-d7

Benzylmagnesium chloride-d7 was synthesized according to a modified literature procedure[7] and the concentration of the solution was determined by titration with salicylaldehyde phenylhydrazone.[8]

A solution of benzyl chlorde-d7 (5.42 g, 40.6 mmol, 1.00 equiv) in THF (22 mL) containing a small amount of 1,2-dibromoethane (400 uL) as initiator was added dropwise to a stirred suspension of magnesium turnings (1.30 g, 53.5 mmol, 1.10 equiv) in THF (22 mL). The mixture was heated to reflux for 1 h by which most of the magnesium had dissolved and the solution turned grayish yellow. The solution was filtered over celite and used without further purification. The product was obtained as a gray solution of benzylmagnesium chloride-d7 in THF (44 mL, 0.62 M, 67%).

W(*N*-2,6-dichlorophenyl)Cl$_4$(MeCN)

2,6-Dichlorophenyl isocyanate (6.05 g, 32.2 mmol, 1.10 equiv) and W(O)Cl$_4$ (10.0 g, 29.3 mmol, 1.00 equiv) were suspended in toluene (250 mL) in a Schlenk flask. After refluxing for 18 h, the toluene was removed *in vacuo*. The resulting green solid was washed with MeCN and dried *in vacuo*. Additional product was recrystallized from the supernatant solution. The product was obtained as a dark green solid (12.3 g, 80%). **^{1}H** NMR (400 MHz, DMSO-d6) δ 7.45 (d, J = 8.1 Hz, 2H, $C_6Cl_2\underline{H_3}$), 6.87 (t, J = 8.1 Hz, 1H, $C_6Cl_2\underline{H_3}$), 2.08 (s, 3H, $\underline{Me}$CN); **^{13}C** NMR (101 MHz, DMSO-d6) δ 147.7 ($\underline{C_6}Cl_2H_3$), 132.7 ($\underline{C_6}Cl_2H_3$), 127.7 ($\underline{C_6}Cl_2H_3$), 126.3 ($\underline{C_6}Cl_2H_3$), 118.1 (Me$\underline{C}$N), 64.9 ($\underline{Me}$CN). Anal. Calcd for $C_8H_6Cl_6N_2W$: C, 18.24; H, 1.15; N, 5.32. Found: C, 18.22; H, 1.22; N, 5.39.

W(*N*-2,6-dichlorophenyl)Cl$_2$(O^{t}Bu)$_2$(THF)

^{t}BuOLi (6.97 g, 87.0 mmol, 2.00 equiv) was dissolved in THF (100 mL) and added slowly over 30 min to a solution of W(*N*-2,6-dichlorophenyl)Cl$_4$(MeCN) (24.3 g, 21.6 mmol, 1.00 equiv) in THF (250 mL) at -78 °C (solution turned from green to golden, green precipitate starts to dissolve). After warming to rt, the solution was stirred for 4 h before removing the solvent *in vacuo*. The product was dissolved in diethyl ether (250 mL) and filtered over celite. Crystallization from diethyl ether was conducted by partial evaporation of the solvent *in vacuo* to obtain the product as a canary yellow solid (20.7 g, 75%). **^{1}H** NMR (400 MHz, DMSO-d6) δ 7.51 (d, J = 8.1 Hz, 2H, $C_6Cl_2\underline{H_3}$), 6.92 (t, J = 8.1 Hz, 1H, $C_6Cl_2\underline{H_3}$), 3.67 – 3.52 (m, 4H, THF), 1.82 – 1.67 (m, 4H, THF), 1.36 (s, 18H, C$\underline{Me_3}$); **^{13}C** NMR (101 MHz, DMSO) δ 145.9 ($\underline{C_6}Cl_2H_3$), 133.6 ($\underline{C_6}Cl_2H_3$), 128.2 ($\underline{C_6}Cl_2H_3$), 127.5 ($\underline{C_6}Cl_2H_3$), 85.5 (THF), 67.0 ($\underline{C}Me_3$), 29.9 (C$\underline{Me_3}$), 25.1(THF); Anal. Calcd for $C_{18}H_{29}Cl_4NO_3W$: C, 34.15; H, 4.62; N, 2.21. Found: C, 33.85; H, 4.44; N, 2.26.

W(*N*-2,6-dichlorophenyl)(CH$_2$Ph)$_2$(O^{t}Bu)$_2$

A solution of benzylmagnesium chloride in THF (3.5 mL, 2.0 M, 2.20 equiv) was added dropwise to a solution of W(*N*-2,6-dichlorophenyl)(O^{t}Bu)$_2$Cl$_2$ (2.00 g, 3.16 mmol, 1.00 equiv) in diethyl ether (30 mL) at rt. The reaction mixture was stirred for 1 h and the solvent was removed *in vacuo*. The product was dissolved in a small amount of pentane (50 mL) and filtered over celite. Recrystallization from pentane furnished the product as orange crystals (1.82 g, 86%). **^{1}H** NMR (400 MHz, C_6D_6) δ 7.29 – 7.24 (m, 4H, CH$_2\underline{Ph}$), 7.23 – 7.18 (m, 4H, CH$_2\underline{Ph}$), 7.04 (d, *J* = 8.1 Hz, 2H, $C_6Cl_2\underline{H_3}$), 6.93 (t, *J* = 7.2 Hz, 2H, CH$_2\underline{Ph}$), 6.11 (t, *J* = 8.1 Hz, 1H, $C_6Cl_2\underline{H_3}$), 3.27 (s, 4H, $\underline{CH_2}$Ph), 1.22 (s,

18H, $C\underline{Me_3}$); **^{13}C** NMR (101 MHz, C_6D_6) δ 149.9 ($\underline{C_6}Cl_2H_3$), 143.1 ($CH_2\underline{Ph}$), 142.9 ($CH_2\underline{Ph}$), 133.0 ($\underline{C_6}Cl_2H_3$), 132.6 ($CH_2\underline{Ph}$), 127.8 ($CH_2\underline{Ph}$), 125.6 ($\underline{C_6}Cl_2H_3$), 125.4 ($\underline{C_6}Cl_2H_3$), 84.9 ($\underline{C}Me_3$), 66.1 ($\underline{C}H_2Ph$), 31.4 ($C\underline{Me_3}$); Anal. Calcd for $C_{28}H_{35}Cl_2NO_2W$: C, 50.02; H, 5.25; N, 2.08. Found: C, 49.97; H, 5.247; N, 2.05.

W(*N*-2,6-dichlorolphenyl)($CD_2C_6D_5$)$_2$(O^{t}Bu)$_2$

A solution of benzylmagnesium chloride-d7 in THF (5.6 mL, 0.62 M, 2.20 equiv) was cooled to -35 °C and added dropwise to a chilled solution (-35 °C) of W(*N*-2,6-dichlorophenyl)(OtBu)$_2$Cl$_2$ (1.00 g, 1.58 mmol, 1.00 equiv) in diethyl ether (30 mL). After reaching rt, the reaction mixture was stirred for 1 h and the solvent was removed *in vacuo*. The product was dissolved in pentane (50 mL) and filtered over celite. Recrystallization from pentane furnished the product as orange crystals (720 mg, 66%). **^{1}H** NMR (400 MHz, CD_2Cl_2) δ 7.43 (d, *J* = 8.1 Hz, 2H, $C_6Cl_2\underline{H_3}$), 6.81 (t, *J* = 8.1 Hz, 1H, $C_6Cl_2\underline{H_3}$), 1.28 (s, 18H, $C\underline{Me_3}$). **^{13}C** NMR (101 MHz, CD_2Cl_2) δ 149.5 ($\underline{C_6}Cl_2H_3$), 142.7 ($CD_2\underline{C_6}D_5$), 132.4 ($\underline{C_6}Cl_2H_3$), 132.3 ($CD_2\underline{C_6}D_5$), 132.1 ($CD_2\underline{C_6}D_5$), 131.8 ($CD_2\underline{C_6}D_5$), 127.7 ($\underline{C_6}Cl_2H_3$), 127.5 ($CD_2\underline{C_6}D_5$), 127.3 ($CD_2\underline{C_6}D_5$), 125.4 ($\underline{C_6}Cl_2H_3$), 124.8 ($CD_2\underline{C_6}D_5$), 124.5 ($CD_2\underline{C_6}D_5$), 124.3 ($CD_2\underline{C_6}D_5$), 84.8 ($\underline{C}Me_3$), 64.9 ($\underline{C}D_2Ph$), 64.7 ($\underline{C}D_2Ph$), 64.5 ($\underline{C}D_2Ph$), 64.3 ($\underline{C}D_2Ph$), 64.1 ($\underline{C}D_2Ph$), 31.3 ($C\underline{Me_3}$); Anal. Calcd for $C_{48}H_{21}D_{14}Cl_2NO_2W$: C, 48.99; N, 2.04. Found: C, 48.58; N, 2.00 (H cannot be determined due to mixture of H/D).

W(*N*-2,6-dimethylphenyl)(CH_2Ph)$_2$(O^{t}Bu)$_2$

A solution of benzylmagnesium chloride in THF (6.0 mL mL, 2.0 M, 2.20 equiv) was cooled to -35 °C and added dropwise to a chilled solution (-35 °C) of W(*N*-2,6-dimethylphenyl)(OtBu)$_2$Cl$_2$ (3.21 g, 5.42 mmol, 1.00 equiv) in diethyl ether (50 mL). After reaching rt, the reaction mixture was stirred for 1 h and the solvent was removed *in vacuo*. The product was dissolved in pentane (100 mL) and filtered over celite. Recrystallization from pentane furnished the product as a red microcrystalline soild (2.18 g, 64%). **^{1}H** NMR (400 MHz, CD_2Cl_2) δ 7.41 – 7.25 (m, 6H, $CH_2\underline{Ph}$), 7.21 – 7.15 (m, 4H, $CH_2\underline{Ph}$, $C_6Me_2\underline{H_3}$), 7.06 – 6.94 (m, 3H, $CH_2\underline{Ph}$, $C_6Me_2\underline{H_3}$), 2.92 (s, 4H, $\underline{CH_2}Ph$), 2.76 (s, 6H, $C_6\underline{Me_2}H_3$), 1.48 (s, 18H, $C\underline{Me_3}$); **^{13}C** NMR (101 MHz, CD_2Cl_2) δ 153.0 ($\underline{C_6}Me_2H_3$), 144.8 ($CH_2\underline{Ph}$), 134.7 ($CH_2\underline{Ph}$), 131.5 ($CH_2\underline{Ph}$), 127.9 ($CH_2\underline{Ph}$), 127.1 ($\underline{C_6}Me_2H_3$), 126.1 ($\underline{C_6}Me_2H_3$), 124.3 ($\underline{C_6}Me_2H_3$), 84.3 ($\underline{C}Me_3$), 63.9 ($\underline{C}H_2Ph$), 31.6 ($C\underline{Me_3}$), 19.5 ($Ar\underline{Me_2}$); Anal. Calcd for $C_{30}H_{41}NO_2W$: C, 57.06; H, 6.54; N, 2.22. Found: C, 56.86; H, 6.693; N, 2.05.

W(*N*-2,6-diisopropylphenyl)(CH_2Ph)$_2$(O^{t}Bu)$_2$

A solution of benzylmagnesium chloride in THF (11.4 mL mL, 2.00 M, 2.20 equiv) was cooled (-35 °C) and added dropwise to a chilled solution (-35 °C) of W(*N*-2,6-diisopropylphenyl)(OtBu)$_2$Cl$_2$ (6.72 g, 10.4 mmol, 1.00 equiv) in diethyl ether (50 mL). After reaching rt, the reaction mixture was stirred for 1.5 h and the solvent was removed *in vacuo*. The product was dissolved in pentane (100 mL) and filtered over celite. Recrystallization from pentane furnished the product as a bright orange

microcrystalline soild (6.11 g, 86%). **^{1}H** NMR (400 MHz, CD_2Cl_2) δ 7.22 – 7.16 (m, 2H, $CH_2\underline{Ph}$), 7.16 – 7.08 (m, 4H, $CH_2\underline{Ph}$), 7.03 – 6.93 (m, 5H, $CH_2\underline{Ph,}$ $C_6{}^{i}Pr_2\underline{H_3}$), 6.88 – 6.78 (m, 2H, $C_6{}^{i}Pr_2\underline{H_3}$), 3.85 (hept, *J* = 6.9 Hz, 2H, $C_6\underline{{}^{i}Pr_2}H_3$), 2.74 (s, 4H, $\underline{CH_2}Ph$), 1.30 (s, 18H, $C\underline{Me_3}$), 1.27 (d, *J* = 6.9 Hz, 12H, $C_6\underline{{}^{i}Pr_2}H_3$); **^{13}C** NMR (101 MHz, CD_2Cl_2) δ 150.4 ($\underline{C_6}{}^{i}Pr_2H_3$), 145.2 ($CH_2\underline{Ph}$), 144.4 ($CH_2\underline{Ph}$), 131.3 ($CH_2\underline{Ph}$), 127.9 ($CH_2\underline{Ph}$), 126.7 ($\underline{C_6}{}^{i}Pr_2H_3$), 124.2 ($\underline{C_6}{}^{i}Pr_2H_3$), 122.6 ($\underline{C_6}{}^{i}Pr_2H_3$), 85.2 ($\underline{C}Me_3$), 63.2 ($\underline{C}H_2Ph$), 31.7 ($C\underline{Me_3}$), 28.4 ($Ar\underline{C}HMe_2$), 24.4 ($ArCH\underline{Me_2}$); Anal. Calcd for $C_{34}H_{49}NO_2W$: C, 59.39; H, F7.18; N, 2.04. Found: C, 59.02; H, 7.110; N, 2.07.

W(*N*-2,6-dichlorophenyl)(CHPh)(1,3-dimesitylimidazol-2-ylidene)(CH_2Ph)(O^tBu)

1,3-Dimesitylimidazol-2-ylidene (304 mg, 997 µmol, 1.00 equiv) and W(*N*-2,6-dichlorophenyl)$(CH_2Ph)_2(O^tBu)_2$ (670 mg, 997 µmol, 1.00 equiv) were weighed in a vial and dissolved in a small amount of benzene (1 mL). The reaction mixture was stirred at room temperature for 90 min. Then the solvent was evaporated *in vacuo* and co-evaporated with pentane (3 x 2 mL). Subsequently, the product was washed with a small amount of pentane (3 x 2 mL) to furnish the product as a yellow powder (702 mg, 78%). Single crystals for X-ray analysis were grown from a pentane solution to yield bright yellow crystals. **^{1}H** NMR (**both isomers**) (400 MHz, C_6D_6) δ 12.14 (s, 1H, $^{1}J_{CH}$ = 140.1 Hz, $C\underline{H}Ph$), 10.45 (s, 1H, $^{1}J_{CH}$ = 118.9 Hz, $C\underline{H}Ph$), 7.05 – 6.16 (m, 34H, $\underline{Ar}$), 5.90 (d, *J* = 5.0 Hz, 4H, IMes backbone), 3.24 – 3.13 (m, 2H, CH_2Ph), 2.71 – 2.58 (m, 2H, $C\underline{H_2}Ph$), 2.12 (d, *J* = 3.9 Hz, 12H, Mes), 2.02 (s, 6H, Mes), 1.98 (d, *J* = 5.3 Hz, 12H, Mes), 1.89 (s, 6H, Mes), 1.57 (d, *J* = 1.6 Hz, 18H, $O^t\underline{Bu}$), **^{13}C** NMR (**both isomers**) (101 MHz, CD_2Cl_2) δ 278.9 ($\underline{C}HPh$), 266.9 ($\underline{C}HPh$), 200.8 ($N\underline{C}N$), 199.5 ($N\underline{C}N$), 156.7 (Ar), 154.5 (Ar), 150.9 (Ar), 150.9 (Ar), 142.5 (Ar), 140.6 (Ar), 139.1 (Ar), 138.9 (Ar), 137.6 (Ar), 137.3 (Ar), 136.3 (Ar), 135.9 (Ar), 135.6 (Ar), 135.4 (Ar), 135.0 (Ar), 132.4 (Ar), 131.4 (Ar), 131.0 (Ar), 130.8 (Ar), 130.2 (Ar), 129.7 (Ar), 129.3 (Ar), 128.7 (Ar), 128.0 (Ar), 127.6 (Ar), 127.2 (Ar), 127.0 (Ar), 126.6 (Ar), 126.4 (Ar), 126.3 (Ar), 125.7 (Ar), 125.4 (Ar), 125.0 (Ar), 123.8 (Ar), 123.6 (Ar), 122.4 (Ar), 119.4 (IMes backbone), 119.2 (IMes backbone), 81.0 ($\underline{C}Me_3$), 80.5 ($\underline{C}Me_3$), 65.5 ($\underline{C}H_2Ph$), 64.3 ($\underline{C}H_2Ph$), 59.0, 32.7, 32.6, 31.4 ($C\underline{Me_3}$), 31.2 ($C\underline{Me_3}$), 21.4, 21.4, 19.2, 19.2, 19.1, 18.7; Anal. Calcd for $C_{45}H_{49}Cl_2N_3OW$: C, 59.88; H, 5.47; N, 4.66. Found: C, 59.86; H, 5.499; N, 4.66.

W(*N*-2,6-dichlorophenyl)(CHPh)(1,3-diisopropylimidazol-2-ylidene)(CH_2Ph)(O^tBu)

1,3-Diisopropylimidazol-2-ylidene (214 mg, 1.41 mmol, 1.50 equiv) and W(*N*-2,6-dichlorophenyl)$(CH_2Ph)_2(O^tBu)_2$ (630 mg, 947 µmol, 1.00 equiv) were weighed in a vial and dissolved in benzene (6 mL). The reaction mixture was stirred at room temperature for 1 h. Then the solvent was evaporated *in vacuo* and then co-evaporated with pentane (3 x 4 mL). Subsequently, the product was washed with a small amount of pentane (3 x 4 mL) to furnish the product as a beige powder (490 mg, 70%). **^{1}H** NMR (**major isomer**) (400 MHz, C_6D_6) δ 11.58 (s, 1H, $C\underline{H}Ph$), 7.54 – 7.47 (m, 3H, Ar), 7.36 – 7.32 (m, 2H, Ar), 7.14 – 7.08 (m, 3H, Ar), 6.99 (d, *J* = 8.0 Hz, 2H,

Ar), 6.80 – 6.73 (m, 2H, Ar), 6.23 (t, *J* = 8.0 Hz, 1H, Ar), 6.18 (s, 2H, NHC backbone), 4.78 (hept, *J* = 6.6 Hz, 2H, C<u>H</u>(CH_3)$_2$), 3.73 (d, *J* = 9.9 Hz, 1H, C<u>H</u>$_2$Ph), 3.38 (d, *J* = 9.8 Hz, 1H, C<u>H</u>$_2$Ph), 1.33 (s, 9H, O^t<u>Bu</u>), 0.97 (d, *J* = 6.7 Hz, 6H, CH(C<u>H</u>$_3$)$_2$), 0.85 (d, *J* = 6.7 Hz, 6H, CH(C<u>H</u>$_3$)$_2$). ^{1}H NMR (**minor isomer**) (400 MHz, C_6D_6) δ 12.66 (s, 1H, C<u>H</u>Ph), 7.65 (d, *J* = 7.4 Hz, 2H, Ar), 7.42 – 7.36 (m, 3H, Ar), 7.25 – 7.19 (m, 3H, Ar), 7.04 (d, *J* = 8.0 Hz, 2H, Ar), 6.70 – 6.62 (m, 2H, Ar), 6.31 (t, *J* = 8.0 Hz, 1H, Ar), 6.17 (s, 2H, NHC backbone), 5.04 (hept, *J* = 6.6 Hz, 2H, C<u>H</u>(CH_3)$_2$), 3.08 (d, *J* = 11.3 Hz, 1H, C<u>H</u>$_2$Ph), 2.96 (d, *J* = 11.3 Hz, 1H, C<u>H</u>$_2$Ph), 1.31 (s, 9H, O^t<u>Bu</u>), 0.79 (d, *J* = 6.7 Hz, 6H, CH(C<u>H</u>$_3$)$_2$), 0.76 (d, *J* = 6.7 Hz, 6H, CH(C<u>H</u>$_3$)$_2$), ^{13}**C** NMR (**both isomers**) (101 MHz, CD_2Cl_2) δ 271.4 (<u>C</u>HPh), 253.6 (<u>C</u>HPh), 194.0 (N<u>C</u>N), 193.8 (N<u>C</u>N), 156.6 (Ar), 152.5 (Ar), 151.6 (Ar), 151.5 (Ar), 146.2 (Ar), 142.4 (Ar), 132.3 (Ar), 132.1 (Ar), 129.4 (Ar), 129.2 (Ar), 128.8 (Ar), 128.6 (Ar), 128.0 (Ar), 127.9 (Ar), 127.3 (Ar), 127.1 (Ar), 126.9 (Ar), 126.5 (Ar), 126.4 (Ar), 122.7 (Ar), 122.7 (Ar), 120.9 (Ar), 120.1 (Ar), 119.8 (Ar), 117.2 (IPr backbone), 117.1 (IPr backbone), 80.1 (<u>C</u>Me$_3$), 78.9 (<u>C</u>Me$_3$), 67.0 (<u>C</u>H$_2$Ph), 57.6 (<u>C</u>H$_2$Ph), 53.0 (<u>C</u>H(CH_3)$_2$), 51.6 (<u>C</u>H(CH_3)$_2$), 32.8 (C<u>Me</u>$_3$), 32.2 (C<u>Me</u>$_3$), 23.7 (CH(<u>C</u>H$_3$)$_2$), 23.6 (CH(<u>C</u>H$_3$)$_2$), 23.5 (CH(<u>C</u>H$_3$)$_2$), 23.1 (CH(<u>C</u>H$_3$)$_2$); Anal. Calcd for $C_{33}H_{41}Cl_2N_3OW$: C, 52.82; H, 5.51; N, 5.60. Found: C, 52.45; H, 5.521; N, 5.55.

W(*N*-2,6-dichlorophenyl)(CHPh)(1,3-dicyclohexylimidazol-2-ylidene)(CH_2Ph)(O^{t}Bu)

1,3-Dicyclohexylimidazol-2-ylidene (34.6 mg, 149 µmol, 1.00 equiv) and W(*N*-2,6-dichlorophenyl)(CH_2CMe_2Ph)$_2$(O^{t}Bu)$_2$ (100 mg, 149 µmol, 1.00 equiv) were weighed in a vial and dissolved in benzene (2 mL). The reaction mixture was stirred at room temperature for 30 min. Then the solvent was evaporated *in vacuo* and co-evaporated with pentane (3 x 3 mL). Subsequently, the product was washed with a small amount of pentane (3 x 3 mL) to furnish the product as a beige powder (90 mg, 73%). The product was recrystallized form dichloromethane/pentane to yield off-white crystals for X-ray analysis. ^{1}H NMR (**both isomers**) (400 MHz, C_6D_6) δ 12.62 (s, 1H, $^1J_{CH}$ = 141.3 Hz C<u>H</u>Ph), 11.61 (s, 1H, $^1J_{CH}$ = 124.1 Hz C<u>H</u>Ph), 7.70 (d, *J* = 7.7 Hz, 2H, Ar), 7.50 – 7.35 (m, 5H, Ar), 7.26 – 7.20 (m, 3H, Ar), 7.16 – 7.11 (m, 2H, Ar), 7.07 – 6.88 (m, 10H, Ar), 6.81 – 6.74 (m, 1H, Ar), 6.70 – 6.59 (m, 1H, Ar), 6.42 – 6.19 (m, 6H, Ar), 4.68 (d, *J* = 47.3 Hz, 4H, NC<u>H</u>(CH_2)$_5$), 3.54 (d, *J* = 11.0 Hz, 1H, C<u>H</u>$_2$Ph), 3.37 (d, *J* = 10.9 Hz, 1H, C<u>H</u>$_2$Ph), 3.03-2.90 (m, 2H, C<u>H</u>$_2$Ph), 2.06 – 1.91 (m, 2H, Cy), 1.87 – 1.69 (m, 4H, Cy), 1.59 – 1.40 (m, 8H, Cy), 1.41 – 1.17 (m, 28H, Cy, O^t<u>Bu</u>), 1.15 – 0.95 (m, 10H, Cy), 0.91 – 0.71 (m, 6H, Cy); ^{13}C NMR (**both isomers**) (101 MHz, CD_2Cl_2) δ 271.5 (<u>C</u>HPh), 258.1 (<u>C</u>HPh), 194.8 (N<u>C</u>N), 194.3 (N<u>C</u>N), 144.7 (Ar), 142.4 (Ar), 132.2 (Ar), 131.9 (Ar), 129.9 (Ar), 129.4 (Ar), 129.0 (Ar), 128.8 (Ar), 128.6 (Ar), 128.2 (Ar), 127.3 (Ar), 127.1 (Ar), 127.0 (Ar), 126.9 (Ar), 126.3 (Ar), 122.9 (Ar), 122.7 (Ar), 120.5 (Ar), 120.1 (Ar), 117.9 (ICy backbone), 117.4 (ICy backbone), 80.3 (<u>C</u>Me$_3$), 79.3 (<u>C</u>Me$_3$), 68.1 (<u>C</u>H$_2$Ph), 60.0 32.2 (C<u>Me</u>$_3$), 25.8 (Cy), 25.6 (Cy), 24.8 (Cy), 24.7 (Cy); Anal. Calcd for $C_{39}H_{49}Cl_2N_3OW$: C, 56.40; H, 5.95; N, 5.06. Found: C, 56.57; H, 5.969; N, 4.99.

W(*N*-2,6-dimethylphenyl)(CHPh)(1,3-dimesitylimidazol-2-ylidene)(CH_2Ph)(O^tBu)

1,3-Dimesitylimidazol-2-ylidene (145 mg, 475 µmol, 1.00 equiv) and W(*N*-2,6-dimethylphenyl)$(CH_2Ph)_2(O^tBu)_2$ (300 mg, 158 µmol, 1.00 equiv) were weighed in a vial and dissolved in benzene (3 mL). The reaction mixture was stirred at room temperature for 5 h. Then the solvent was evaporated *in vacuo* and pentane was added. The reaction as stirred for another 36 h, then the solvent was evaporated *in vacuo* and then co-evaporated with pentane (3 x 2 mL). Subsequently, the product was washed with a small amount of cold pentane (3 x 2 mL) to furnish the product as a yellow powder (236 mg, 58%). **^{1}H** NMR (**major isomer**) (400 MHz, C_6D_6) δ 11.82 (s, 1H, CHPh, $^1J_{CH}$ = 137,9 Hz), 7.13 – 6.69 (m, 10H, Ar), 6.60 (t, *J* = 7.2 Hz, 1H, Ar), 6.54 (s, 2H, Ar), 6.50 – 6.40 (m, 2H, Ar), 6.25 (s, 2H, Ar), 5.86 (s, 2H, IMes backbone), 2.59 (d, *J* = 10.7 Hz, 1H, CH_2Ph), 2.31 (d, *J* = 10.5 Hz, 1H, CH_2Ph), 2.22 (s, 3H,), 2.12 (s, 3H,), 2.03 (d, *J* = 17.8 Hz, 12H, Mes), 1.83 (s, 3H), 1.76 (s, 3H), 1.59 (s, 9H, O^tBu); **minor isomer < 10%, not determined**; **^{13}C** NMR (101 MHz, CD2Cl2) δ 276.9 (CHPh), 200.1 (NCN), 157.0 (Ar), 155.1 (Ar), 141.3 (Ar), 139.0 (Ar), 137.4 (Ar), 135.7 (Ar), 134.7 (Ar), 130.7 (Ar), 130.2 (Ar), 129.0 (Ar), 126.4 (Ar), 126.1 (Ar), 125.9 (Ar), 125.7 (Ar), 123.8 (Ar), 123.1 (Ar), 119.2 (IMes backbone), 80.0 (CMe_3), 63.8 (CH_2Ph), 54.0, 33.1 (CMe_3), 21.4, 20.1, 19.1, 18.8, 18.4; **minor isomer not determined**; Anal. Calcd for $C_{47}H_{55}N_3OW$: C, 65.50; H, 6.43; N, 4.88. Found: C, 65.20; H, 6.571; N, 4.91.

W(*N*-2,6-dimethylphenyl)(CHPh)(1,3-diisopropylimidazol-2-ylidene)(CH_2Ph)(O^tBu)

1,3-Diisopropylimidazol-2-ylidene (36.2 mg, 238 µmol, 1.50 equiv) and W(*N*-2,6-dimethylphenyl)$(CH_2Ph)_2(O^tBu)_2$ (100 mg, 158 µmol, 1.00 equiv) were weighed in a vial and dissolved in benzene (1.5 mL). The reaction mixture was stirred at room temperature for 3 h. Then the solvent was evaporated *in vacuo* and co-evaporated with pentane (3 x 2 mL). Subsequently, the product was washed with a small amount of pentane (3 x 2 mL) to furnish the product as a light-orange microcrystalline powder (90.3 mg, 80%). **^{1}H** NMR (**major Isomer**) (400 MHz, C_6D_6) δ 11.74 (s, 1H, CHPh), 7.52 – 7.46 (m, 2H, Ar), 7.40 – 7.33 (m, 2H, Ar), 7.13 (d, *J* = 7.8 Hz, 2H, Ar), 7.03 – 6.98 (m, 2H, Ar), 6.98 – 6.93 (m, 2H, Ar), 6.91 – 6.81 (m, 3H, Ar), 6.78 (t, *J* = 7.5 Hz, 1H, Ar), 6.15 (s, 2H, IPr Backbone), 4.81 (hept, *J* = 6.6 Hz, 2H, $CH(CH_3)_2$), 3.33 (d, *J* = 10.9 Hz, 1H, CH_2Ph), 3.17 (d, *J* = 10.9 Hz, 1H, CH_2Ph), 2.26 (s, 6H, Me), 1.28 (s, 9H, O^tBu), 0.92 (d, *J* = 6.7 Hz, 6H, $CH(CH_3)_2$), 0.77 (d, *J* = 6.7 Hz, 6H, $CH(CH_3)_2$); **^{1}H** NMR (**minor isomer**) (400 MHz, C_6D_6) δ 12.38 (s, 1H, CHPh), 7.70 – 7.61 (m, 2H, Ar), 7.43 – 7.39 (m, 1H, Ar), 7.06 – 7.01 (m, 2H, Ar), 6.91 – 6.85 (m, 3H, Ar), 6.82 – 6.74 (m, 4H, Ar), 6.69 – 6.58 (m, 1H, Ar), 6.15 (s, 2H, IPr Backbone), 4.93 (hept, *J* = 6.7 Hz, 2H, $CH(CH_3)_2$), 2.93 (d, *J* = 11.5 Hz, 1H, CH_2Ph), 2.78 (d, *J* = 11.5 Hz, 1H, CH_2Ph), 2.58 (s, 6H, Me), 1.33 (s, 9H, O^tBu), 0.88 (d, *J* = 6.9 Hz, 6H, $CH(CH_3)_2$), 0.71 (d, *J* = 6.7 Hz, 6H, $CH(CH_3)_2$); **^{13}C** NMR (**major isomer**) (101 MHz, CD_2Cl_2) δ 253.8 (CHPh), 195.1 (NCN), 153.9 (Ar), 147.2 (Ar), 134.7 (Ar), 129.8 (Ar), 129.0 (Ar), 128.0 (Ar), 127.5 (Ar), 127.2 (Ar), 126.3 (Ar), 122.7 (Ar), 121.0 (Ar), 117.1 (IPr Backbone), 78.0 (CMe_3), 58.0 (CH_2Ph), 52.8 ($CH(CH_3)_2$), 32.4 (CMe_3), 23.9 ($CH(CH_3)_2$), 23.5

($CH(\underline{C}H_3)_2$), 19.4 (Me); **^{13}C** NMR (**minor isomer**) (101 MHz, CD_2Cl_2) δ 269.4 ($\underline{C}$HPh), 195.2 (N$\underline{C}$N), 156.7 (Ar), 155.9 (Ar), 143.2 (Ar), 134.6 (Ar), 128.5 (Ar), 127.9 (Ar), 127.6 (Ar), 127.0 (Ar), 126.6 (Ar), 126.5 (Ar), 123.2 (Ar), 120.1 (Ar), 117.2 (IPr Backbone), 79.3 ($\underline{C}Me_3$), 66.1 ($\underline{C}H_2Ph$), 51.4 ($\underline{C}H(CH_3)_2$), 33.1 ($C\underline{Me_3}$), 23.0 ($CH(\underline{C}H_3)_2$), 22.9 ($CH(\underline{C}H_3)_2$), 19.4 (Me); Anal. Calcd for $C_{35}H_{47}N_3OW$: C, 59.24; H, 6.68; N, 5.92. Found: C, 58.98; H, 6.711; N, 5.88.

W(*N*-2,6-dimethylphenyl)(CHPh)(1,3-dicyclohexylimidazol-2-ylidene)(CH_2Ph)(O^{t}Bu)

1,3-Dicyclohexylimidazol-2-ylidene (88.3 mg, 380 µmol, 1.20 equiv) and W(*N*-2,6-dimethylphenyl)(CH_2Ph)$_2$(O^{t}Bu)$_2$ (200 mg, 317 µmol, 1.00 equiv) were weighed in a vial and dissolved in benzene (2 mL). The reaction mixture was stirred at room temperature for 75 min. Then the solvent was evaporated *in vacuo* and then co-evaporated with pentane (3 x 3 mL). Subsequently, the product was washed with a small amount of pentane (3 x 3 mL) to furnish the product as a yellow fluffy powder (293 mg, 96%). **^{1}H** NMR (**major isomer**) (400 MHz, C_6D_6) δ 11.76 (s, 1H, C$\underline{H}$Ph), 7.56 – 7.47 (m, 4H, Ar), 7.26 – 7.18 (m, 2H, Ar), 6.98 – 6.92 (m, 3H, Ar), 6.91 – 6.81 (m, 3H, Ar), 6.77 (t, *J* = 7.5 Hz, 1H, Ar), 6.29 (s, 2H, ICy backbone), 4.98 – 4.30 (m, 2H, $NC\underline{H}(CH_2)_5$), 3.41 (d, *J* = 11.8 Hz, 1H, C$\underline{H}$Ph), 2.99 (d, *J* = 11.8 Hz, 1H, C$\underline{H}$Ph), 2.32 (s, 6H, Me), 1.76 – 1.64 (m, 2H, Cy), 1.62 – 1.40 (m, 6H, Cy), 1.37 – 1.27 (m, 2H, Cy), 1.25 (s, 9H, O$^t\underline{Bu}$), 1.22 – 1.12 (m, 2H, Cy), 1.06 – 0.90 (m, 4H, Cy), 0.91 – 0.69 (m, 4H, Cy); **^{1}H** NMR (**minor isomer**) (400 MHz, C_6D_6) δ 12.37 (s, 1H, C$\underline{H}$Ph), 7.69 (d, *J* = 7.7 Hz, 2H, Ar), 7.56 – 7.44 (m, 3H, Ar), 7.41 (dd, *J* = 8.4, 7.3 Hz, 2H, Ar), 7.15 – 7.11 (m, 2H, Ar), 7.04 – 6.97 (m, 2H, Ar), 6.65 (t, *J* = 7.2 Hz, 1H, Ar), 6.35 (s, 2H, ICy Backbone), 4.36 – 4.04 (m, 2H, $NC\underline{H}(CH_2)_5$), 2.87 – 2.72 (m, 2H, C$\underline{H}$Ph), 2.57 (s, 6H, Me), 2.00 – 1.80 (m, 2H, Cy), 1.75 – 1.66 (m, 2H, Cy), 1.59 – 1.40 (m, 4H, Cy), 1.39 (s, 9H, O$^t\underline{Bu}$), 1.36 – 1.28 (m, 2H, Cy), 1.22 – 1.14 (m, 2H, Cy), 1.06 – 0.91 (m, 4H, Cy), 0.90 – 0.71 (m, 2H, Cy), 0.66 – 0.54 (m, 2H, Cy); **^{13}C** NMR (**major isomer**) (101 MHz, CD_2Cl_2) δ 255.6 ($\underline{C}$HPh), 195.7 (N$\underline{C}$N), 155.6 (Ar), 154.9 (Ar), 146.1 (Ar), 134.5 (Ar), 129.9 (Ar), 129.3 (Ar), 128.1 (Ar), 127.5 (Ar), 127.1 (Ar), 126.6 (Ar), 122.7 (Ar), 120.9 (Ar), 117.4 (ICy Backbone), 78.1 ($\underline{C}Me_3$), 58.9 ($\underline{C}H_2Ph$), 57.9 ($N\underline{C}H(CH_2)_5$), 34.6 (Cy), 32.3 ($C\underline{Me_3}$), 26.1 (Cy), 25.8 (Cy), 19.8 (Me); **^{13}C** NMR (**minor isomer**) (101 MHz, CD_2Cl_2) δ 269.0 ($\underline{C}$HPh), 195.4 (N$\underline{C}$N), 156.4 (Ar), 156.0 (Ar), 143.1 (Ar), 134.6 (Ar), 129.1 (Ar), 128.8 (Ar), 128.3 (Ar), 127.8 (Ar), 126.4 (Ar), 123.3 (Ar), 120.1 (Ar), 119.5 (Ar), 117.9 (ICy Backbone), 79.3 ($\underline{C}Me_3$), 66.9 ($\underline{C}H_2Ph$), 60.3 ($N\underline{C}H(CH_2)_5$), 34.7 (Cy), 34.1 ($C\underline{Me_3}$), 22.9 (Cy), 20.2 (Cy), 14.4 (Me); Anal. Calcd for $C_{35}H_{47}N_3OW$: C, 59.24; H, 6.68; N, 5.92. Found: C, 58.98; H, 6.711; N, 5.88; Anal. Calcd for $C_{41}H_{55}N_3OW$: C, 62.35; H, 7.02; N, 5.32. Found: C, 62.35; H, 6.117; N, 5.27.

W(*N*-2,6-diisopropylphenyl)(CHPh)(1,3-diisopropylimidazol-2-ylidene)(CH_2Ph)(O^{t}Bu)

1,3-Diisopropylimidazol-2-ylidene (33.2 mg, 218 µmol, 1.50 equiv) and W(*N*-2,6-diisopropylphenyl)(CH_2Ph)$_2$(O^{t}Bu)$_2$ (100 mg, 145 µmol, 1.00 equiv) were weighed in a

vial and dissolved in benzene (1.5 mL). The reaction mixture was stirred at room temperature for 3 h. Then the solvent was evaporated *in vacuo* and co-evaporated with pentane (3 x 2 mL). Subsequently, the product was washed with a small amount of pentane (3 x 2 mL) to furnish the product as a beige microcrystalline powder (97.2 mg, 87%). **^{1}H** NMR (**major isomer**) (400 MHz, C_6D_6) δ 11.79 (s, 1H, $C\underline{H}Ph$), 7.53 (d, *J* = 7.7 Hz, 2H, Ar), 7.41 (d, *J* = 7.9 Hz, 2H, Ar), 7.25 – 7.19 (m, 2H, Ar), 7.15 – 7.07 (m, 4H, Ar), 6.99 – 6.94 (m, 1H, Ar), 6.90 – 6.82 (m, 3H, Ar), 6.14 (s, 2H, IPr Backbone), 4.96 – 4.77 (m, 2H, $C\underline{H}(CH_3)_2$), 4.11 – 3.95 (m, 2H, $C\underline{H}(CH_3)_2$), 3.45 (d, *J* = 11.7 Hz, 1H, $C\underline{H_2}Ph$), 2.88 (d, *J* = 11.7 Hz, 1H, $C\underline{H_2}Ph$), 1.17 (s, 9H, $O^t\underline{Bu}$), 1.14 (d, *J* = 6.3 Hz, 6H, $CH(C\underline{H_3})_2$), 0.99 (d, *J* = 6.7 Hz, 6H, $CH(C\underline{H_3})_2$), 0.90 – 0.75 (m, 12H, $CH(C\underline{H_3})_2$); ^{1}H NMR (**minor isomer**) (400 MHz, C_6D_6) δ 12.36 (s, 1H, $C\underline{H}Ph$), 7.39 – 7.31 (m, 2H, Ar), 7.26 – 7.06 (m, 5H, Ar), 7.06 – 7.00 (m, 3H, Ar), 6.91 – 6.82 (m, 2H, Ar), 6.70 (tt, *J* = 7.3, 1.3 Hz, 1H, Ar), 6.15 (s, 2H, IPr Backbone), 4.97 – 4.71 (m, 2H, $C\underline{H}(CH_3)_2$), 4.22 (hept, *J* = 7.0 Hz, 2H, $C\underline{H}(CH_3)_2$), 3.15 (d, *J* = 11.7 Hz, 1H, $C\underline{H_2}Ph$), 2.67 (d, *J* = 11.7 Hz, 1H, $C\underline{H_2}Ph$), 1.35 (d, *J* = 6.9 Hz, 6H, $CH(C\underline{H_3})_2$), 1.31 (s, 9H, $O^t\underline{Bu}$), 1.28 (d, *J* = 6.8 Hz, 6H, $CH(C\underline{H_3})_2$), 0.88 – 0.84 (m, 6H, $CH(C\underline{H_3})_2$), 0.69 (d, *J* = 6.6 Hz, 6H, $CH(C\underline{H_3})_2$); **^{13}C** NMR (**major isomer**) (101 MHz, CD_2Cl_2) δ 257.5 ($\underline{C}HPh$), 195.7 ($N\underline{C}N$), 155.2 (Ar), 152.7 (Ar), 146.8 (Ar), 145.1 (Ar), 130.0 (Ar), 129.2 (Ar), 127.9 (Ar), 127.1 (Ar), 126.2 (Ar), 123.5 (Ar), 123.4 (Ar), 121.1 (Ar), 117.1 (IPr Backbone), 78.2 ($\underline{C}Me_3$), 58.5 ($\underline{C}H_2Ph$), 52.9 ($\underline{C}H(CH_3)_2$), 32.2 ($C\underline{Me_3}$), 27.4 ($\underline{C}H(CH_3)_2$), 24.2 ($\underline{C}H(CH_3)_2$), 24.0 ($\underline{C}H(CH_3)_2$), 23.2 ($\underline{C}H(CH_3)_2$); **^{13}C** NMR (**minor isomer**) (101 MHz, CD_2Cl_2) δ 269.5 ($\underline{C}HPh$), 195.0 ($N\underline{C}N$), 155.6 (Ar), 153.6 (Ar), 144.5 (Ar), 143.0 (Ar), 128.8 (Ar), 128.3 (Ar), 127.7 (Ar), 126.7 (Ar), 126.4 (Ar), 124.0 (Ar), 123.1 (Ar), 120.5 (Ar), 117.3 (IPr Backbone), 79.3 ($\underline{C}Me_3$), 63.6 ($\underline{C}H_2Ph$), 51.6 ($\underline{C}H(CH_3)_2$), 33.0 ($C\underline{Me_3}$), 28.6 ($\underline{C}H(CH_3)_2$), 25.5 ($\underline{C}H(CH_3)_2$), 24.7 ($\underline{C}H(CH_3)_2$), 23.0 ($\underline{C}H(CH_3)_2$); Anal. Calcd for $C_{39}H_{55}N_3OW$: C, 61.17; H, 7.24; N, 5.49. Found: C, 60.85; H, 7.190; N, 5.45.

W(*N*-2,6-diisopropylphenyl)(CHPh)(1,3-dicyclohexylimidazol-2-ylidene)(CH_2Ph)(O^tBu)

1,3-Dicyclohexylimidazol-2-ylidene (142 mg, 611 µmol, 1.20 equiv) and W(*N*-2,6-diisopropylphenyl)(CH_2Ph)$_2$(O^tBu)$_2$ (350 mg, 317 µmol, 1.00 equiv) were weighed in a vial and dissolved in benzene (3 mL). The reaction mixture was stirred at room temperature for 2.5 h. Then the solvent was evaporated *in vacuo* and co-evaporated with pentane (3 x 8 mL). Subsequently, the product was washed with a small amount of pentane (3 x 8 mL) to furnish the product as a fluffy off-white powder (297 mg, 69%). **^{1}H** NMR (**major isomer**) (400 MHz, CD_2Cl_2) δ 11.46 (s, 1H, $C\underline{H}Ph$), 7.44 – 7.36 (m, 2H, Ar), 7.24 – 7.14 (m, 2H, Ar), 7.12 – 6.82 (m, 10H, Ar, ICy Backbone), 6.68 – 6.62 (m, 1H, Ar), 4.81 – 4.64 (m, 1H, $NC\underline{H}(CH_2)_5$), 4.21 – 3.99 (m, 1H, $NC\underline{H}(CH_2)_5$), 3.93 – 3.53 (m, 2H, $C\underline{H}(CH_3)_2$), 2.97 (d, *J* = 11.7 Hz, 1H, $C\underline{H_2}Ph$), 2.29 – 2.18 (m, 2H, Cy), 2.15 (d, *J* = 11.8 Hz, 1H, $C\underline{H_2}Ph$), 1.96 – 1.43 (m, 8H, cy), 1.43 – 0.94 (m, 22H, Cy, $CH(C\underline{H_3})_2$), 0.90 (s, 9H, $O^t\underline{Bu}$); **minor isomer < 15%**, **not determined**; **^{13}C** NMR (**major isomer**) (101 MHz, CD_2Cl_2) δ 257.2, 196.0, 155.4, 152.7, 146.1, 145.3, 130.5,

129.2, 127.9, 127.1, 126.6, 123.4, 121.2, 117.7, 117.5, 78.2, 60.9, 59.7, 58.2, 54.0, 35.0, 34.6, 34.3, 34.0, 32.9, 31.9, 26.4, 26.1, 25.9, 25.6; **minor isomer < 15%**, **not determined**; Anal. Calcd for $C_{45}H_{63}NO_3W$: C, 63.90; H, 7.51; N, 4.97. Found: C, 63.49; H, 7.503; N, 4.95.

Reversibility of α-H Abstraction

A solution of *t*BuOH (27.1 mg, 366 µmol) in C_6D_6 (1 mL) was prepared. Three scintillation vials were equipped with W(*N*-2,6-dichlorophenyl)(CHPh)(1,3-dimesitylimidazol-2-ylidene)(CH_2Ph)(O*t*Bu) (33 mg, 36.6 µmol, 1.00 equiv). To each vial, C_6D_6 (1mL) and then 100 µL (1.00 equiv), 200 µL (2.00 equiv) or 300 µL (3.00 equiv) of the *t*BuOH solution was added. After stirring for 100 min, ^{1}H NMR spectra were recorded.

The first spectrum shows the isolated alkylidene complex **2** (spectrum 5, Figure S1) to which 1, 2 or 3 equiv of *t*BuOH were added (spectra 2,3 and 4, Figure S1). A build-up of peaks is apparent that can be assigned to W(*N*-2,6-dichlorophenyl)(CH_2Ph)$_2$(O*t*Bu)$_2$ **1** (spectrum 1, Figure S1).

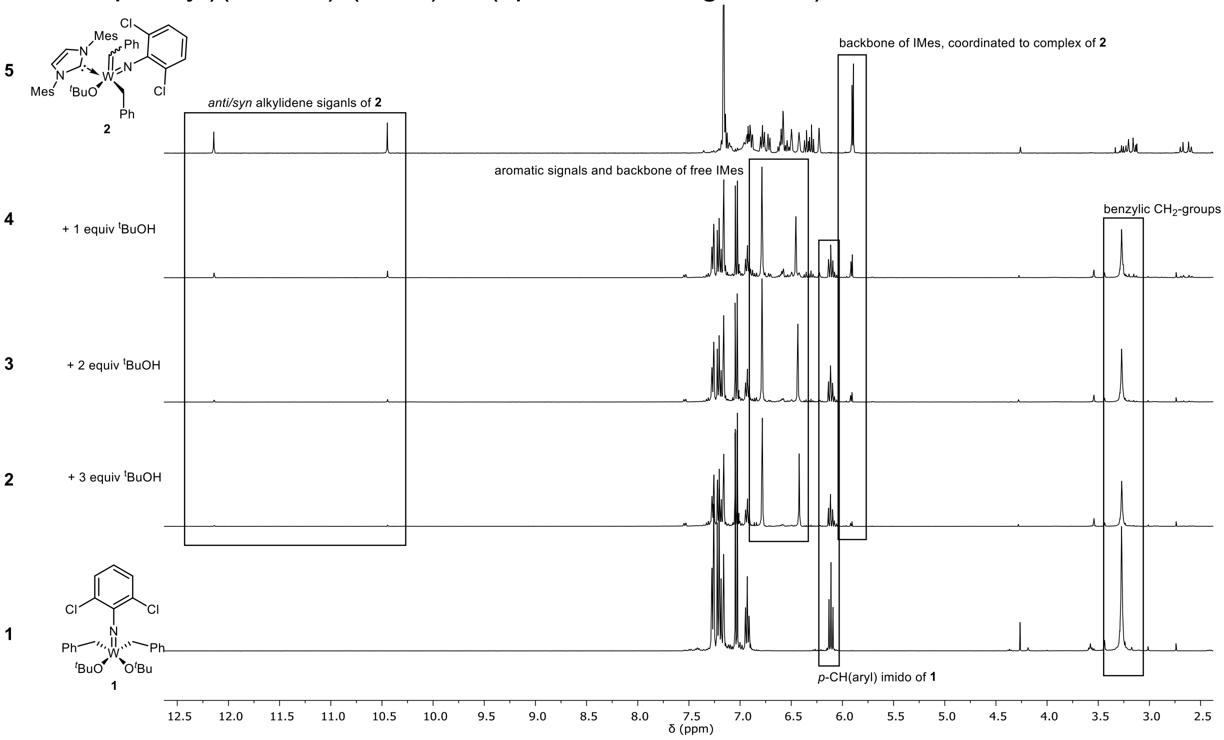

Figure S1. Reversibility of α-H Abstraction.

General Procedures for Kinetic Measurements

W(*N*Ar)(alkyl)$_2$(O*t*Bu)$_2$ (approximately 370 µmol, 1.00 equiv) were dissolved in C_6D_6 (5 mL). The NHC (approximately 370 µmol, 1.00 equiv) and cyclooctane (approximately 370 µmol, 1.0 equiv, internal standard) were dissolved in C_6D_6 (5 mL). The two solutions (500 µL each) were mixed either in a scintillation vial in a dry box or directly in an NMR tube quipped with a septum using a syringe. ^{1}H NMR spectra were recorded in intervals of 2 or 5 minutes. Integrals were normalized with respect to the internal standard. For monitoring of the kinetics, the decreasing integrals of the benzylic CH_2-group of W(*N*Ar)(CH_2Ph)$_2$(O*t*Bu)$_2$ and the increasing integrals of the NHC backbone of W(*N*Ar)(CHPh)(NHC)(CH_2Ph)$_2$(O*t*Bu) were evaluated. For the deuterated

complex W(*N*Ar)($CD_2C_6D_5$)$_2$(O^{t}Bu)$_2$, the decrease of the integrals of the two *m*-H of the imido-ligand were monitored.

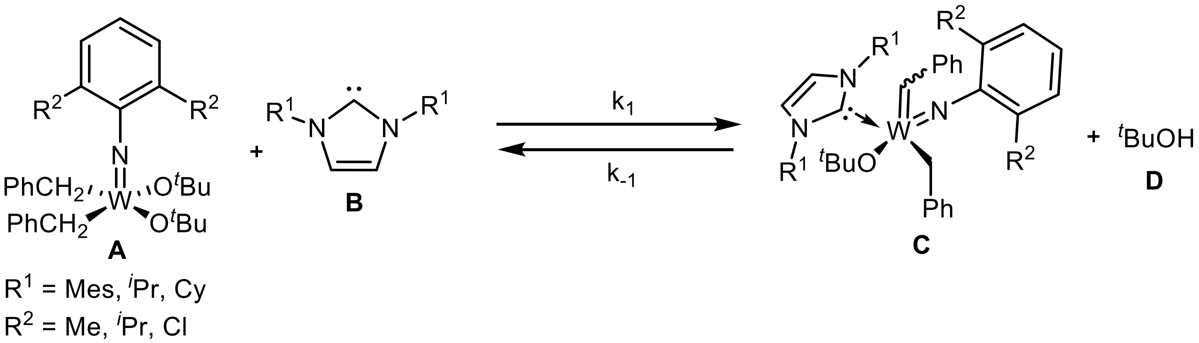

Scheme S1. Equilibrium formation of a W imido alkylidene NHC complex.

A 2nd-order rate law for an equilibrium reaction was applied:

For $[A] = [B]$ and $[C] = [D]$:

$$\frac{-\,\mathrm{d}[A]}{\mathrm{d}t} = k_1[A]^2 - k_{-1}[C]^2 \quad \text{and} \quad K = \frac{[C]^2}{[A]^2} = \frac{k_1}{k_{-1}} \quad \text{and} \quad [C] = [A_0] - [A]$$

Which leads to following equation:

$$\frac{-\,\mathrm{d}[A]}{\mathrm{d}t} = k_1\left([A]^2 - \frac{1}{K}([A_0] - [A])^2\right)$$

Integration:

$$\int_{[A_0]}^{[A_t]} \frac{\mathrm{d}[A]}{[A]^2 - \frac{1}{K}([A_0] - [A])^2} = -k_1 \int_0^t \mathrm{d}t$$

Integrated 2nd-order rate law for equilibrium equation:

$$-\frac{\sqrt{K}\ln\left(\frac{\left|(\mathrm{K}-1)[A] - \left(\sqrt{K}-1\right)[A_0]\right|}{\left|(\mathrm{K}-1)[A] + \left(\sqrt{K}+1\right)[A_0]\right|}\right)}{2[A_0]} = k_1 t + c$$

The left part of the equation was plotted vs. time for each W imido dialkly complex. The slope of the regression line is the rate constant of the reaction. The uncertainty of the rate constant was calculated as the standard deviation of the slope of the regression line.

Kinetic Measurements

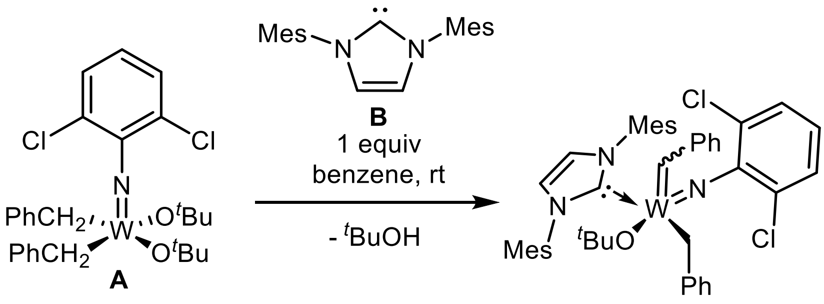

Scheme S2. Formation of **2**.

Initial concentrations:

[A] = 37.2 mM

[B] = 39.3 mM

[cyclooctane] = 36.7 mM

K = 0.269 (determined after 70 min)

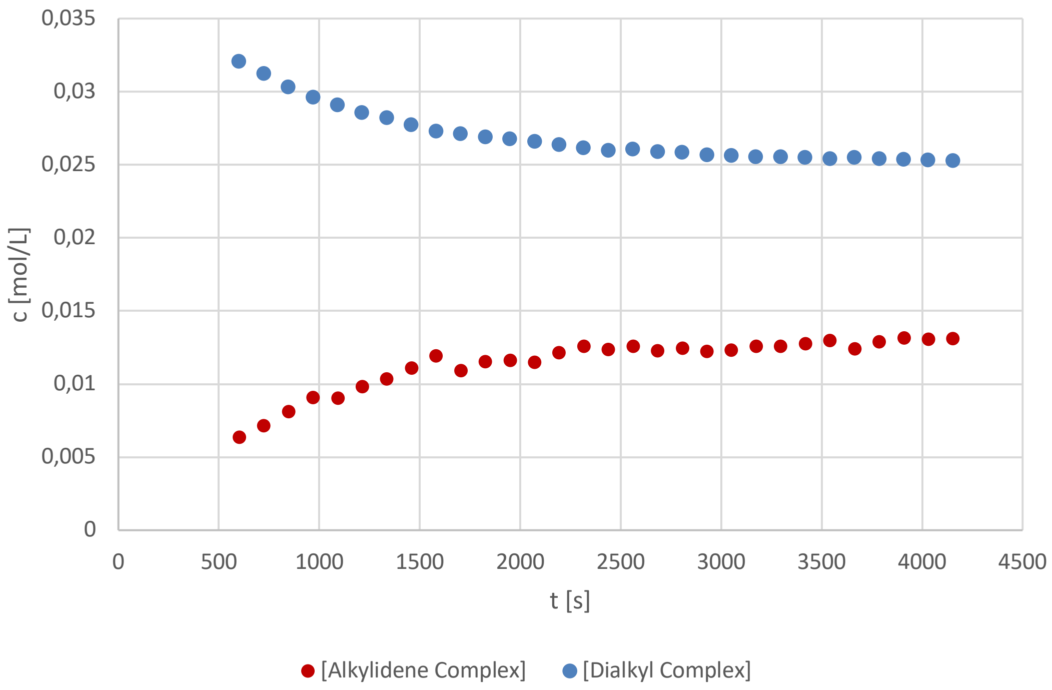

Figure S2. Formation of **2**; plot of concentrations of the W dialkyl and W alkylidene complex.

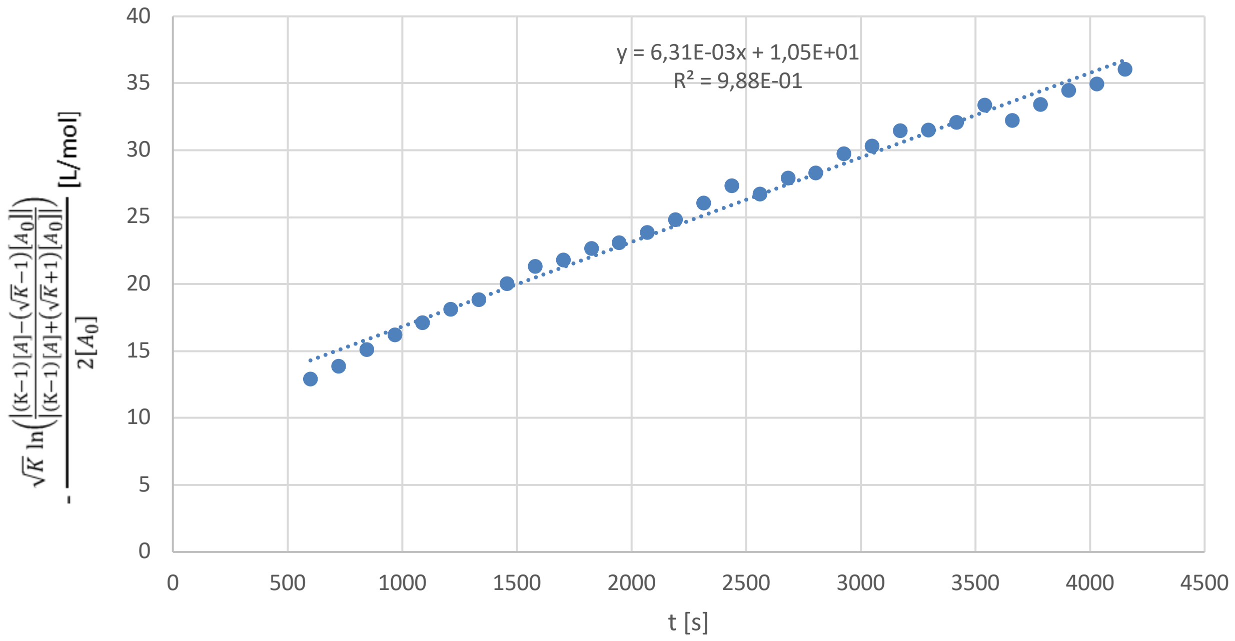

Figure S3. Formation of **2**; plot of $-\frac{\sqrt{K}\ln\left(\frac{|(K-1)[A]-(\sqrt{K}-1)[A_0]|}{|(K-1)[A]+(\sqrt{K}+1)[A_0]|}\right)}{2[A_0]}$ of the W dialkyl complex *vs.* time.

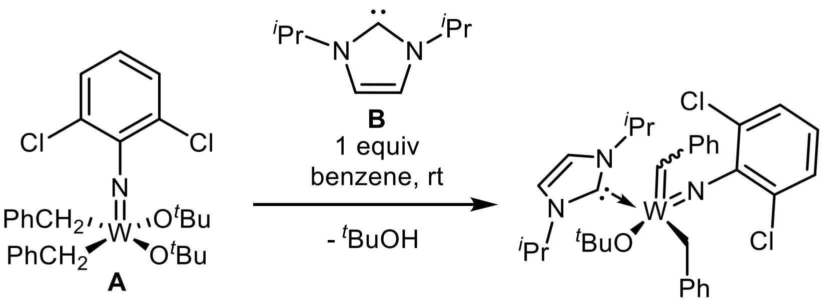

Scheme S3. Formation of **6**.

Initial concentrations:

[A] = 37.2 mM

[B] = 39.4 mM

[cyclooctane] = 33.9 mM

K = 102 (determined after 95 min)

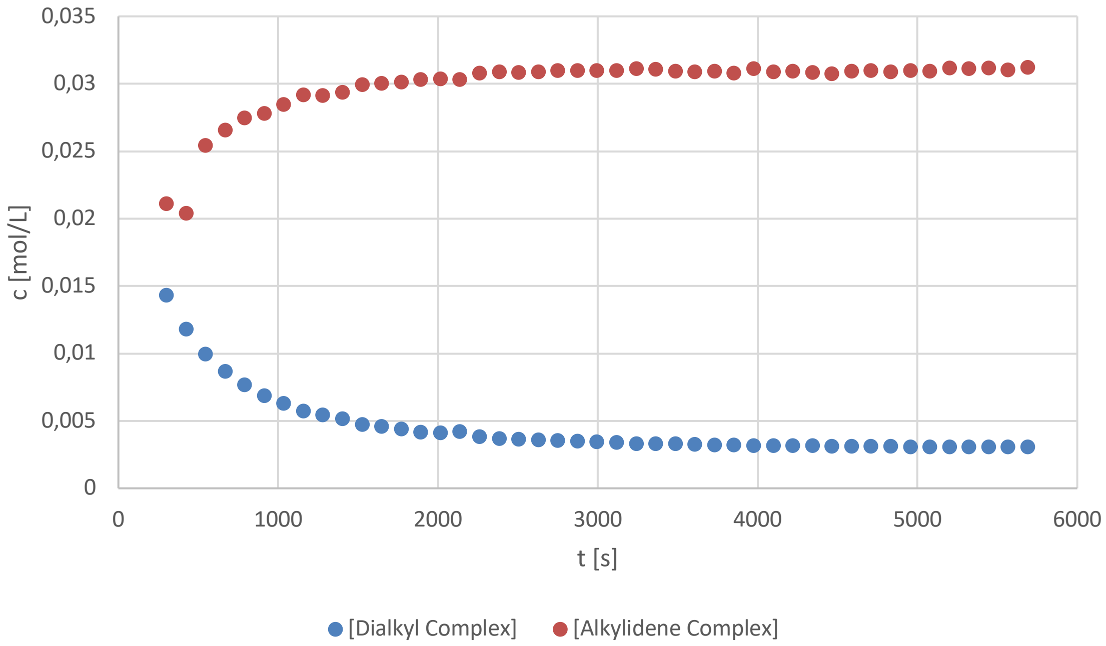

Figure S4. Formation of **6**; plot of concentrations of the W dialkyl and W alkylidene complex.

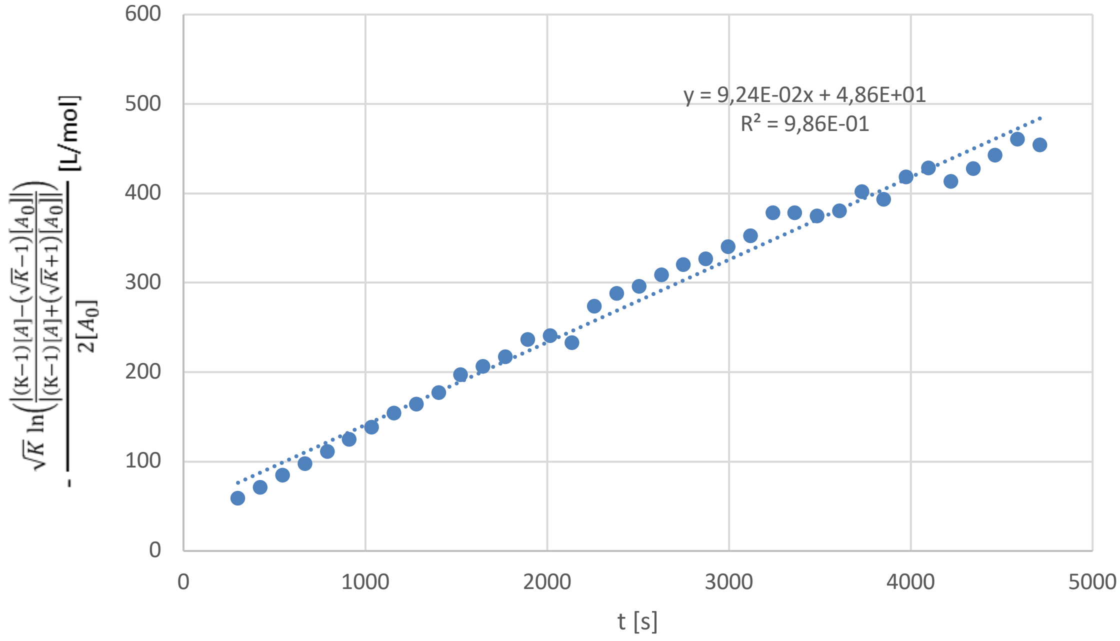

Figure S5. Formation of **6**; plot of $-\frac{\sqrt{K}\ln\left(\frac{|(K-1)[A]-(\sqrt{K}-1)[A_0]|}{|(K-1)[A]+(\sqrt{K}+1)[A_0]|}\right)}{2[A_0]}$ of the W dialkyl complex *vs.* time.

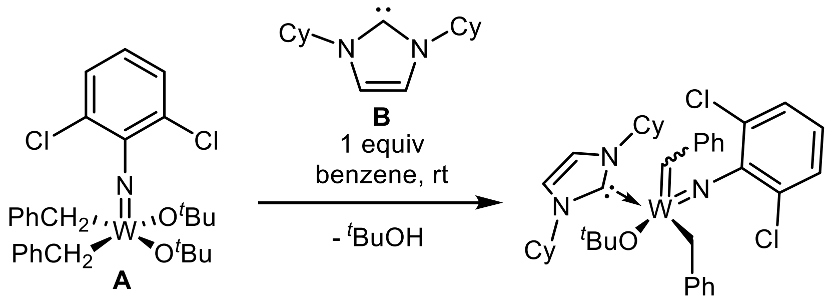

Scheme S4. Formation of **5**.

Initial concentrations:

[A] = 37.2 mM

[B] = 39.3 mM

[cyclooctane] = 34.9 mM

K = 292 (determined after 95 min)

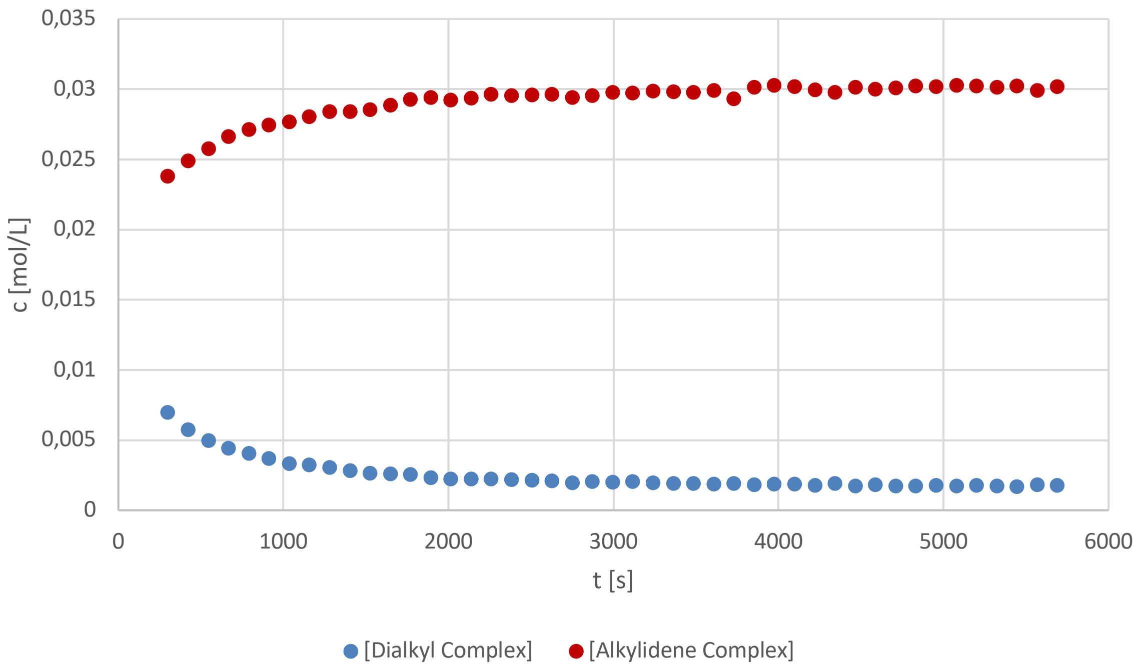

Figure S6. Formation of **5**; plot of concentrations of the W dialkyl and W alkylidene complex.

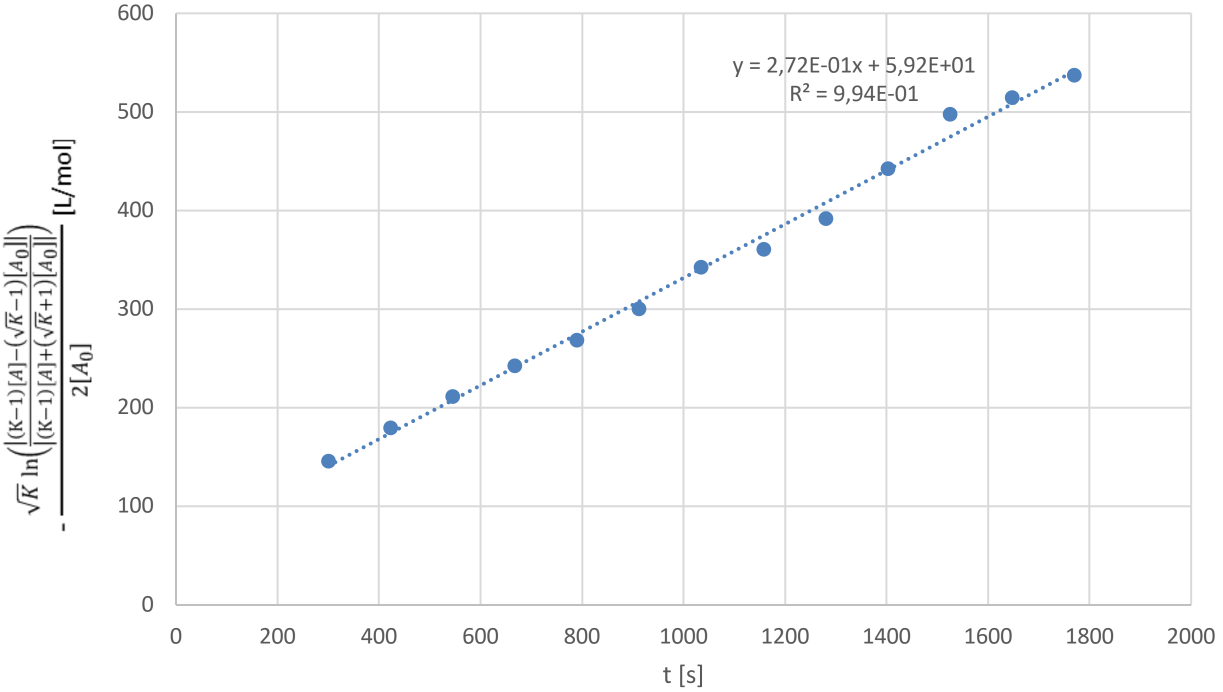

Figure S7. Formation of **5**; plot of $-\frac{\sqrt{K}\ln\left(\frac{|(K-1)[A]-(\sqrt{K}-1)[A_0]|}{|(K-1)[A]+(\sqrt{K}+1)[A_0]|}\right)}{2[A_0]}$ of the W dialkyl complex *vs.* time.

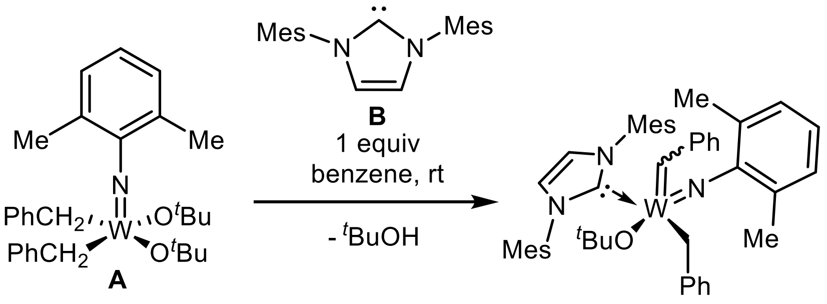

Scheme S5. Formation of **9**.

Initial concentrations:

[A] = 37.2 mM

[B] = 39.3 mM

[cyclooctane] = 36.7 mM

K = 0.116 (determined after 18 h)

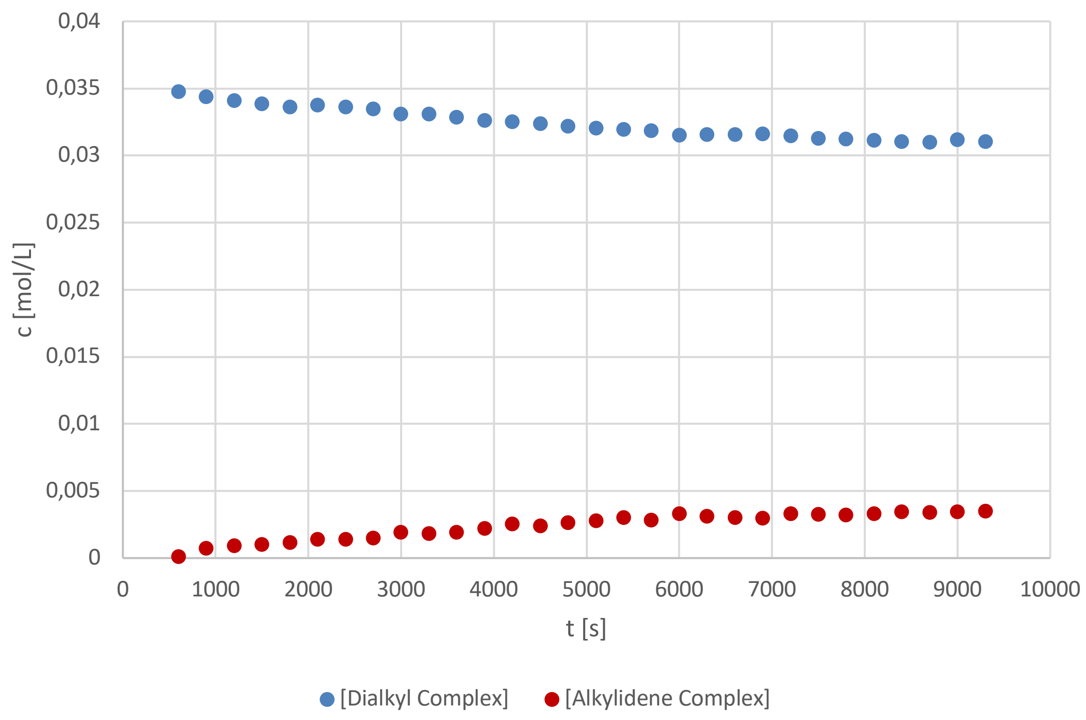

Figure S8. Formation of **9**; plot of concentrations of the W dialkyl and W alkylidene complex.

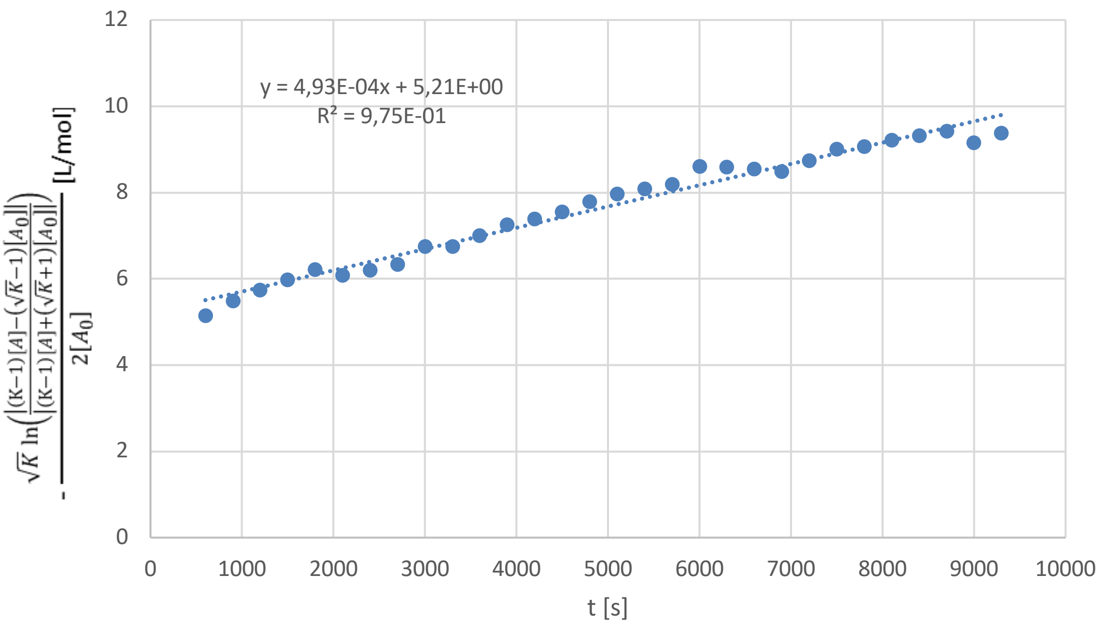

Figure S9. Formation of **9**; plot of $-\frac{\sqrt{K}\ln\left(\frac{|(K-1)[A]-(\sqrt{K}-1)[A_0]|}{|(K-1)[A]+(\sqrt{K}+1)[A_0]|}\right)}{2[A_0]}$ of the W dialkyl complex *vs*. time.

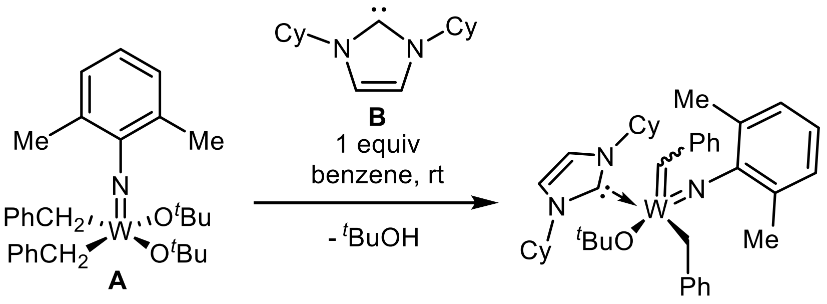

Scheme S6. Formation of **7**.

Initial concentrations:

[A] = 37.2 mM

[B] = 39.3 mM

[cyclooctane] = 34.9 mM

K = 10.5 (determined after 5 h)

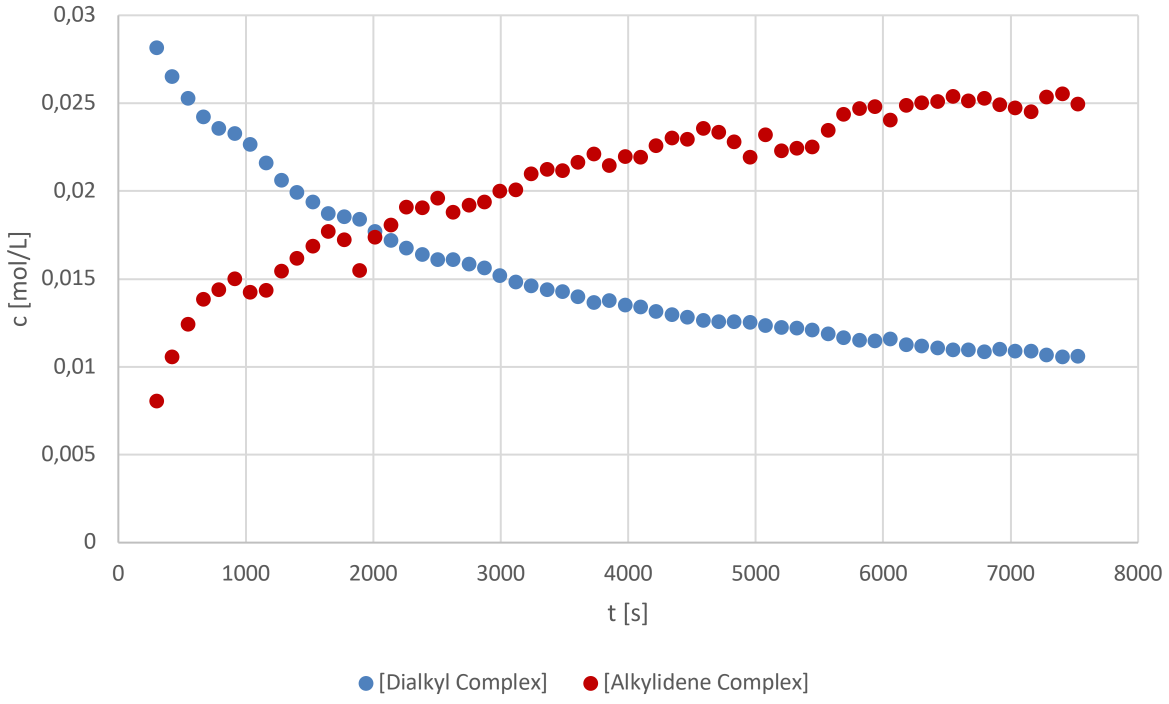

Figure S10. Formation of **7**; Integrated equilibrium equation is applied to the concentration of dialkyl complex.

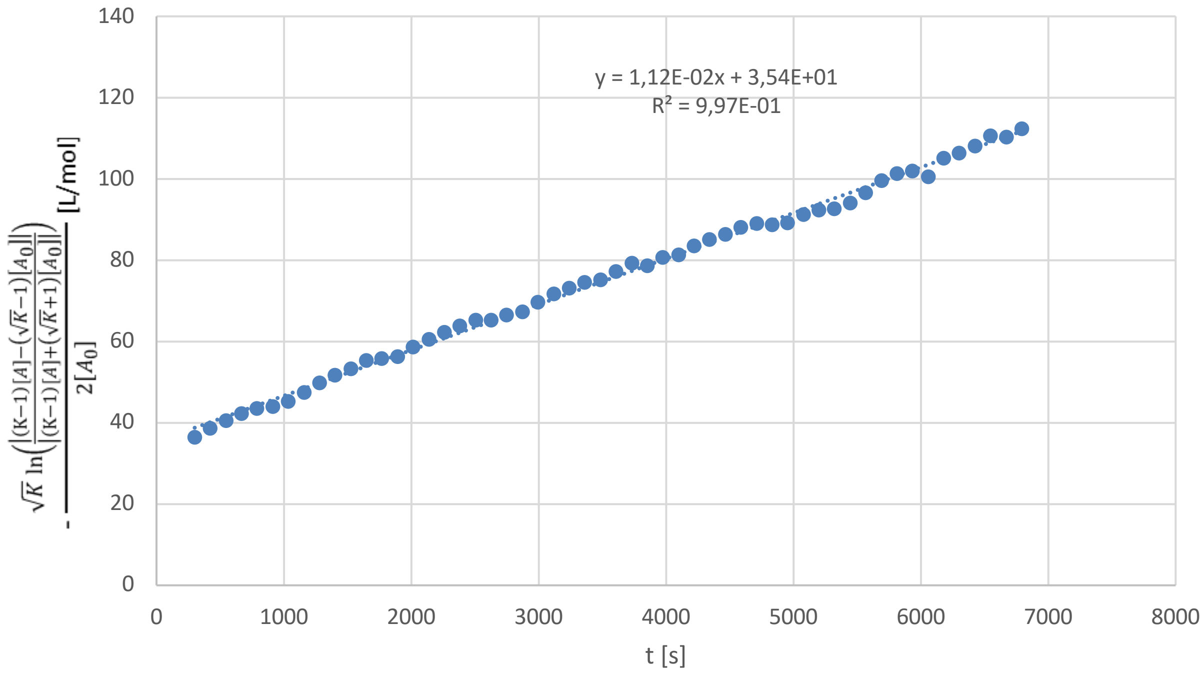

Figure S11. Formation of **7**; plot of $-\frac{\sqrt{K}\ln\left(\frac{|(K-1)[A]-(\sqrt{K}-1)[A_0]|}{|(K-1)[A]+(\sqrt{K}+1)[A_0]|}\right)}{2[A_0]}$ of the W dialkyl complex *vs.* time.

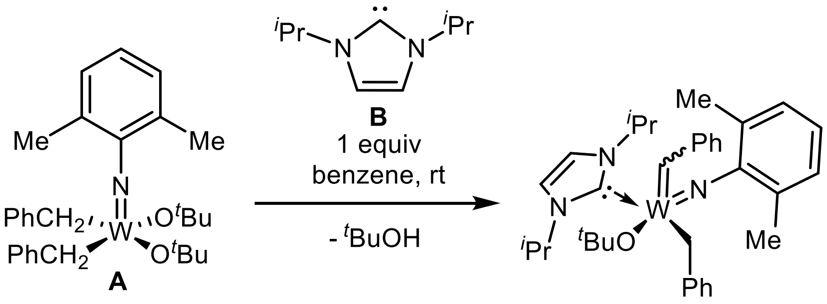

Scheme S7. Formation of **8**.

Initial concentrations:

[A] = 37.2 mM

[B] = 39.4 mM

[cyclooctane] = 33.9 mM

K = 1.52 (determined after 125 min)

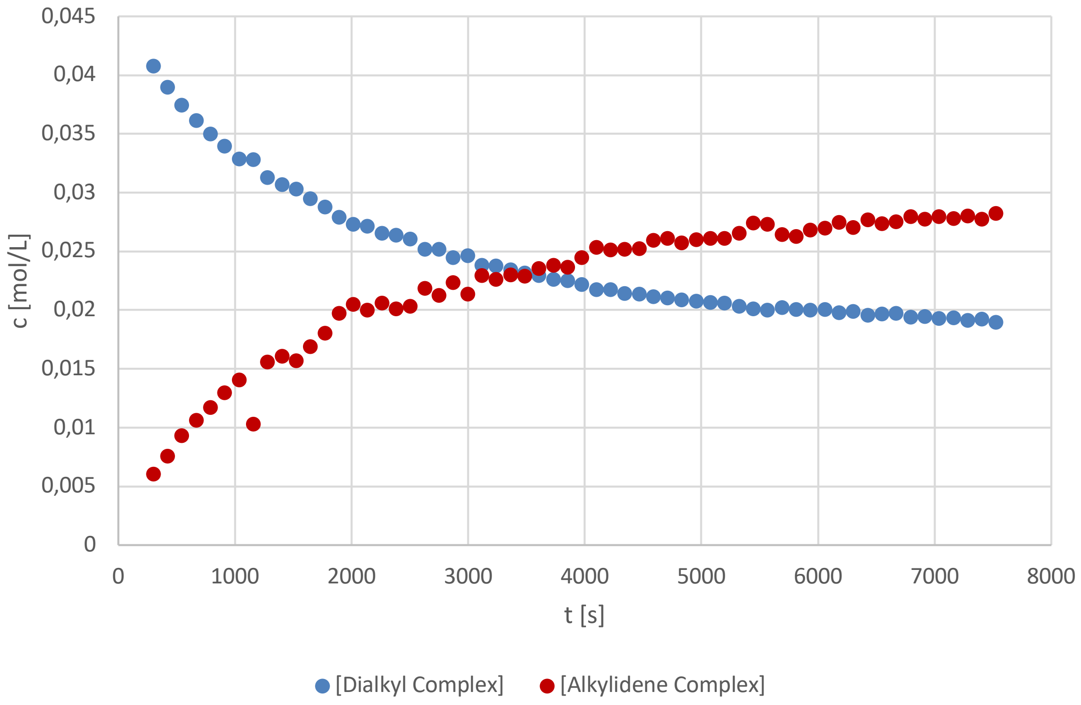

Figure S12. Formation of **8**; Integrated equilibrium equation is applied to concentration of dialkyl complex.

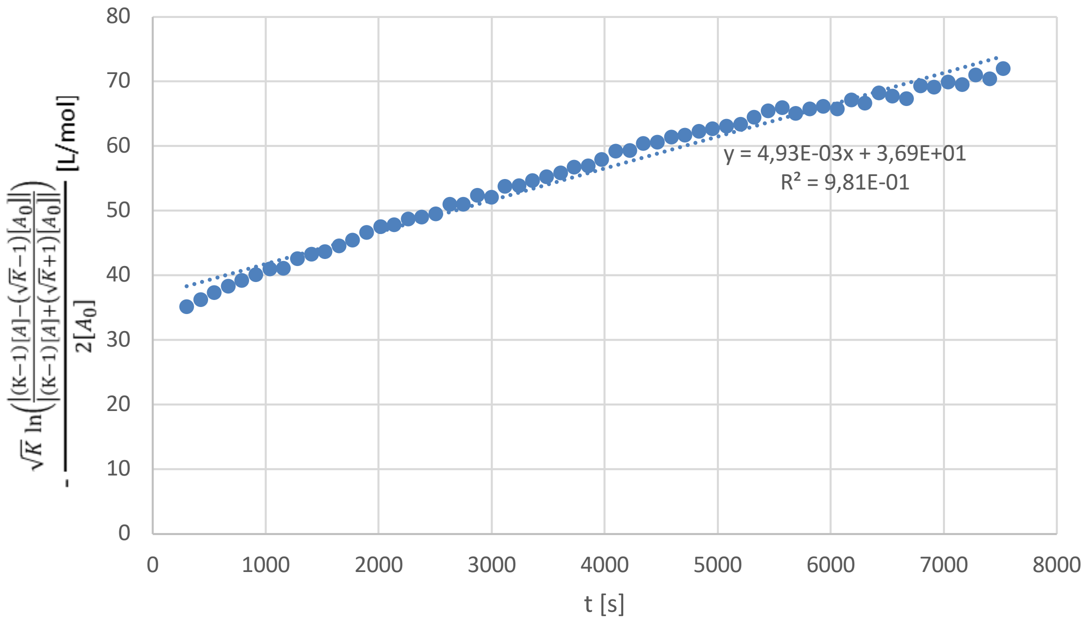

Figure S13. Formation of **8**; plot of $-\frac{\sqrt{K}\ln\left(\frac{|(K-1)[A]-(\sqrt{K}-1)[A_0]|}{|(K-1)[A]+(\sqrt{K}+1)[A_0]|}\right)}{2[A_0]}$ of the W dialkyl complex *vs.* time.

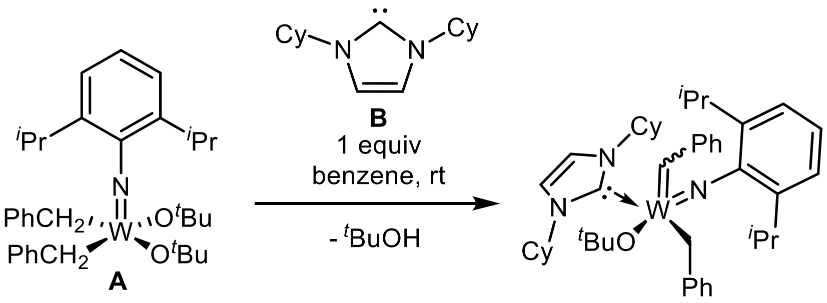

Scheme S8. Formation of **10**.

Initial concentrations:

[A] = 37.2 mM

[B] = 39.3 mM

[cyclooctane] = 34.9 mM

K = 0.286 (determined after 21 h)

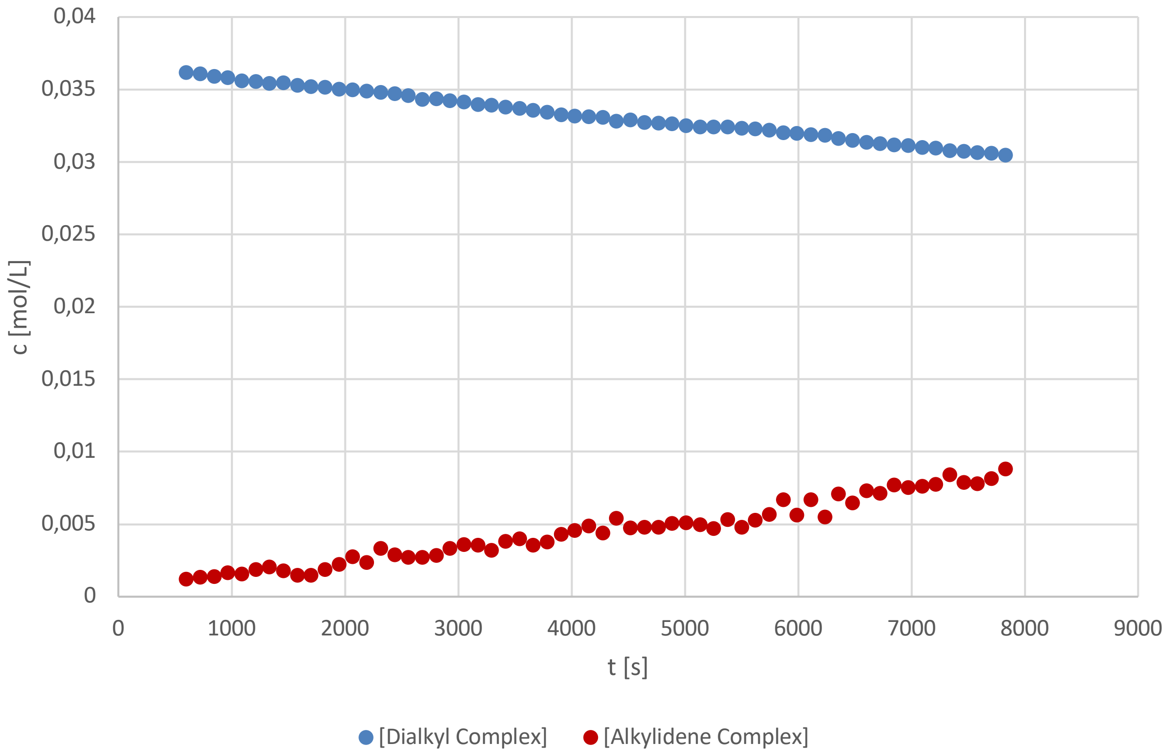

Figure S14. Formation of **10**; plot of concentrations of the W dialkyl and W alkylidene complex.

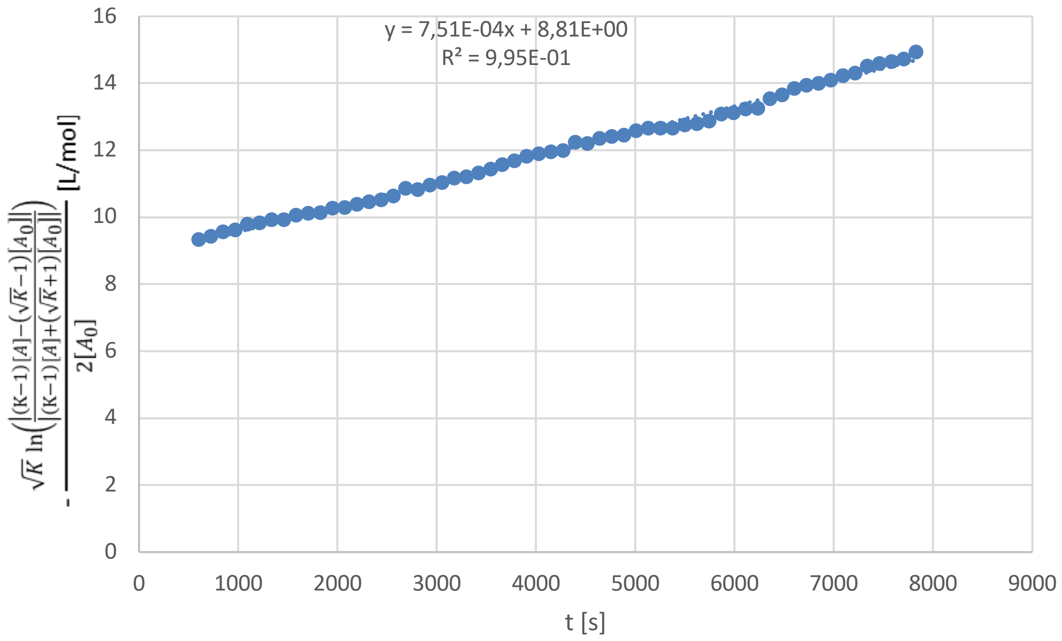

Figure S15. Formation of **10**; plot of $-\frac{\sqrt{K}\ln\left(\frac{|(K-1)[A]-(\sqrt{K}-1)[A_0]|}{|(K-1)[A]+(\sqrt{K}+1)[A_0]|}\right)}{2[A_0]}$ of the W dialkyl complex *vs*. time.

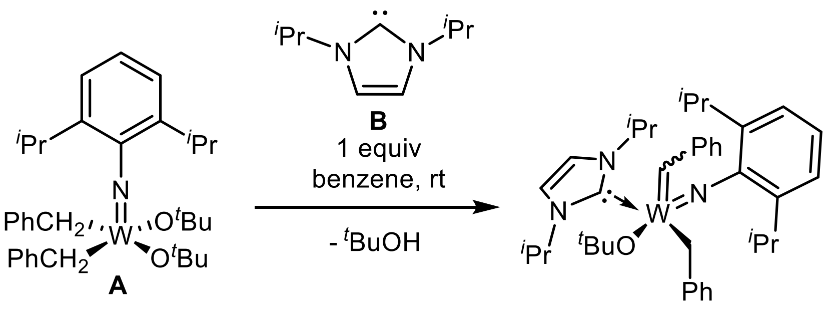

Scheme S9. Formation of **11**.

Initial concentrations:

[A] = 37.2 mM

[B] = 39.4 mM

[cyclooctane] = 33.9 mM

K = 0.0224 (determined after 23 h)

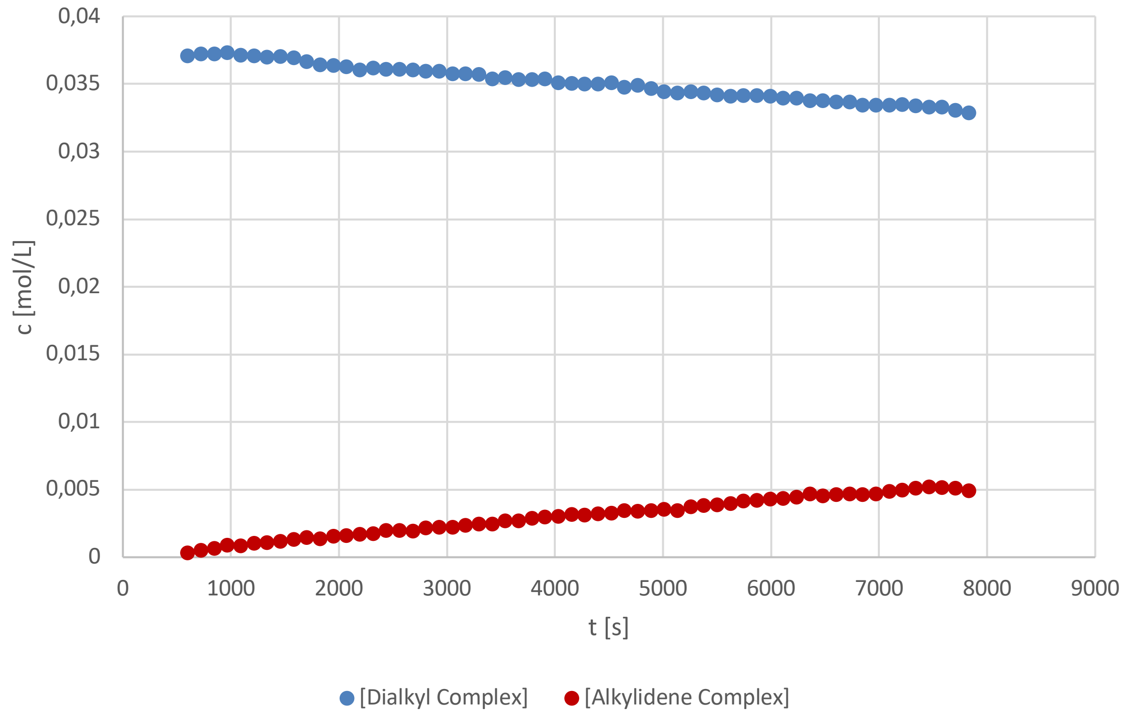

Figure S16. Formation of **11**; plot of concentrations of the W dialkyl and W alkylidene complex.

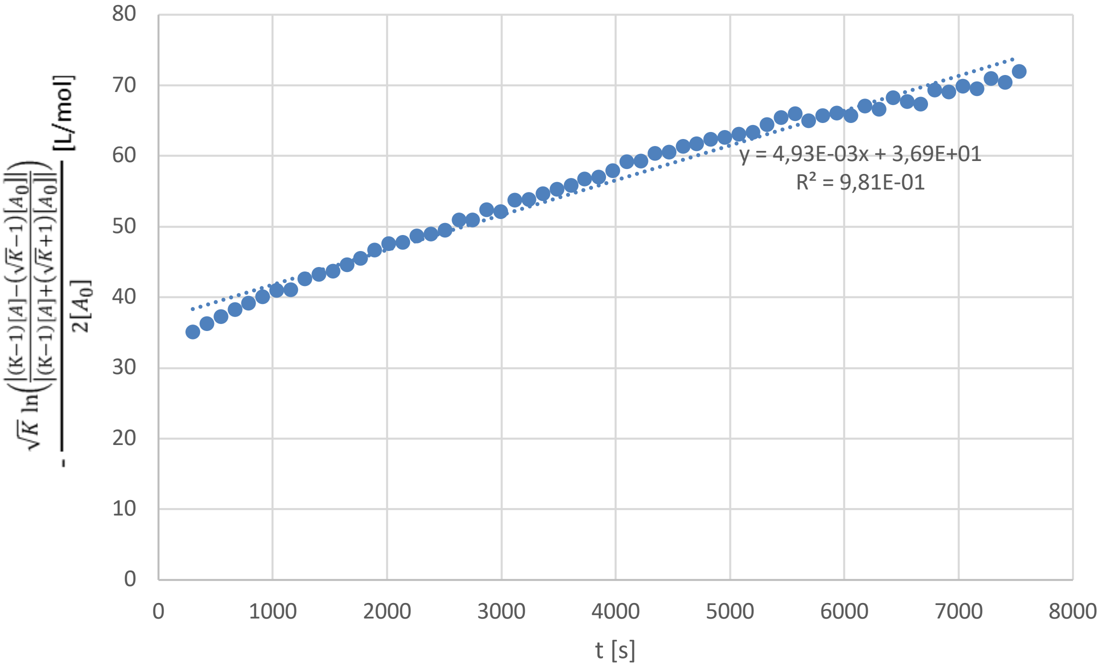

Figure S17. Formation of **11**; plot of $-\frac{\sqrt{K}\ln\left(\frac{|(K-1)[A]-(\sqrt{K}-1)[A_0]|}{|(K-1)[A]+(\sqrt{K}+1)[A_0]|}\right)}{2[A_0]}$ of the W dialkyl complex *vs*. time.

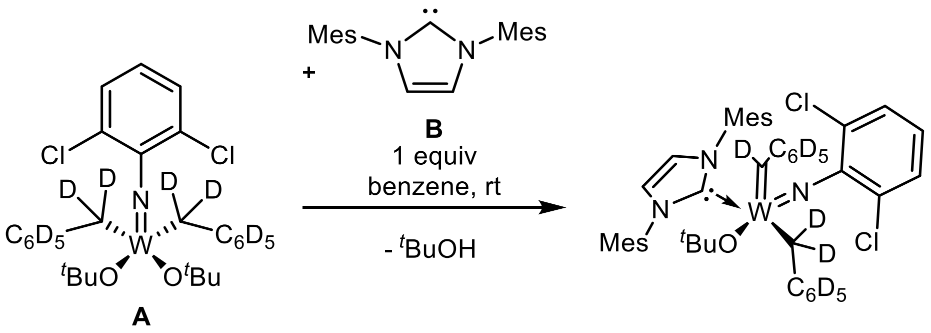

Scheme S10. Formation of **1d**.

Initial concentrations:

[A] = 37.2

[B] = 39.3 mM

[cyclooctane] = 36.7 mM

K = 0.444 (determined after 17 h)

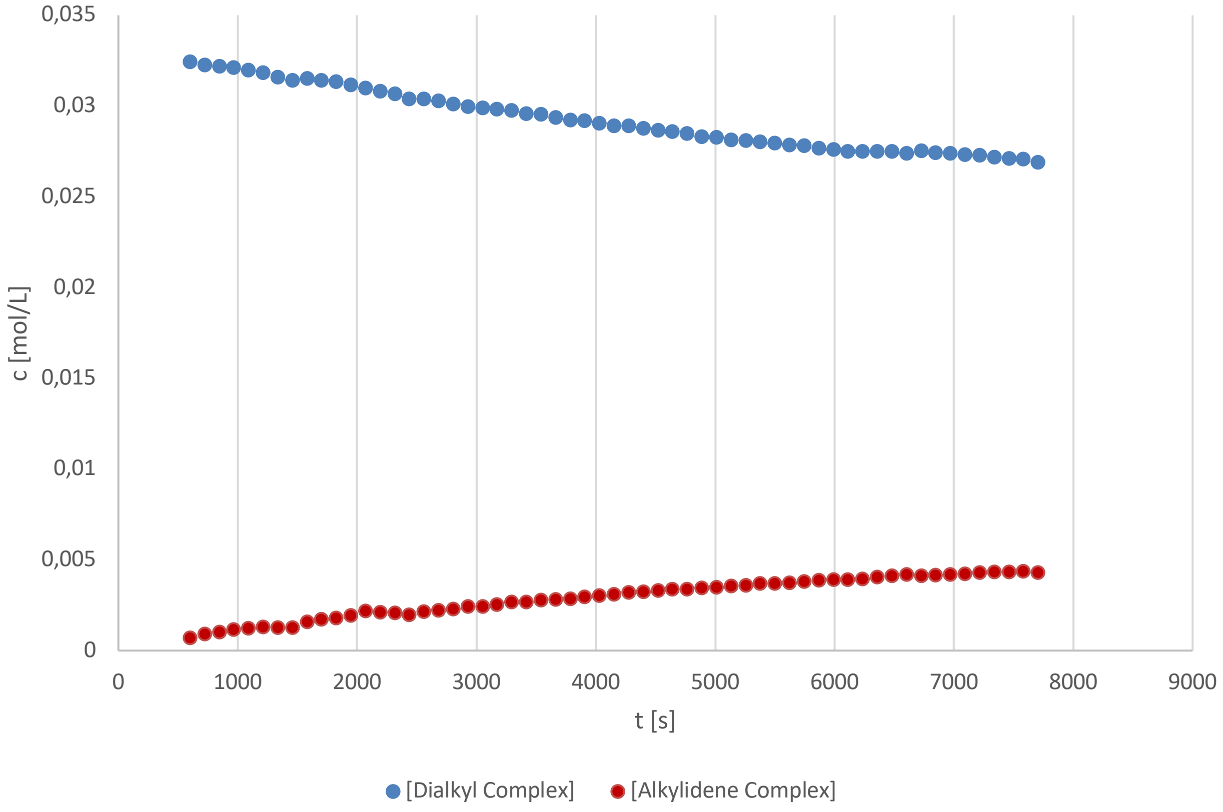

Figure S18. Formation of **1d**; plot of concentrations of the W dialkyl and W alkylidene complex.

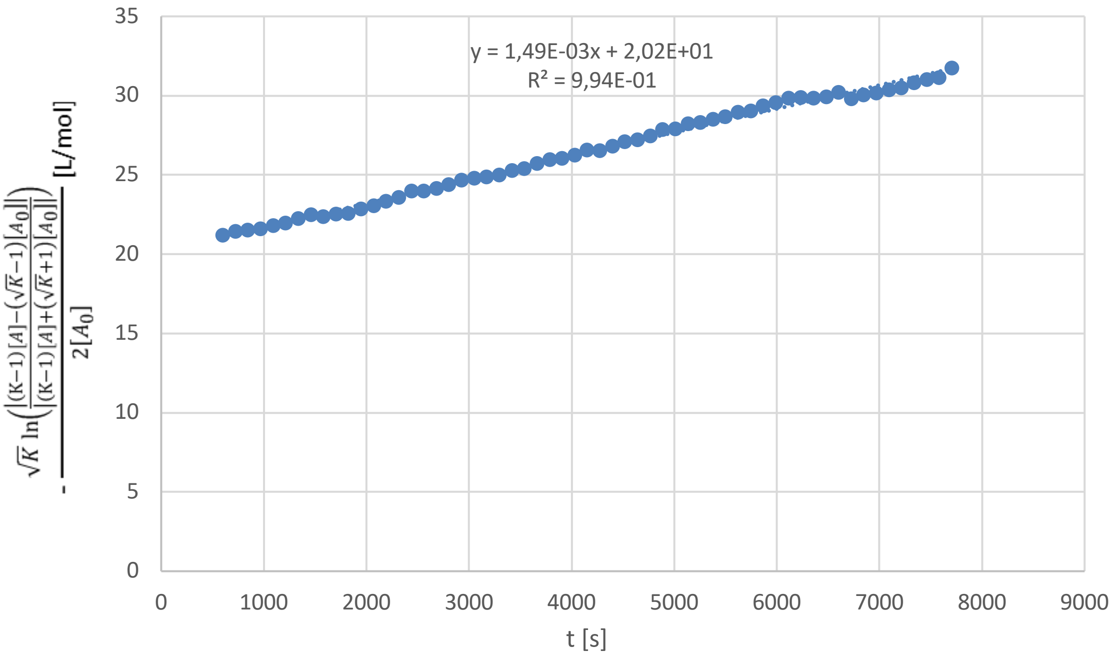

Figure S19. Formation of **1d**; plot of $-\frac{\sqrt{K}\ln\left(\frac{|(K-1)[A]-(\sqrt{K}-1)[A_0]|}{|(K-1)[A]+(\sqrt{K}+1)[A_0]|}\right)}{2[A_0]}$ of the W dialkyl complex *vs*. time.

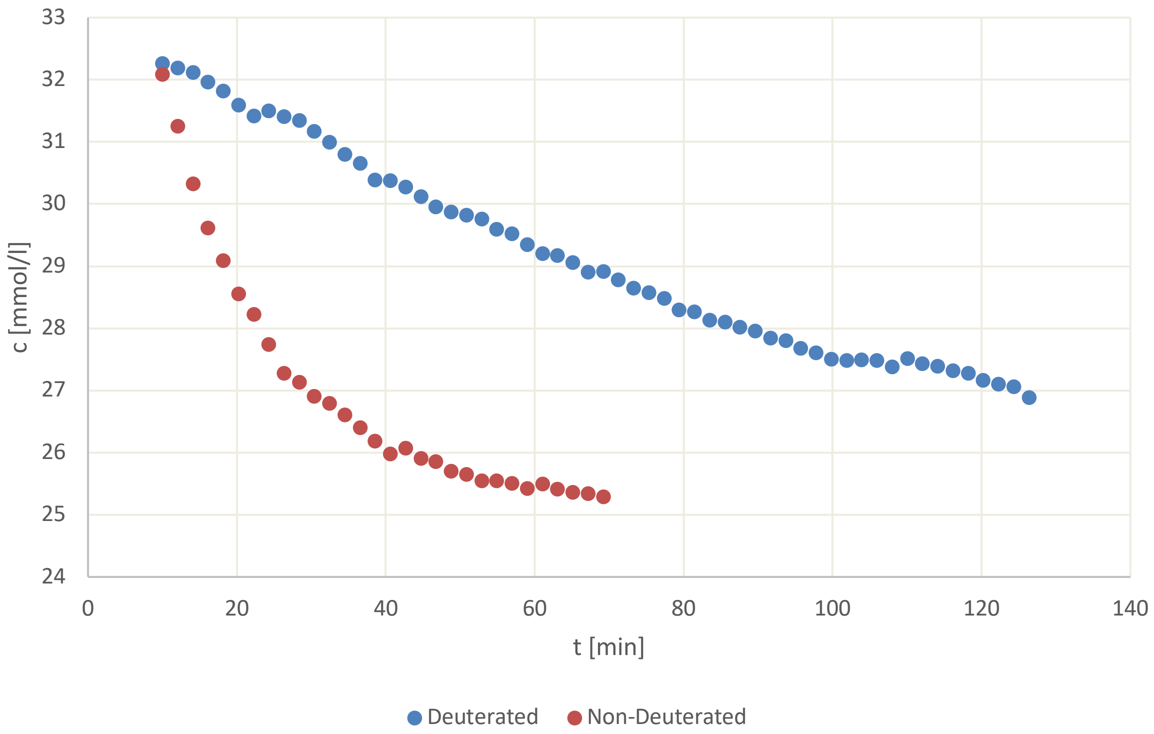

Figure S20. Comparison of decreasing concentrations of the deuterated complex (W(*N*-2,6-dichlorophenyl)($CD_2C_6D_5$)$_2$(O^{t}Bu)2) vs. the non-deuterated complex (W(*N*-2,6-dichlorophenyl)($CH_2C_6H_5$)$_2$(O^{t}Bu)$_2$) in the reaction with IMes, initial concentrations as aforementioned, internal standard: cyclooctane.

NMR Spectra

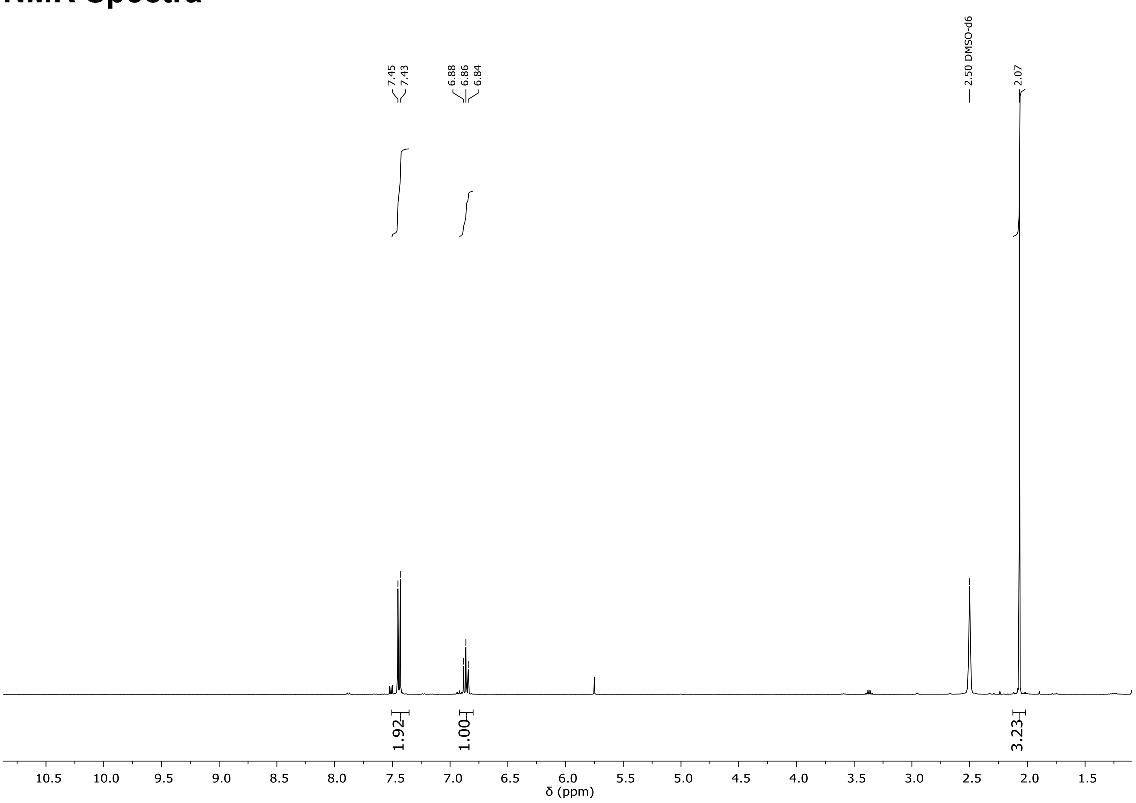

Figure S21. ^{1}H NMR spectrum of W(N-2,6-dichlorophenyl)Cl_4(MeCN) in DMSO-d6.

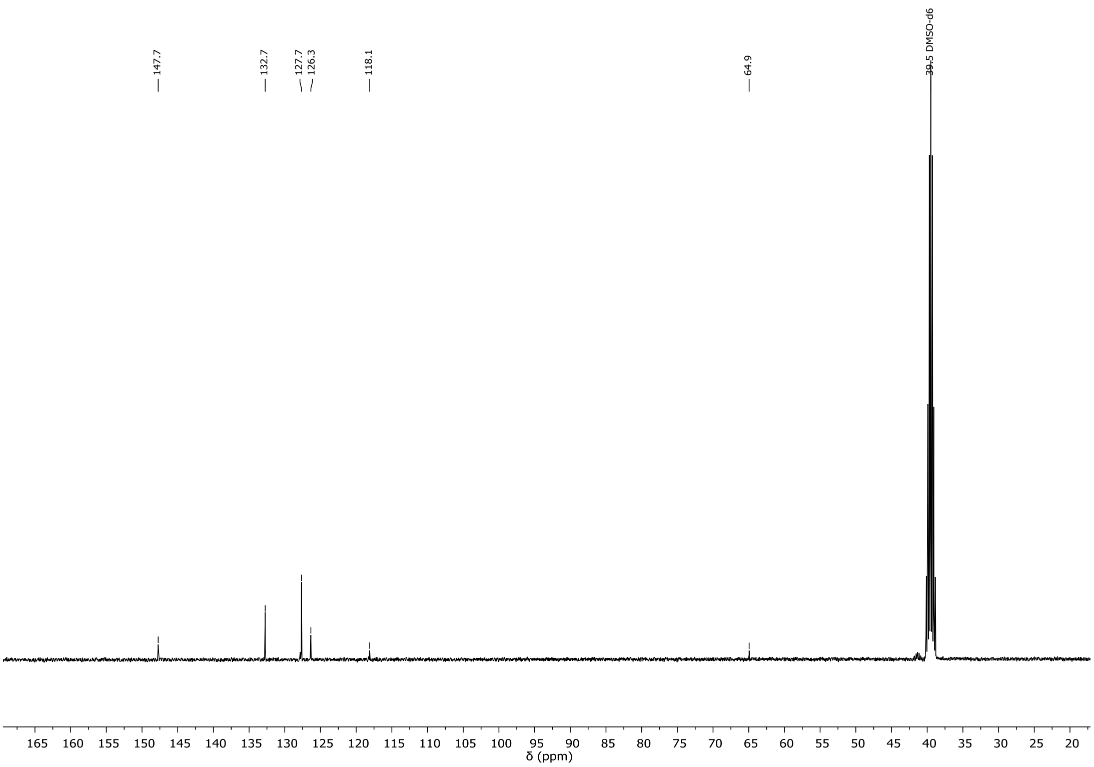

Figure S22. ^{13}C NMR spectrum of W(N-2,6-dichlorophenyl)Cl_4(MeCN) in DMSO-d6.

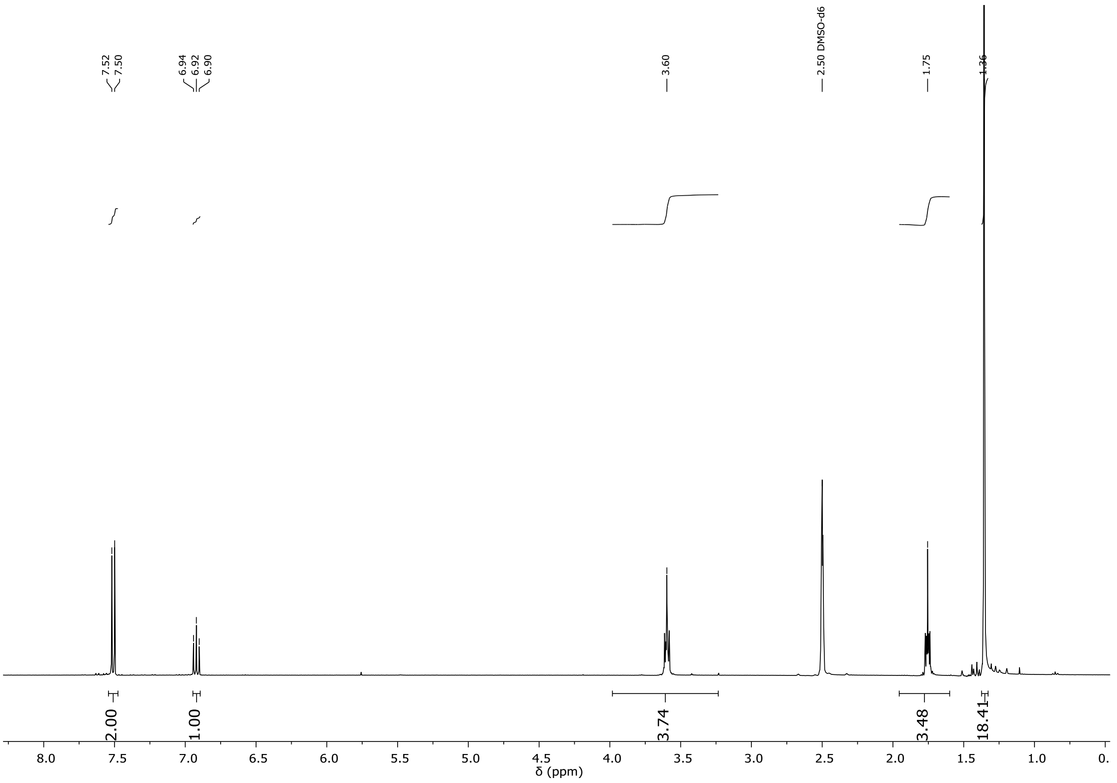

Figure S23. ^{1}H NMR spectrum of W(*N*-2,6-dichlorophenyl)Cl_2(O^{t}Bu)$_2$(THF) in DMSO-d6.

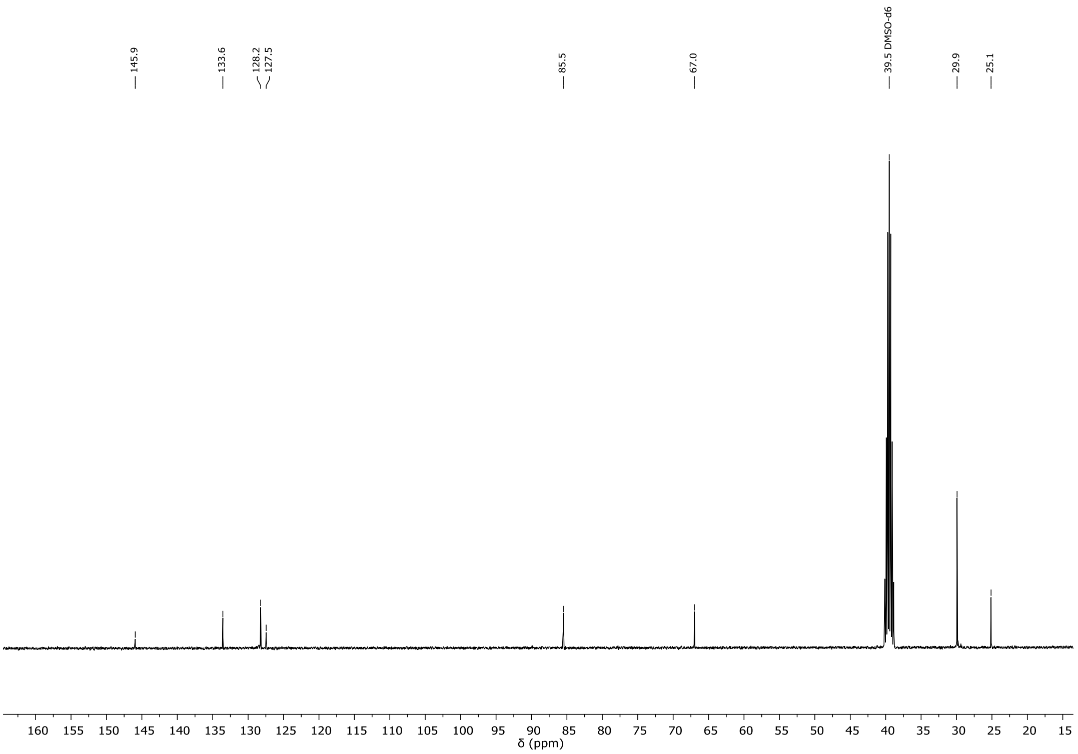

Figure S24. ^{13}C NMR spectrum of W(*N*-2,6-dichlorophenyl)Cl_2(O^{t}Bu)$_2$(THF) in DMSO-d6.

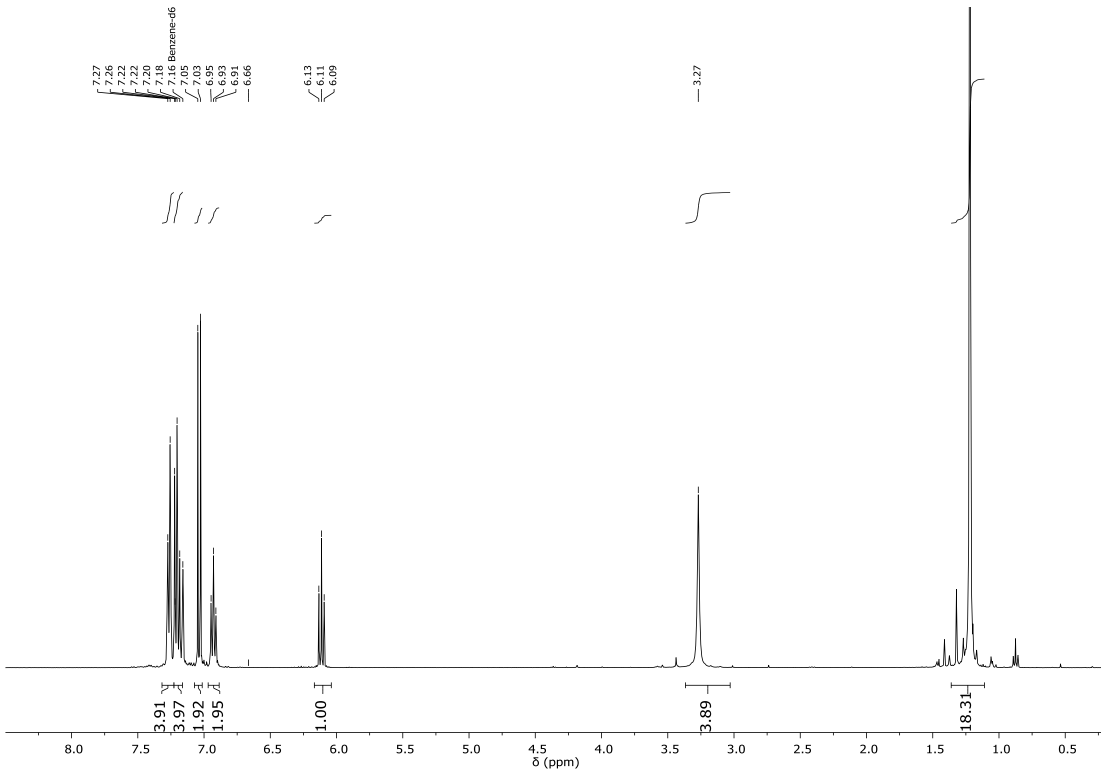

Figure S25. ^{1}H NMR spectrum of W(*N*-2,6-dichlorophenyl)(CH_2Ph)$_2$(O^{t}Bu)$_2$ (**1**) in C_6D_6.

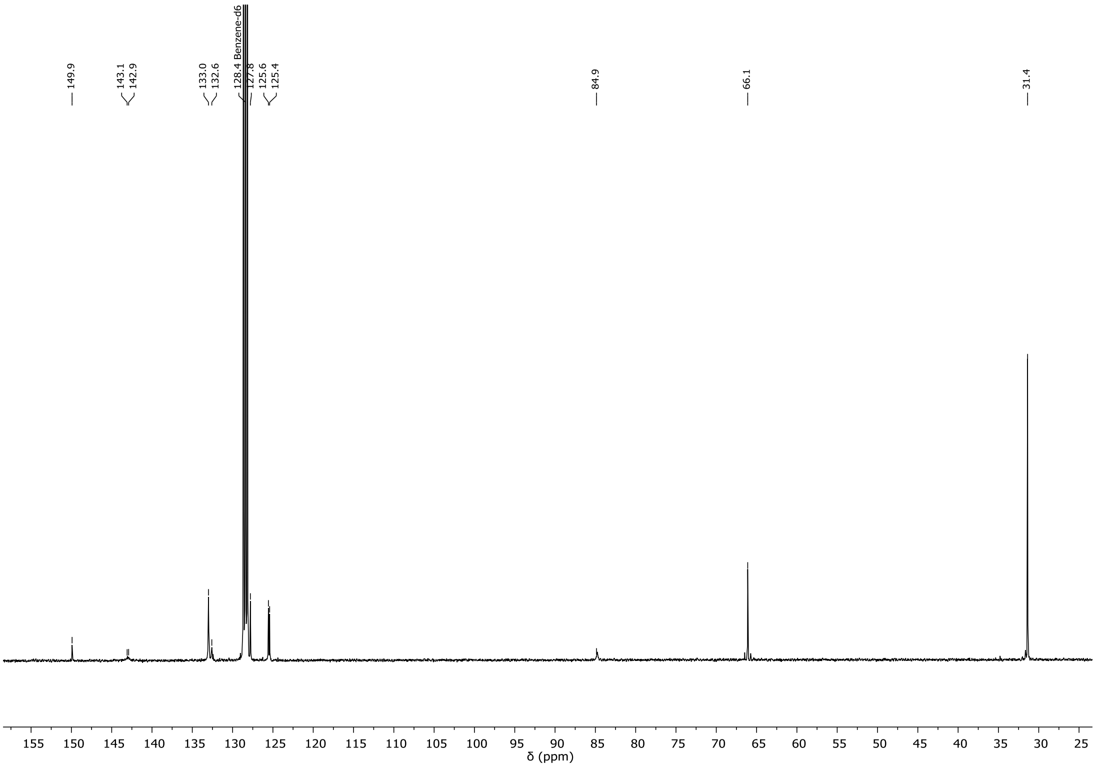

Figure S26. ^{13}C NMR spectrum of W(*N*-2,6-dichlorophenyl)(CH_2Ph)$_2$(O^{t}Bu)$_2$ (**1**) in C_6D_6.

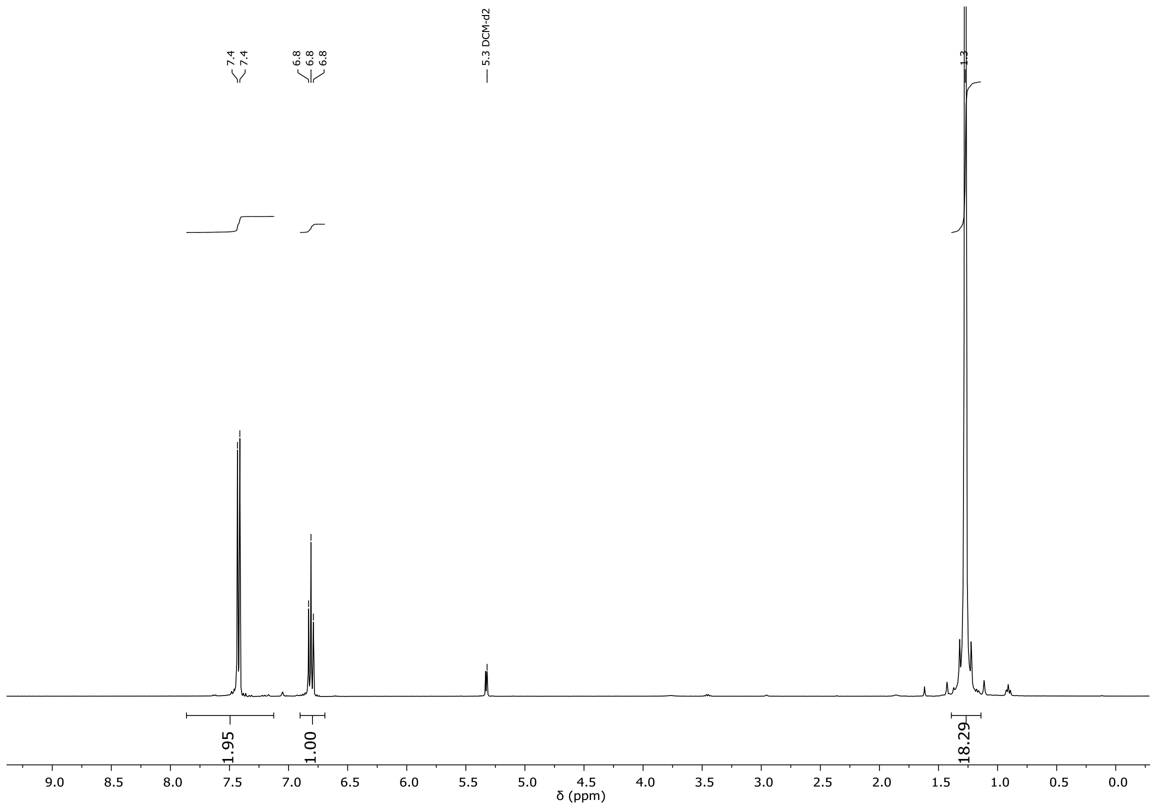

Figure S27. ^{1}H NMR spectrum of W(*N*-2,6-dichlorophenyl)($CD_2C_6D_5$)$_2$(O^{t}Bu)$_2$ (**1d**) in CD_2Cl_2.

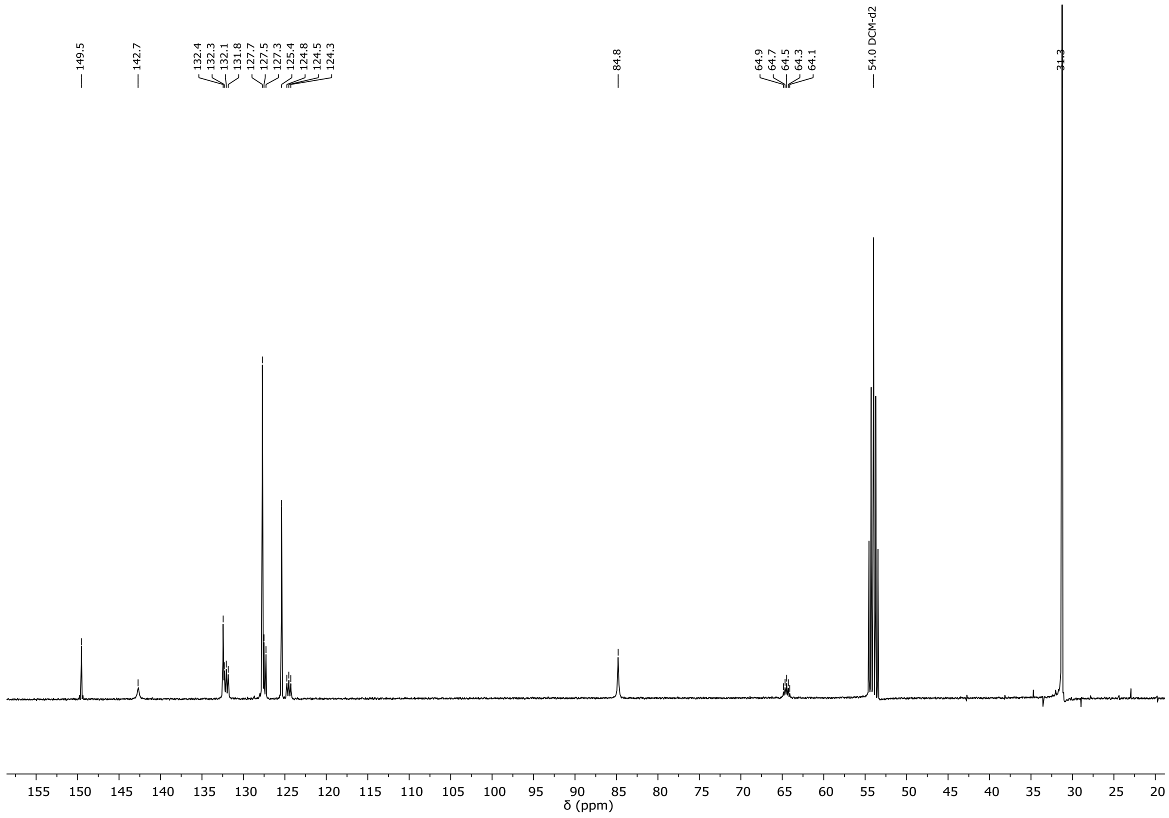

Figure S28. ^{13}C NMR spectrum of W(*N*-2,6-dichlorophenyl)($CD_2C_6D_5$)$_2$(O^{t}Bu)$_2$ (**1d**) in CD_2Cl_2.

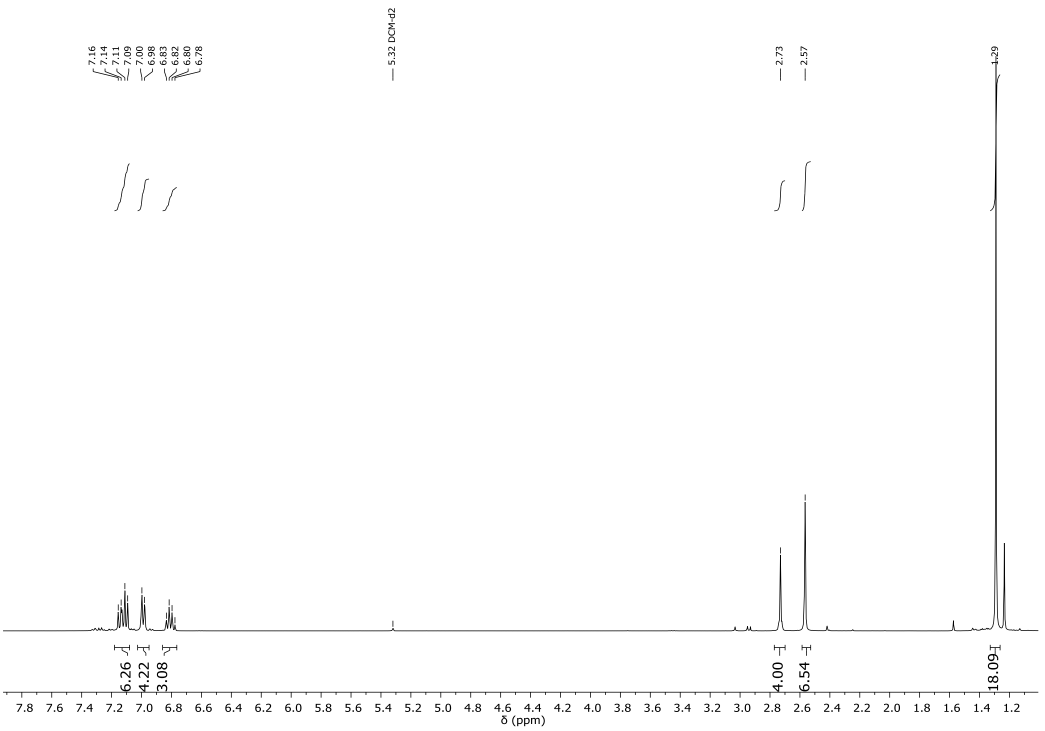

Figure S29. ^{1}H NMR spectrum of W(*N*-2,6-dimethylphenyl)($CH_2Ph)_2(O^tBu)_2$ (**3**) in CD_2Cl_2.

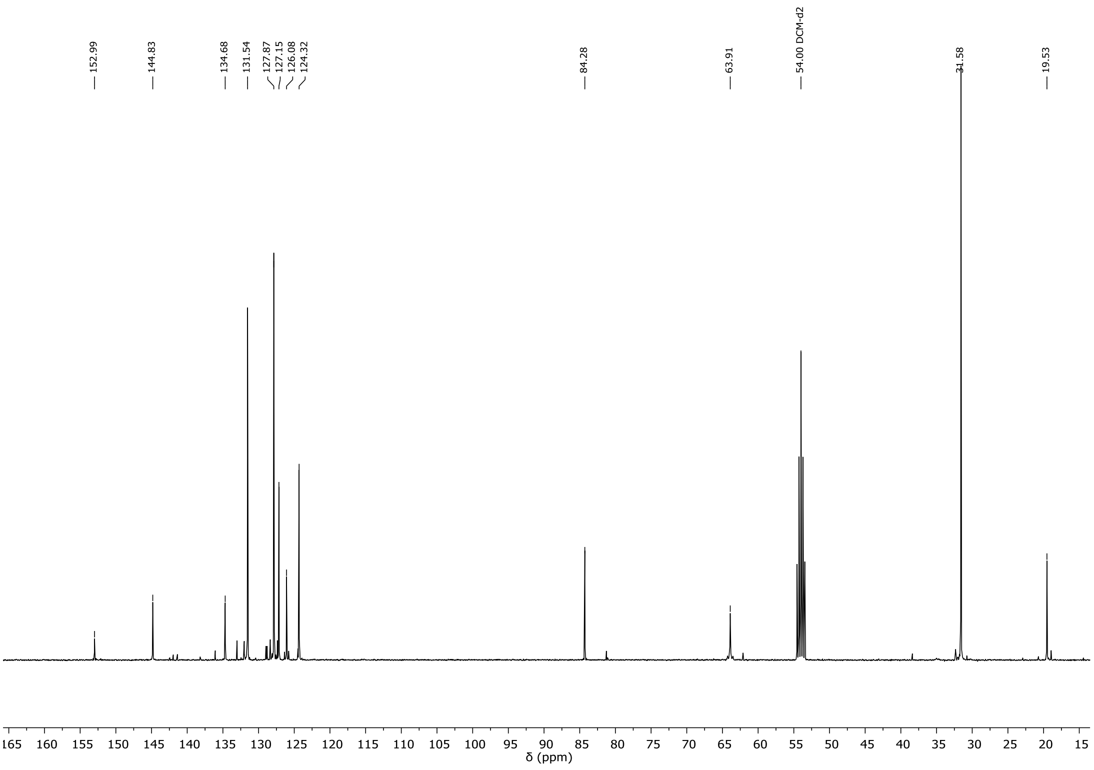

Figure S30. ^{13}C NMR spectrum of W(*N*-2,6-dimethylphenyl)($CH_2Ph)_2(O^tBu)_2$ (**3**) in CD_2Cl_2.

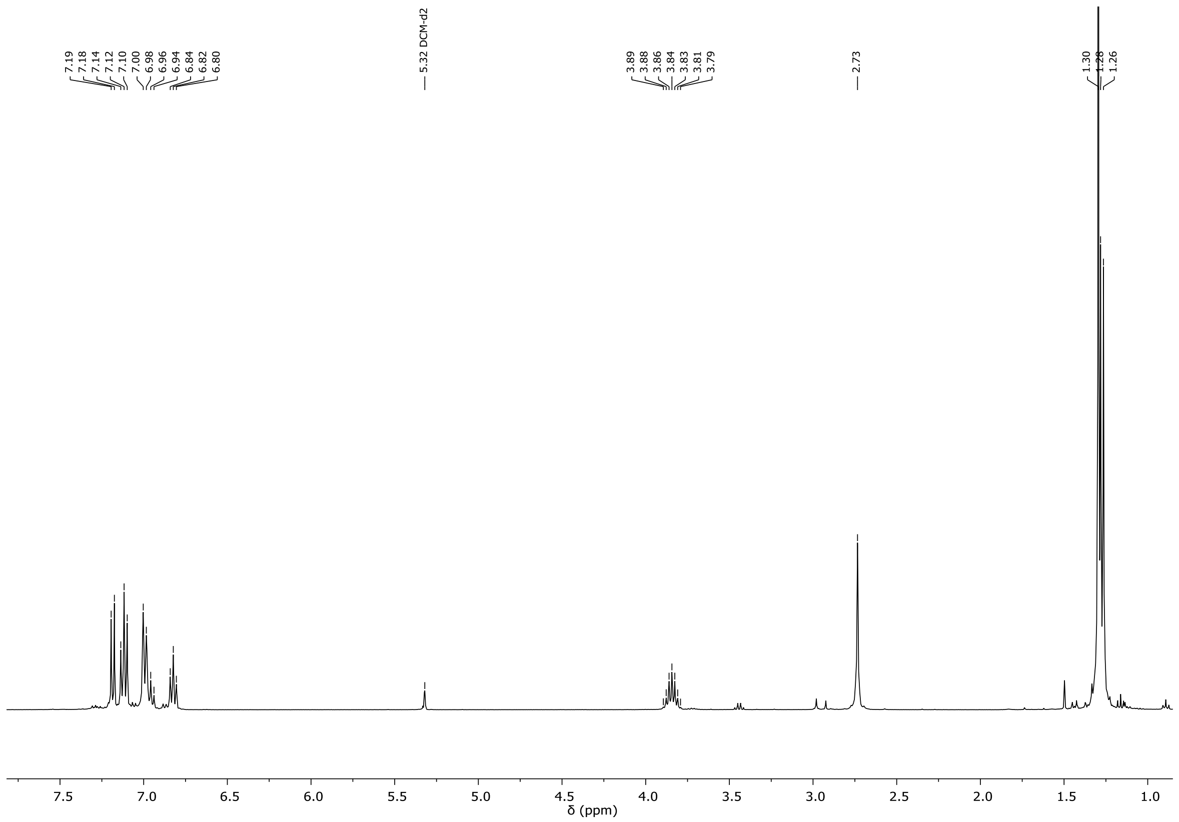

Figure S31. ^{1}H NMR spectrum of W(*N*-2,6-diisopropylphenyl)$(CH_2Ph)_2(O^tBu)_2$ (**4**) in CD_2Cl_2.

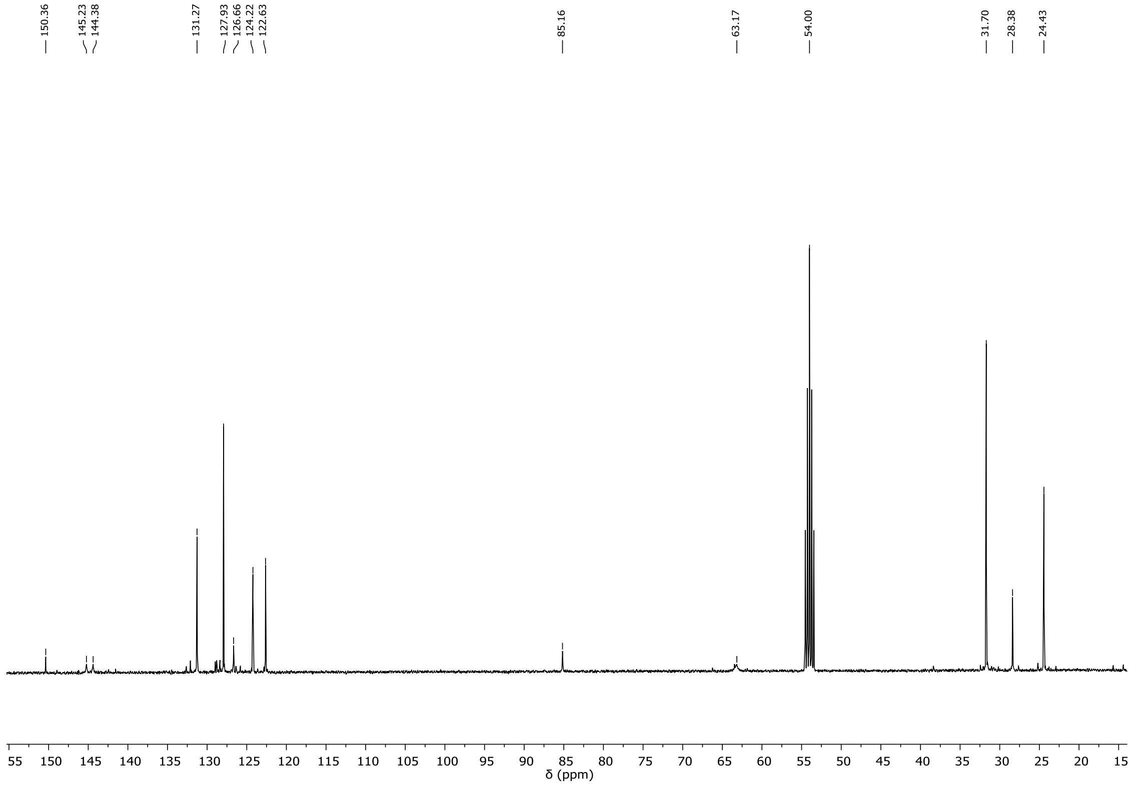

Figure S32. ^{13}C NMR spectrum of W(*N*-2,6-diisopropylphenyl)$(CH_2Ph)_2(O^tBu)_2$ (**4**) in CD_2Cl_2.

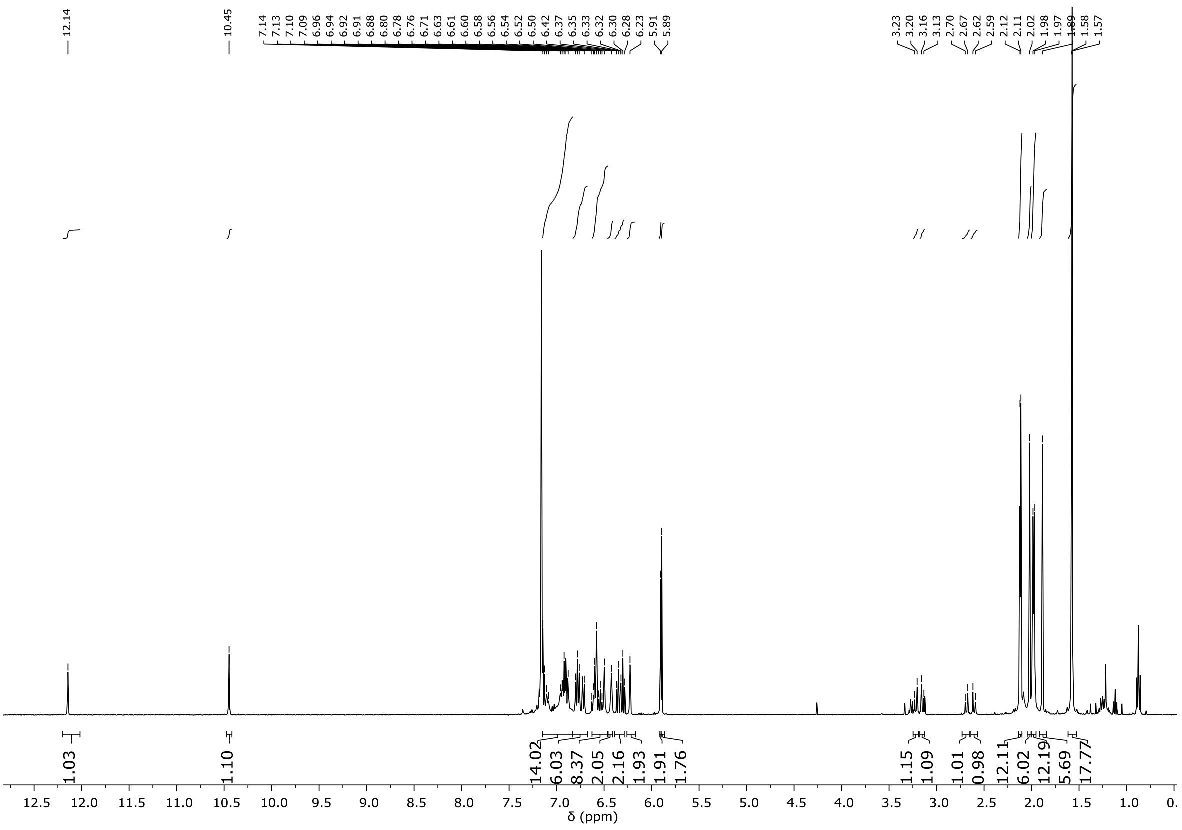

Figure S33. ^{1}H NMR spectrum of W(*N*-2,6-dichlorophenyl)(CHPh)(1,3-dimesitylimidazol-2-ylidene)(CH_2Ph)(O^tBu) (**2**) in C_6D_6.

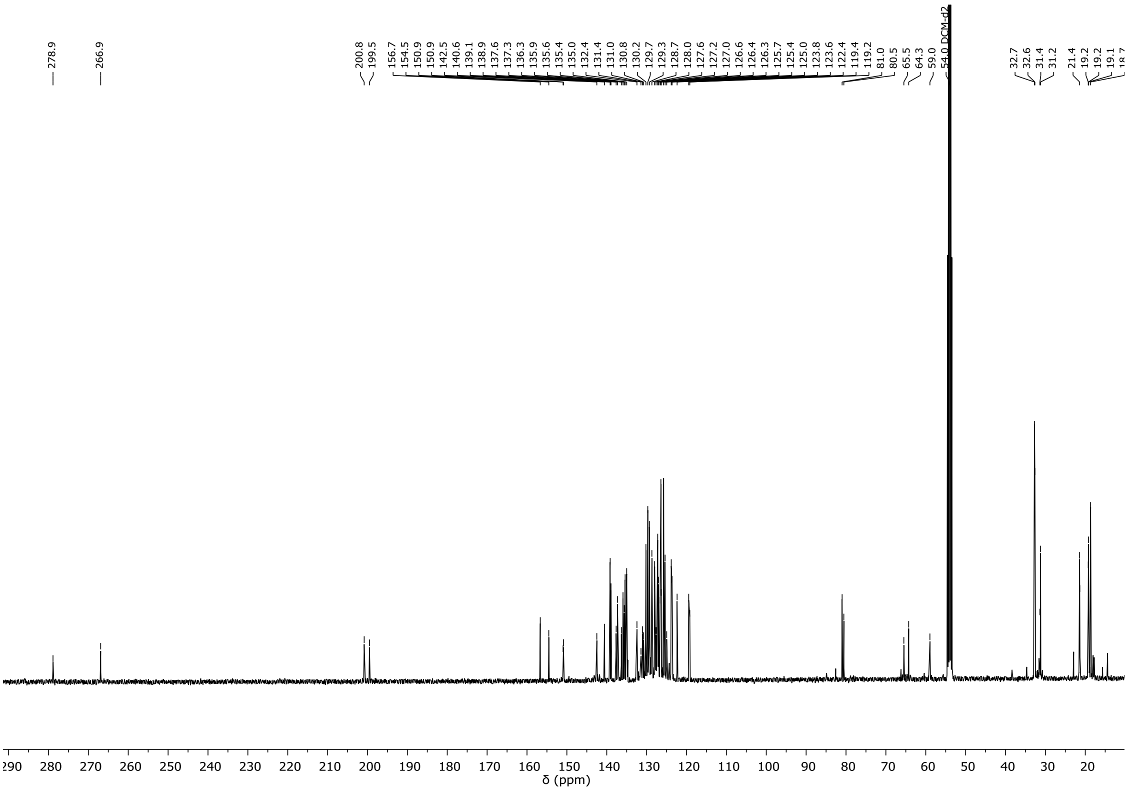

Figure S34. ^{13}C NMR spectrum of W(*N*-2,6-dichlorophenyl)(CHPh)(1,3-dimesitylimidazol-2-ylidene)(CH_2Ph)(O^tBu) (**2**) in CD_2Cl_2.

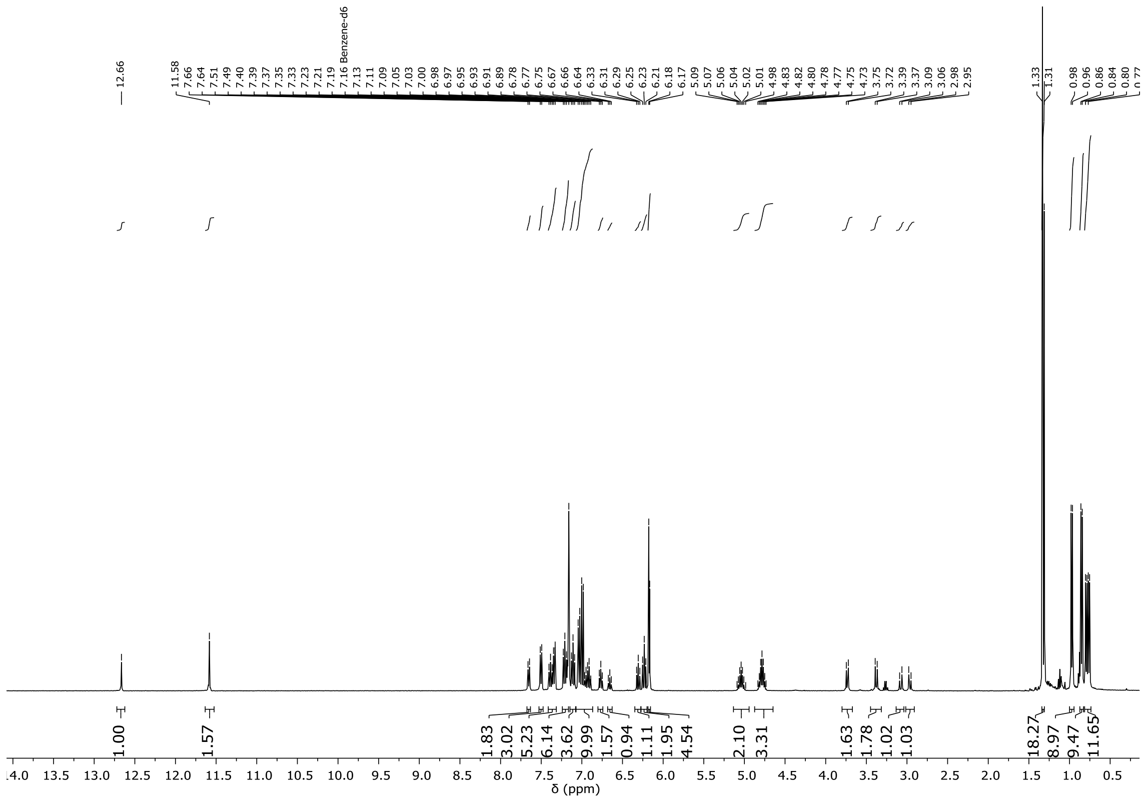

Figure S35. ^{1}H NMR spectrum of W(*N*-2,6-dichlorophenyl)(CHPh)(1,3-diisopropylimidazol-2-ylidene)(CH_2Ph)(O^{t}Bu) (**6**) in C_6D_6.

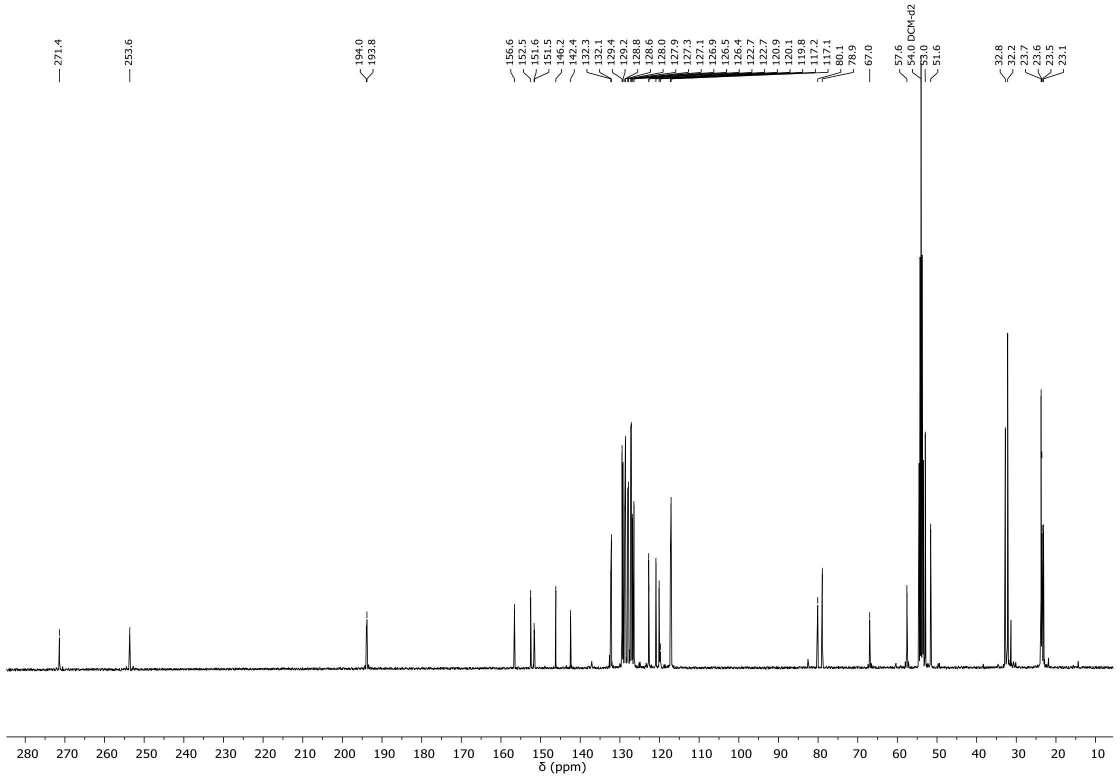

Figure S36. ^{13}C NMR spectrum of W(*N*-2,6-dichlorophenyl)(CHPh)(CH_2Ph)(O^{t}Bu)(1,3-diisopropylimidazol-2-ylidene) (**6**) in CD_2Cl_2.

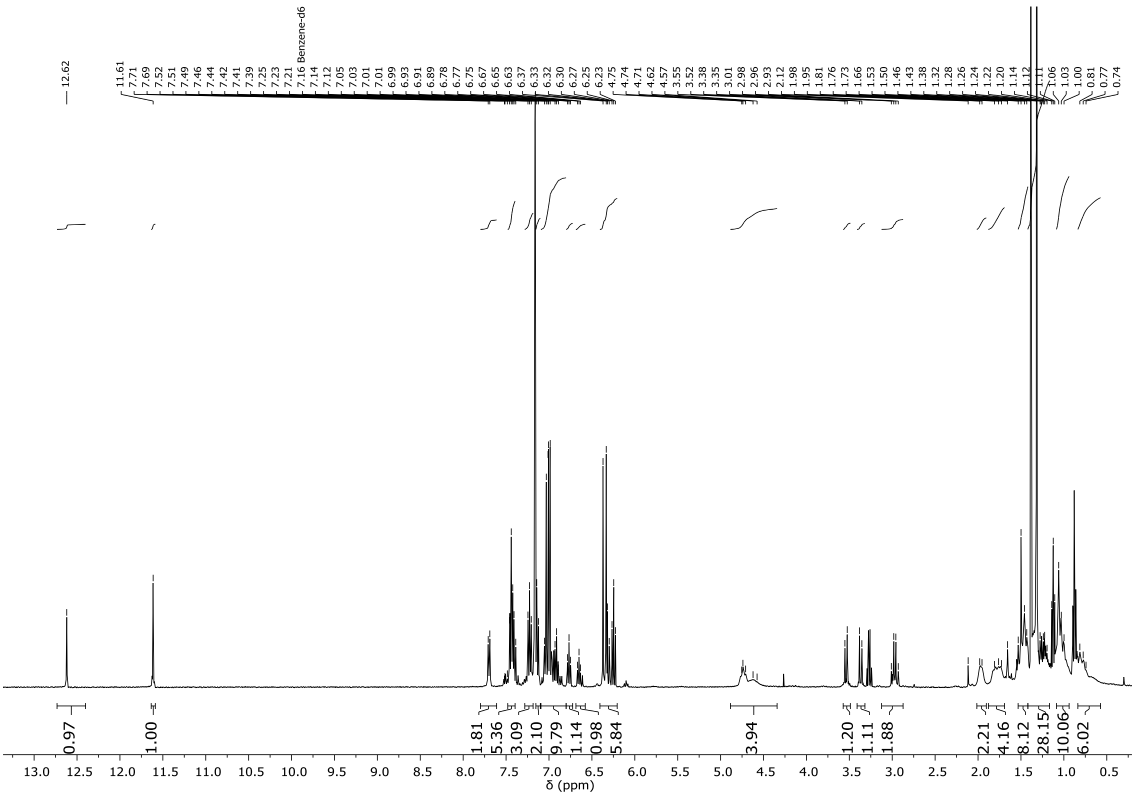

Figure S37. ^{1}H NMR spectrum of W(*N*-2,6-dichlorophenyl)(CHPh)(1,3-dicyclohexylimidazol-2-ylidene)(CH_2Ph)(O*^t^*Bu) (**5**) in C_6D_6.

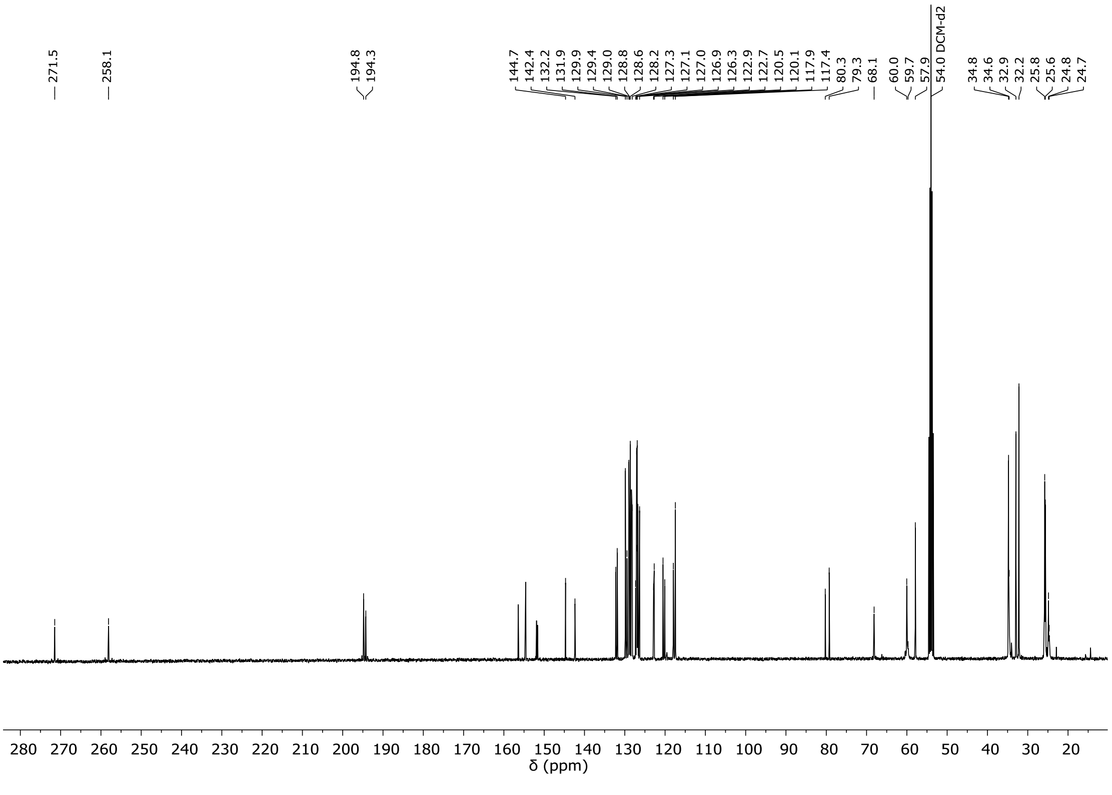

Figure S38. ^{13}C NMR spectrum of W(*N*-2,6-dichlorophenyl)(CHPh)(1,3-dicyclohexylimidazol-2-ylidene)(CH_2Ph)(O*^t^*Bu) (**5**) in CD_2Cl_2.

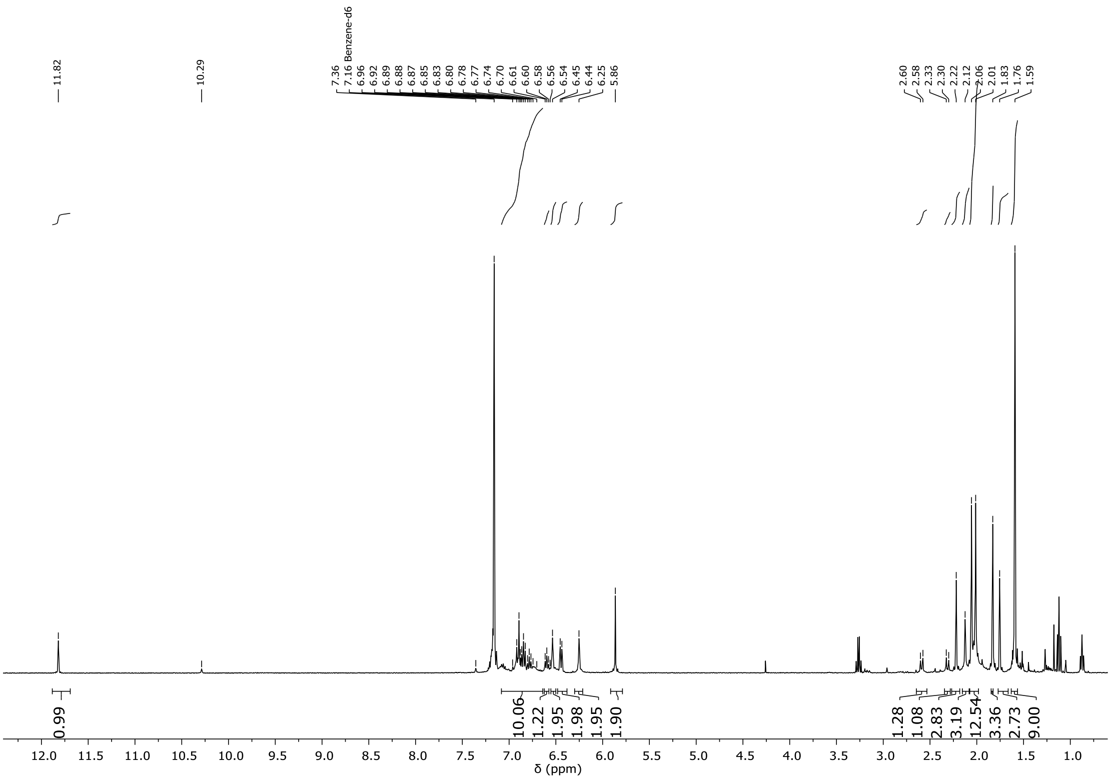

Figure S39. ^{1}H NMR spectrum of W(*N*-2,6-dimethylphenyl)(CHPh)(1,3-dimesitylimidazol-2-ylidene)(CH_2Ph)(O^tBu) (**9**) in C_6D_6 (minor isomer < 10%, not integrated for clarity).

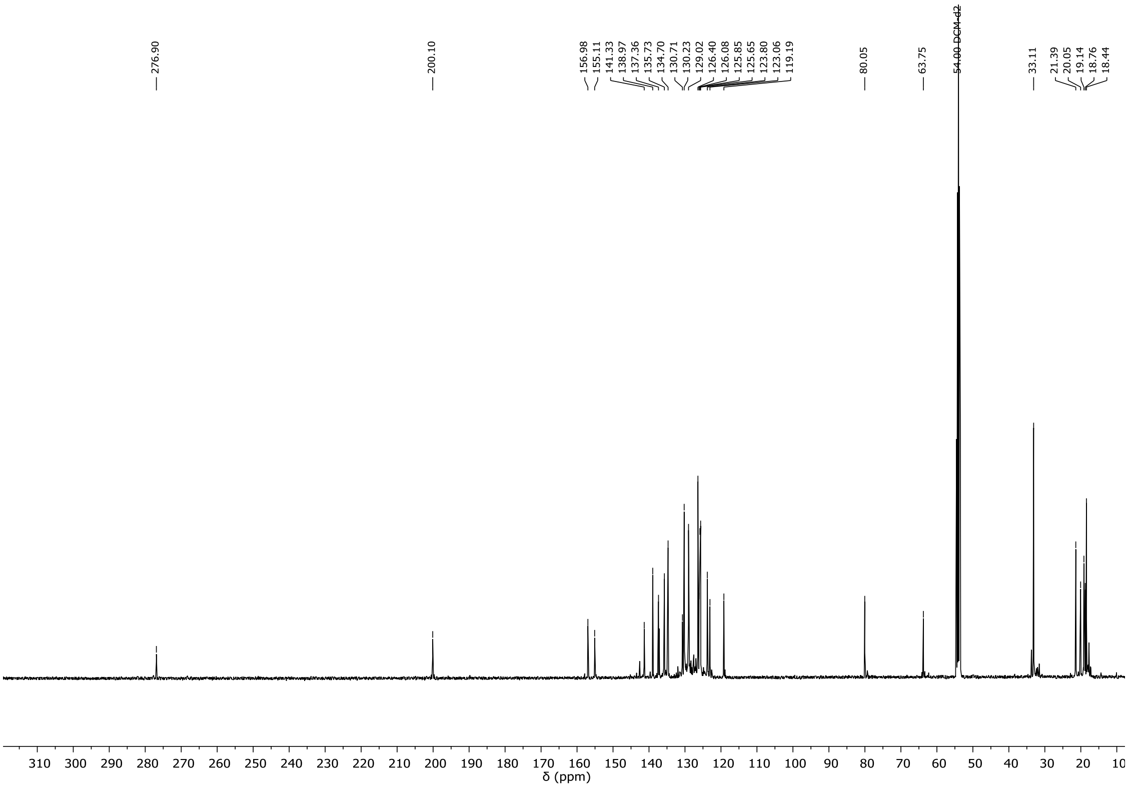

Figure S40. ^{13}C NMR spectrum of W(*N*-2,6-dimethylphenyl)(CHPh)(1,3-dimesitylimidazol-2-ylidene)(CH_2Ph)(O^tBu) (**9**) in CD_2Cl_2.

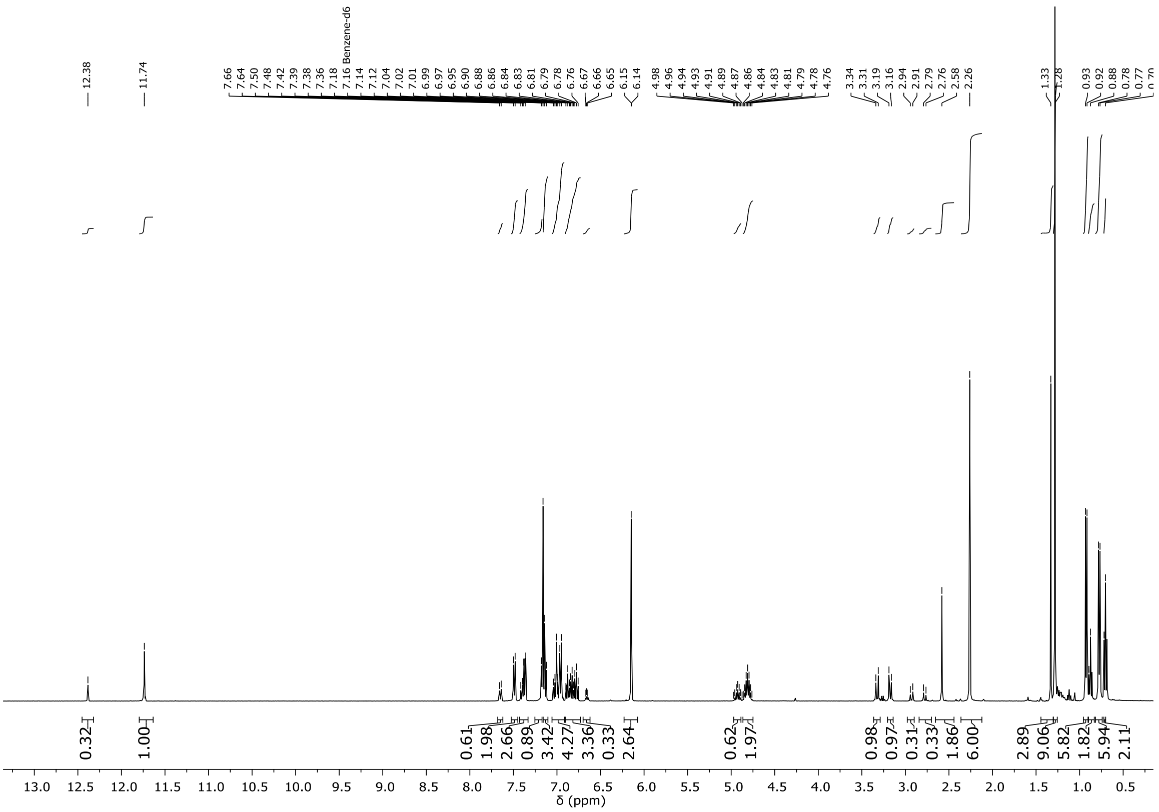

Figure S41. ^{1}H NMR spectrum of W(*N*-2,6-dimethylphenyl)(CHPh)(1,3-diisopropylimidazol-2-ylidene)(CH_2Ph)(O^tBu) (**8**) in C_6D_6.

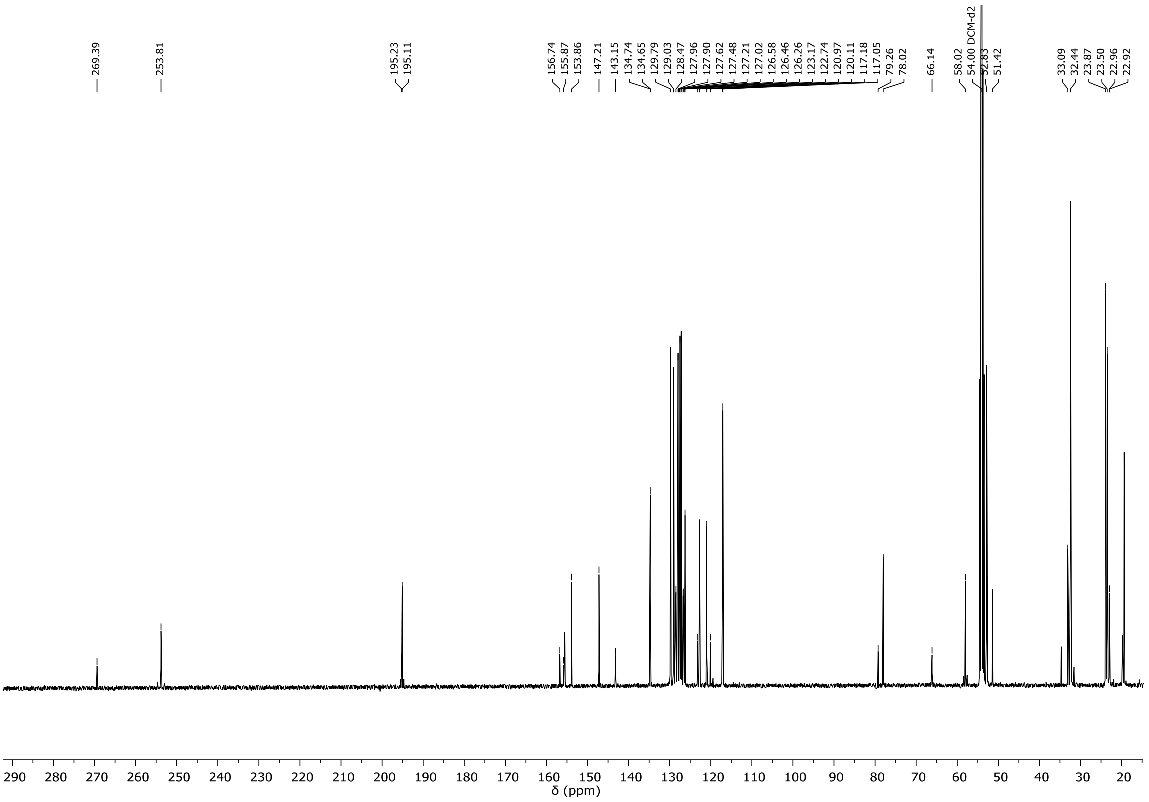

Figure S42. ^{13}C NMR spectrum of W(*N*-2,6-dimethylphenyl)(CHPh)(1,3-diisopropylimidazol-2-ylidene)(CH_2Ph)(O^tBu) (**8**) in CD_2Cl_2.

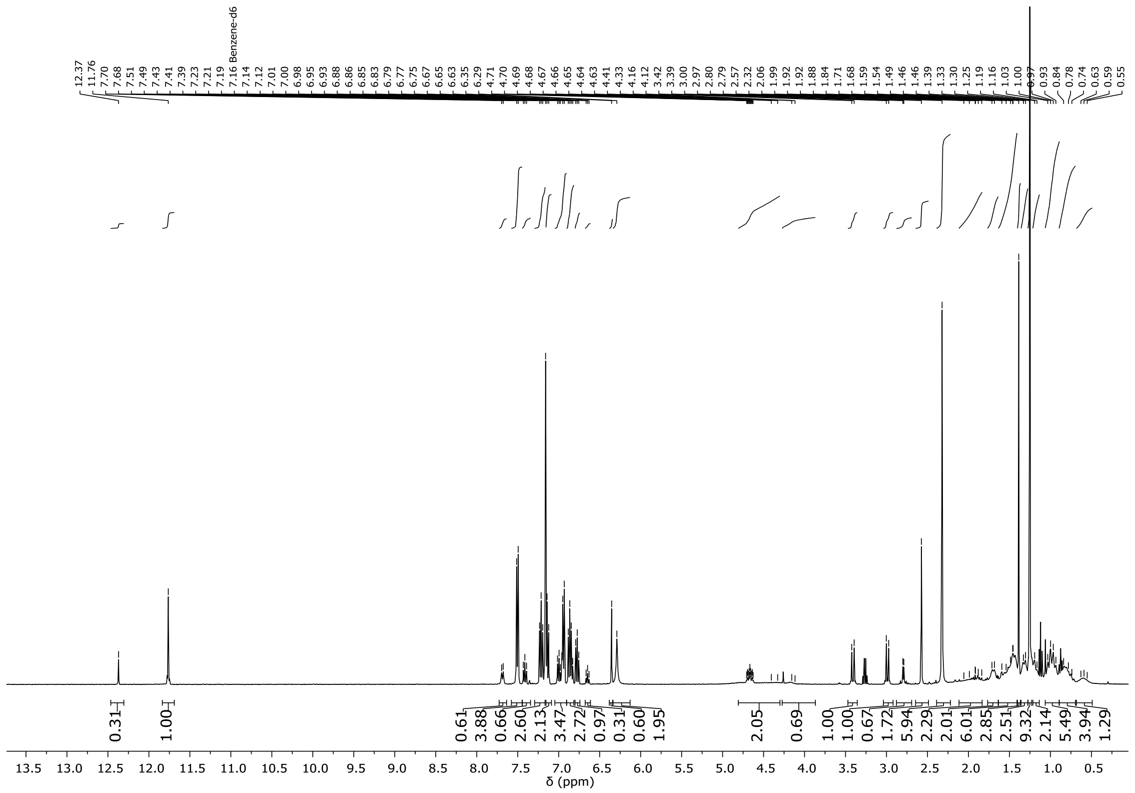

Figure S43. ^{1}H NMR spectrum of W(*N*-2,6-dimethylphenyl)(CHPh)(1,3-dicyclohexylimidazol-2-ylidene)(CH_2Ph)(O^tBu) (**7**) in C_6D_6.

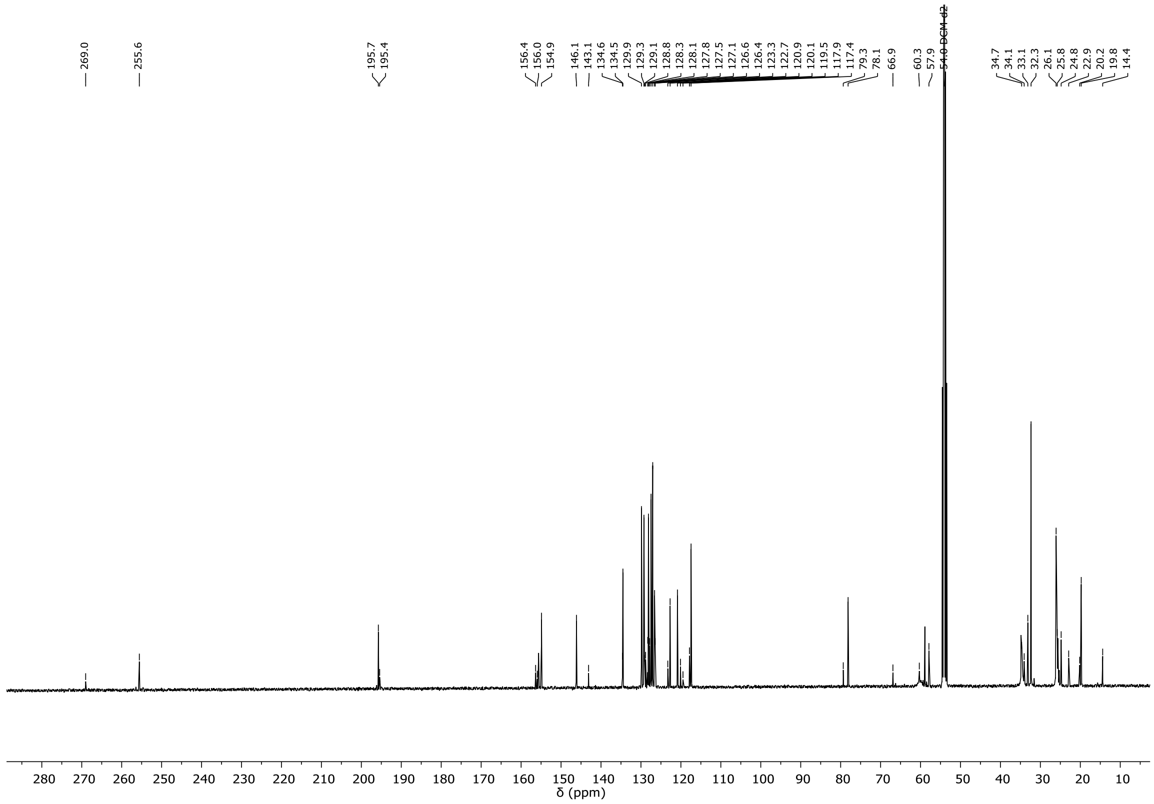

Figure S44. ^{13}C NMR spectrum of W(*N*-2,6-dimethylphenyl)(CHPh)(1,3-dicyclohexylimidazol-2-ylidene)(CH_2Ph)(O^tBu) (**7**) in CD_2Cl_2.

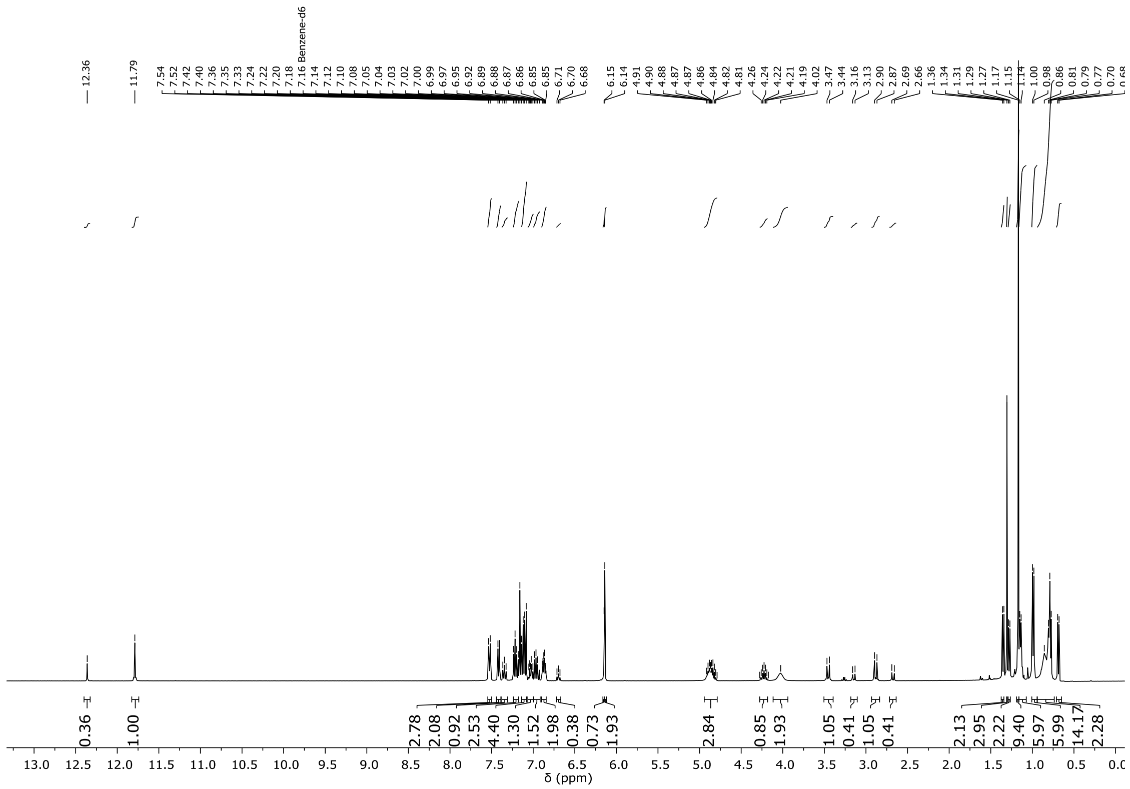

Figure S45. ^{1}H NMR spectrum of W(*N*-2,6-diisopropylphenyl)(CHPh)(1,3-diisopropylimidazol-2-ylidene)(CH_2Ph)(O^tBu) (**11**) in C_6D_6.

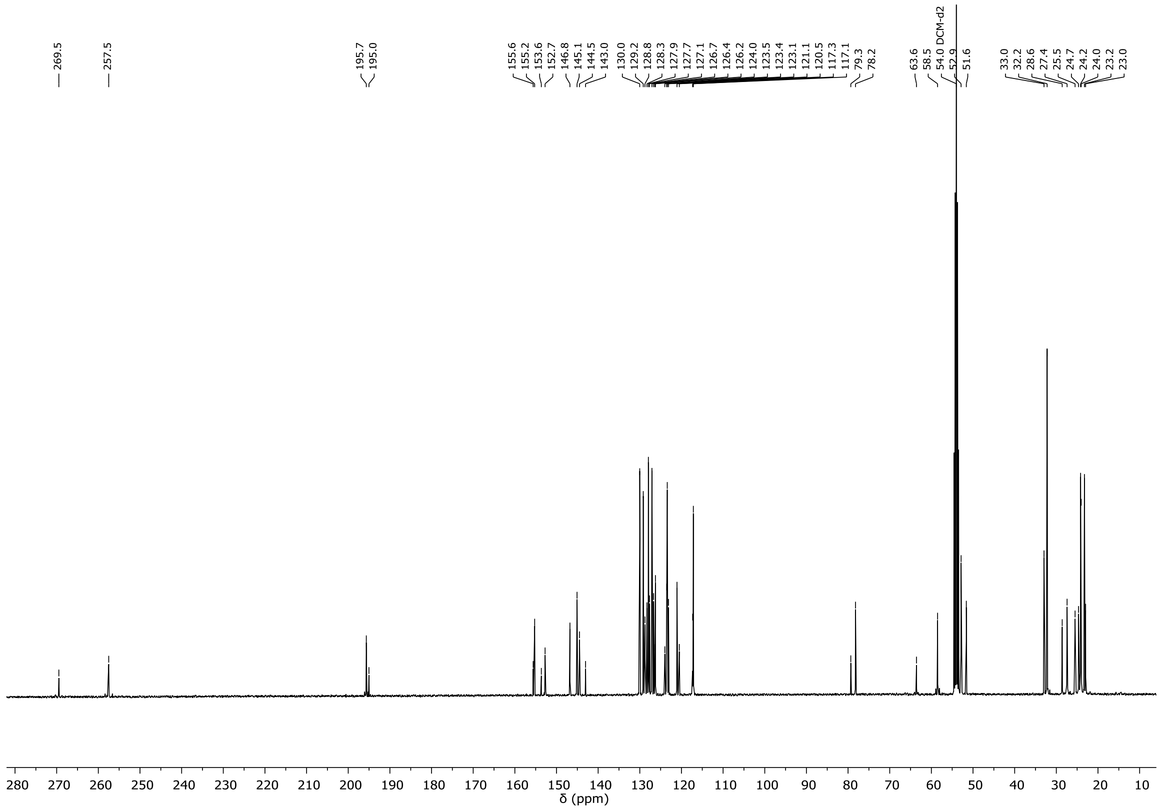

Figure S46. ^{13}C NMR spectrum of W(*N*-2,6-diisopropylphenyl)(CHPh)(1,3-diisopropylimidazol-2-ylidene)(CH_2Ph)(O^tBu) (**11**) in CD_2Cl_2.

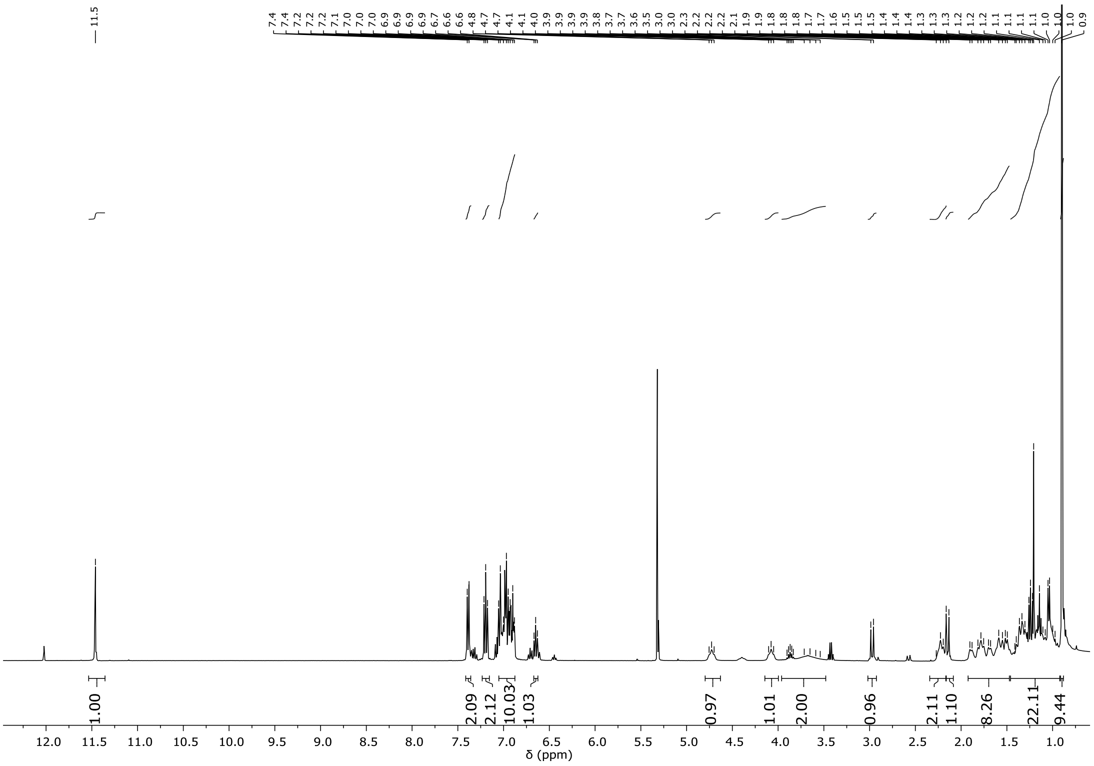

Figure S47. ^{1}H NMR spectrum of W(*N*-2,6-diisopropylphenyl)(CHPh)(1,3-dicyclohexylimidazol-2-ylidene)(CH_2Ph)(O^tBu) (**10**) in C_6D_6 (minor isomer < 15%, not integrated for clarity).

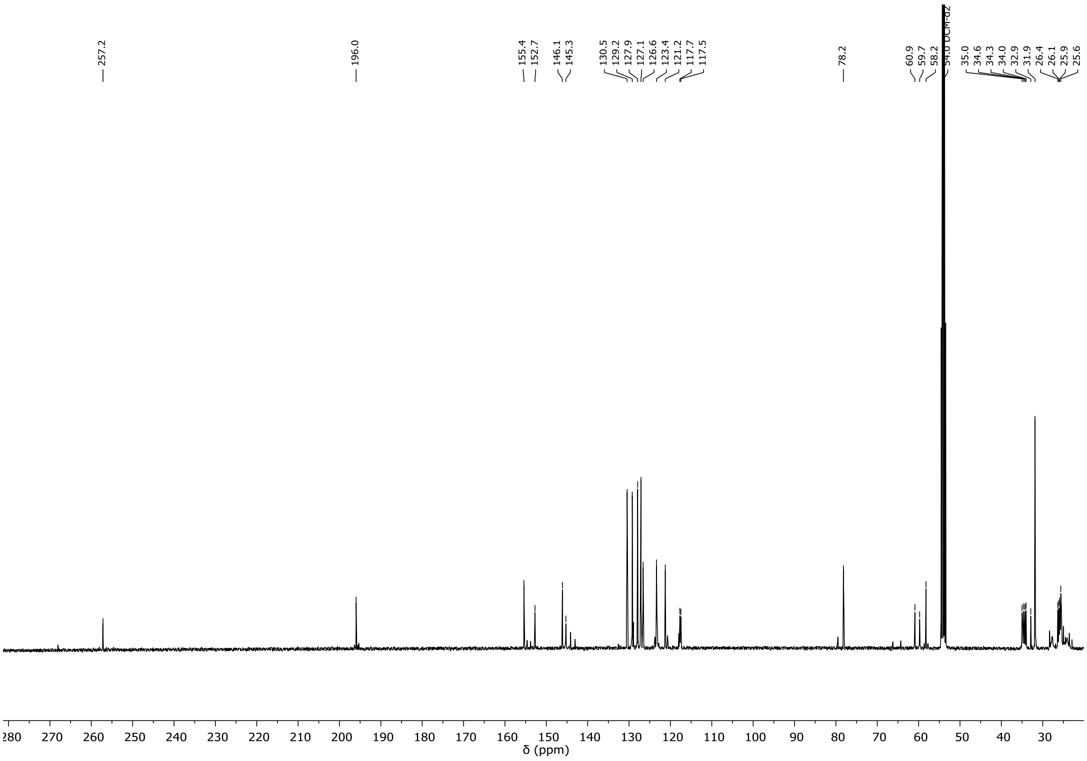

Figure S48. ^{13}C NMR spectrum of W(*N*-2,6-diisopropylphenyl)(CHPh)(1,3-dicyclohexylimidazol-2-ylidene)(CH_2Ph)(O^tBu) (**10**) in CD_2Cl_2 (minor isomer < 15%, peaks not picked for clarity).

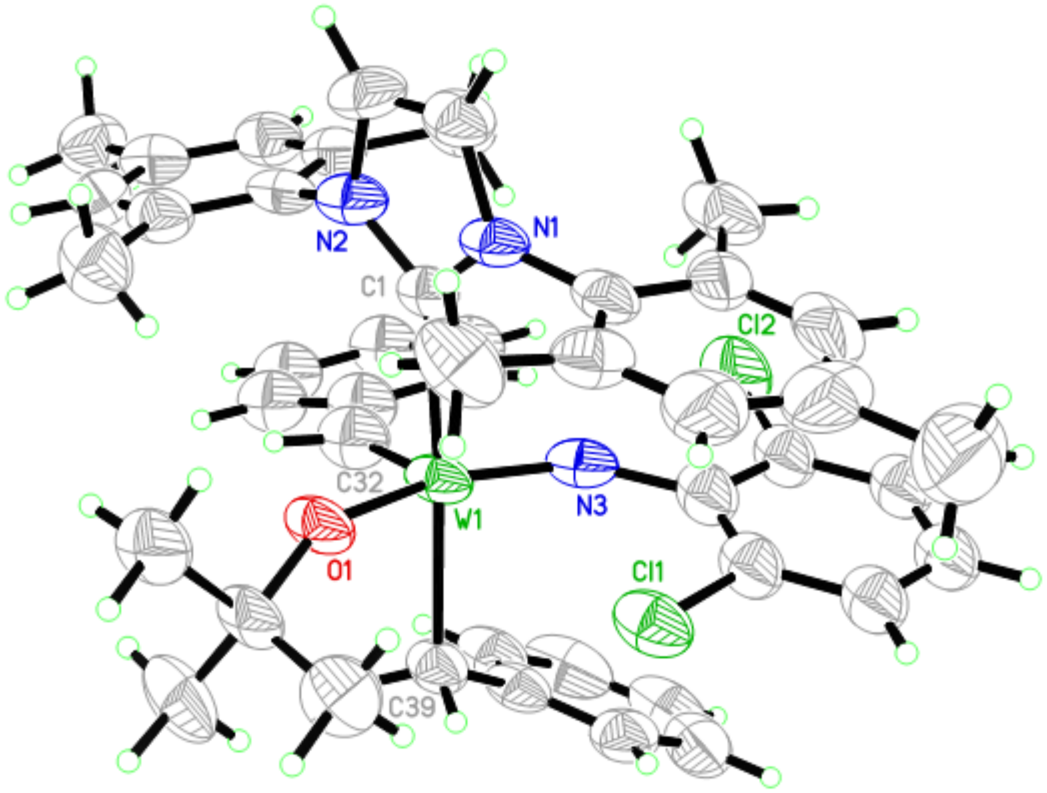

Figure S49. Single crystal X-ray structure of **2** displayed as a thermal ellipsoid plot (50% probability). Relevant bond lengths (pm) and angles (°): W(1)-N(3) 1.704(4), W(1)-O(1) 1.920(4), W(1)-C(32) 1.911(5), W(1)-C(39) 2.250(7), W(1)-C(1) 2.312(6), C(1)-N(1) 1.373(9), C(1)-N(2) 1.371(9); N(3)-W(1)-O(1) 148.9(3), N(3)-W(1)-C(32) 104.9(4), O(1)-W(1)-C(32) 106.1(3), N(3)-W(1)-C(39) 91.7(2), O(1)-W(1)-C(39) 87.3(2), C(32)-W(1)-C(39) 90.0(4), N(3)-W(1)-C(1) 94.3(2), O(1)-W(1)-C(1) 83.1(2), C(32)-W(1)-C(1) 96.8(3), C(39)-W(1)-C(1) 169.5(2), N(1)-C(1)-N(2) 102.5(5).

Table S1. Crystal data and structure refinement for **2**.

Empirical formula	$C_{45}H_{49}Cl_2N_3OW$
Formula weight	902.62
Temperature	135(2) K
Wavelength	1.54178 Å
Crystal system, space group	Monoclinic, $P2_1/n$
Unit cell dimensions	a = 10.3121(3) Å, α = 90°<br>b = 21.0493(8) Å, β = 91.105(2)°<br>c = 18.9691(5) Å, γ = 90°
Volume	4116.7(2) $Å^3$
Z, Calculated density	4, 1.456 mg/m^3
Absorption coefficient	6.673 mm^{-1}
F(000)	1824
Crystal size	0.064 x 0.061 x 0.029 mm
Theta range for data collection	3.136 to 65.598°
Limiting indices	-12<=h<=9, -24<=k<=24, -22<=l<=22
Reflections collected / unique	31175 / 6939 [R(int) = 0.0747]
Completeness to Θ = 65.598	97.70%
Absorption correction	Numerical
Max. and min. transmission	0.9018 and 0.6750
Refinement method	Full-matrix least-squares on F^2
Data / restraints / parameters	6949 / 509 / 623
Goodness-of-fit on F^2	1.040
Final R indices [I>2σ(I)]	R1 = 0.0515, wR2 = 0.1166

R indices (all data)	R1 = 0.0845, wR2 = 0.1278
Extinction coefficient	n/a
Largest diff. peak and hole	1.605 and -1.214 e.Å^{-3}

Table S2. Atomic coordinates (x 10^4) and equivalent isotropic displacement parameters (Å^2 x 10^3) for **2**. U(eq) is defined as one third of the trace of the orthogonalized U_{ij} tensor.

	x	y	z	U(eq)
W(1)	6642(1)	1416(1)	2545(1)	47(1)
C(1)	4424(6)	1450(3)	2349(3)	40(2)
N(1)	3588(5)	971(3)	2151(3)	43(1)
O(1)	6688(5)	1583(3)	1551(2)	55(1)
N(2)	3618(5)	1967(3)	2378(3)	47(1)
C(2)	2320(7)	1180(4)	2065(4)	51(2)
C(3)	2337(7)	1807(4)	2208(4)	56(2)
C(4)	3870(6)	300(4)	2117(4)	48(2)
C(5)	4058(7)	25(4)	1438(4)	58(2)
C(6)	4267(8)	-607(4)	1409(6)	76(3)
C(7)	4273(8)	-991(5)	1991(7)	87(3)
C(8)	4024(8)	-701(5)	2649(6)	90(4)
C(9)	3815(7)	-54(4)	2726(4)	59(2)
C(10)	3976(9)	416(4)	789(4)	73(3)
C(11)	4514(11)	-1699(5)	1972(7)	114(4)
C(12)	3506(8)	257(5)	3421(4)	75(3)
C(13)	3898(6)	2579(4)	2668(3)	47(2)
C(14)	4195(7)	3089(4)	2220(4)	54(2)
C(15)	4353(7)	3682(4)	2517(4)	58(2)
C(16)	4225(7)	3791(4)	3228(4)	56(2)
C(17)	3941(7)	3274(4)	3655(4)	55(2)
C(18)	3757(7)	2674(4)	3392(4)	50(2)
C(19)	4350(9)	2982(4)	1435(4)	71(2)
C(20)	4406(8)	4447(4)	3537(5)	69(2)
C(21)	3422(7)	2117(4)	3858(4)	61(2)
Cl(1)	7257(2)	-110(1)	2144(1)	61(1)
Cl(2)	6206(3)	678(2)	4791(1)	78(1)
N(3)	6558(5)	879(3)	3214(3)	48(2)
C(22)	6746(8)	232(3)	3487(4)	57(1)
C(23)	7073(8)	-255(4)	3041(3)	58(1)

C(24)	7277(9)	-876(4)	3276(4)	60(1)
C(25)	7144(10)	-1006(4)	3986(4)	61(1)
C(26)	6811(11)	-532(4)	4437(4)	60(1)
C(27)	6614(8)	83(4)	4194(4)	58(1)
C(32)	6795(9)	2223(3)	3001(4)	62(2)
C(33)	6907(9)	2566(4)	3688(4)	63(1)
C(34)	7205(11)	3214(4)	3739(4)	64(1)
C(35)	7304(11)	3504(4)	4398(4)	66(1)
C(36)	7093(11)	3162(4)	4995(5)	67(2)
C(37)	6800(12)	2530(5)	4958(4)	66(2)
C(38)	6704(10)	2233(4)	4310(4)	65(1)
Cl(1A)	7244(8)	2829(5)	2718(5)	85(3)
Cl(2A)	6023(10)	1368(5)	4966(4)	89(3)
N(3A)	6479(15)	1623(7)	3404(3)	41(5)
C(22A)	6663(15)	2166(6)	3897(6)	77(7)
C(23A)	7015(16)	2755(7)	3630(6)	40(7)
C(24A)	7200(30)	3273(8)	4063(9)	106(17)
C(25A)	7010(30)	3212(9)	4784(10)	160(30)
C(26A)	6650(30)	2627(9)	5060(9)	110(20)
C(27A)	6476(17)	2111(6)	4618(8)	77(7)
C(32A)	6700(30)	525(4)	2716(15)	65(4)
C(33A)	6838(19)	-63(9)	3161(12)	65(4)
C(34A)	7100(30)	-693(11)	2965(9)	65(4)
C(35A)	7190(30)	-1177(9)	3483(12)	65(4)
C(36A)	6990(40)	-1026(10)	4201(11)	65(4)
C(37A)	6760(40)	-391(12)	4401(11)	65(4)
C(38A)	6650(30)	85(9)	3885(13)	65(4)
C(28)	7318(7)	1585(4)	884(3)	53(2)
C(29)	8504(9)	2014(5)	912(4)	79(3)
C(30)	7690(9)	917(4)	695(4)	75(3)
C(31)	6346(8)	1842(5)	336(3)	70(2)
C(39)	8817(7)	1338(4)	2524(3)	51(2)
C(40)	9422(6)	1163(4)	3202(3)	47(2)
C(41)	9617(7)	1612(5)	3739(4)	65(2)
C(42)	10105(8)	1423(6)	4401(4)	83(3)
C(43)	10404(8)	801(7)	4530(5)	90(4)
C(44)	10232(8)	362(5)	3998(5)	84(3)
C(45)	9763(7)	541(4)	3351(4)	63(2)

Table S3. Bond lengths [Å] and angles [°] for **2**.

W(1)-N(3A)	1.699(5)
W(1)-N(3)	1.704(4)
W(1)-C(32A)	1.905(5)
W(1)-C(32)	1.911(5)
W(1)-O(1)	1.920(4)

W(1)-C(39)	2.250(7)
W(1)-C(1)	2.312(6)
C(1)-N(1)	1.373(9)
C(1)-N(2)	1.371(9)
N(1)-C(2)	1.387(8)
N(1)-C(4)	1.444(9)
O(1)-C(28)	1.432(7)
N(2)-C(3)	1.395(8)
N(2)-C(13)	1.429(9)
C(2)-C(3)	1.347(11)
C(2)-H(2)	0.9500
C(3)-H(3)	0.9500
C(4)-C(9)	1.375(10)
C(4)-C(5)	1.430(10)
C(5)-C(6)	1.349(12)
C(5)-C(10)	1.481(11)
C(6)-C(7)	1.369(14)
C(6)-H(6)	0.9500
C(7)-C(8)	1.416(14)
C(7)-C(11)	1.510(14)
C(8)-C(9)	1.388(14)
C(8)-H(8)	0.9500
C(9)-C(12)	1.511(12)
C(10)-H(10A)	0.9800
C(10)-H(10B)	0.9800
C(10)-H(10C)	0.9800
C(11)-H(11A)	0.9800
C(11)-H(11B)	0.9800
C(11)-H(11C)	0.9800
C(12)-H(12A)	0.9800
C(12)-H(12B)	0.9800
C(12)-H(12C)	0.9800
C(13)-C(18)	1.398(9)
C(13)-C(14)	1.407(10)
C(14)-C(15)	1.378(11)
C(14)-C(19)	1.516(9)
C(15)-C(16)	1.377(10)
C(15)-H(15)	0.9500
C(16)-C(17)	1.391(11)
C(16)-C(20)	1.510(11)
C(17)-C(18)	1.369(10)
C(17)-H(17)	0.9500
C(18)-C(21)	1.513(10)
C(19)-H(19A)	0.9800
C(19)-H(19B)	0.9800
C(19)-H(19C)	0.9800

C(20)-H(20A)	0.9800
C(20)-H(20B)	0.9800
C(20)-H(20C)	0.9800
C(21)-H(21A)	0.9800
C(21)-H(21B)	0.9800
C(21)-H(21C)	0.9800
Cl(1)-C(23)	1.743(6)
Cl(2)-C(27)	1.745(7)
N(3)-C(22)	1.467(7)
C(22)-C(23)	1.376(7)
C(22)-C(27)	1.387(8)
C(23)-C(24)	1.395(9)
C(24)-C(25)	1.384(9)
C(24)-H(24)	0.9500
C(25)-C(26)	1.362(10)
C(25)-H(25)	0.9500
C(26)-C(27)	1.389(9)
C(26)-H(26)	0.9500
C(32)-C(33)	1.493(7)
C(32)-H(32)	0.9500
C(33)-C(38)	1.391(10)
C(33)-C(34)	1.402(7)
C(34)-C(35)	1.392(10)
C(34)-H(34)	0.9500
C(35)-C(36)	1.364(11)
C(35)-H(35)	0.9500
C(36)-C(37)	1.365(10)
C(36)-H(36)	0.9500
C(37)-C(38)	1.381(10)
C(37)-H(37)	0.9500
C(38)-H(38)	0.9500
Cl(1A)-C(23A)	1.757(9)
Cl(2A)-C(27A)	1.765(9)
N(3A)-C(22A)	1.487(9)
C(22A)-C(27A)	1.388(12)
C(22A)-C(23A)	1.389(8)
C(23A)-C(24A)	1.376(12)
C(24A)-C(25A)	1.391(13)
C(24A)-H(24A)	0.9500
C(25A)-C(26A)	1.392(14)
C(25A)-H(25A)	0.9500
C(26A)-C(27A)	1.381(13)
C(26A)-H(26A)	0.9500
C(32A)-C(33A)	1.503(10)
C(32A)-H(32A)	0.9500
C(33A)-C(34A)	1.404(8)

C(33A)-C(38A)	1.425(14)
C(34A)-C(35A)	1.419(13)
C(34A)-H(34A)	0.9500
C(35A)-C(36A)	1.417(13)
C(35A)-H(35A)	0.9500
C(36A)-C(37A)	1.410(14)
C(36A)-H(36A)	0.9500
C(37A)-C(38A)	1.405(14)
C(37A)-H(37A)	0.9500
C(38A)-H(38A)	0.9500
C(28)-C(30)	1.504(11)
C(28)-C(29)	1.520(11)
C(28)-C(31)	1.529(10)
C(29)-H(29A)	0.9800
C(29)-H(29B)	0.9800
C(29)-H(29C)	0.9800
C(30)-H(30A)	0.9800
C(30)-H(30B)	0.9800
C(30)-H(30C)	0.9800
C(31)-H(31A)	0.9800
C(31)-H(31B)	0.9800
C(31)-H(31C)	0.9800
C(39)-C(40)	1.466(9)
C(39)-H(39A)	0.9900
C(39)-H(39B)	0.9900
C(40)-C(45)	1.384(11)
C(40)-C(41)	1.400(11)
C(41)-C(42)	1.402(11)
C(41)-H(41)	0.9500
C(42)-C(43)	1.368(15)
C(42)-H(42)	0.9500
C(43)-C(44)	1.377(15)
C(43)-H(43)	0.9500
C(44)-C(45)	1.364(11)
C(44)-H(44)	0.9500
C(45)-H(45)	0.9500
N(3A)-W(1)-C(32A)	95.3(10)
N(3)-W(1)-C(32)	104.9(4)
N(3A)-W(1)-O(1)	154.2(5)
N(3)-W(1)-O(1)	148.9(3)
C(32A)-W(1)-O(1)	110.3(9)
C(32)-W(1)-O(1)	106.1(3)
N(3A)-W(1)-C(39)	98.8(5)
N(3)-W(1)-C(39)	91.7(2)
C(32A)-W(1)-C(39)	84.5(9)

C(32)-W(1)-C(39)	90.0(4)
O(1)-W(1)-C(39)	87.3(2)
N(3A)-W(1)-C(1)	91.7(5)
N(3)-W(1)-C(1)	94.3(2)
C(32A)-W(1)-C(1)	94.9(9)
C(32)-W(1)-C(1)	96.8(3)
O(1)-W(1)-C(1)	83.1(2)
C(39)-W(1)-C(1)	169.5(2)
N(1)-C(1)-N(2)	102.5(5)
N(1)-C(1)-W(1)	129.4(5)
N(2)-C(1)-W(1)	128.0(5)
C(1)-N(1)-C(2)	112.6(6)
C(1)-N(1)-C(4)	127.2(5)
C(2)-N(1)-C(4)	119.7(6)
C(28)-O(1)-W(1)	152.6(4)
C(1)-N(2)-C(3)	111.8(6)
C(1)-N(2)-C(13)	127.9(5)
C(3)-N(2)-C(13)	119.3(6)
C(3)-C(2)-N(1)	106.2(6)
C(3)-C(2)-H(2)	126.9
N(1)-C(2)-H(2)	126.9
C(2)-C(3)-N(2)	106.9(6)
C(2)-C(3)-H(3)	126.5
N(2)-C(3)-H(3)	126.5
C(9)-C(4)-C(5)	123.1(8)
C(9)-C(4)-N(1)	118.7(7)
C(5)-C(4)-N(1)	117.8(6)
C(6)-C(5)-C(4)	117.4(8)
C(6)-C(5)-C(10)	121.4(8)
C(4)-C(5)-C(10)	121.1(7)
C(5)-C(6)-C(7)	123.3(10)
C(5)-C(6)-H(6)	118.4
C(7)-C(6)-H(6)	118.4
C(6)-C(7)-C(8)	117.2(9)
C(6)-C(7)-C(11)	124.1(11)
C(8)-C(7)-C(11)	118.6(12)
C(9)-C(8)-C(7)	123.2(9)
C(9)-C(8)-H(8)	118.4
C(7)-C(8)-H(8)	118.4
C(4)-C(9)-C(8)	115.7(8)
C(4)-C(9)-C(12)	120.7(8)
C(8)-C(9)-C(12)	123.6(8)
C(5)-C(10)-H(10A)	109.5
C(5)-C(10)-H(10B)	109.5
H(10A)-C(10)-H(10B)	109.5
C(5)-C(10)-H(10C)	109.5

H(10A)-C(10)-H(10C)	109.5
H(10B)-C(10)-H(10C)	109.5
C(7)-C(11)-H(11A)	109.5
C(7)-C(11)-H(11B)	109.5
H(11A)-C(11)-H(11B)	109.5
C(7)-C(11)-H(11C)	109.5
H(11A)-C(11)-H(11C)	109.5
H(11B)-C(11)-H(11C)	109.5
C(9)-C(12)-H(12A)	109.5
C(9)-C(12)-H(12B)	109.5
H(12A)-C(12)-H(12B)	109.5
C(9)-C(12)-H(12C)	109.5
H(12A)-C(12)-H(12C)	109.5
H(12B)-C(12)-H(12C)	109.5
C(18)-C(13)-C(14)	120.9(7)
C(18)-C(13)-N(2)	118.9(6)
C(14)-C(13)-N(2)	120.0(6)
C(15)-C(14)-C(13)	118.0(7)
C(15)-C(14)-C(19)	121.5(7)
C(13)-C(14)-C(19)	120.6(7)
C(16)-C(15)-C(14)	122.6(7)
C(16)-C(15)-H(15)	118.7
C(14)-C(15)-H(15)	118.7
C(15)-C(16)-C(17)	117.7(7)
C(15)-C(16)-C(20)	121.3(8)
C(17)-C(16)-C(20)	121.0(7)
C(18)-C(17)-C(16)	122.6(7)
C(18)-C(17)-H(17)	118.7
C(16)-C(17)-H(17)	118.7
C(17)-C(18)-C(13)	118.2(7)
C(17)-C(18)-C(21)	122.2(6)
C(13)-C(18)-C(21)	119.5(7)
C(14)-C(19)-H(19A)	109.5
C(14)-C(19)-H(19B)	109.5
H(19A)-C(19)-H(19B)	109.5
C(14)-C(19)-H(19C)	109.5
H(19A)-C(19)-H(19C)	109.5
H(19B)-C(19)-H(19C)	109.5
C(16)-C(20)-H(20A)	109.5
C(16)-C(20)-H(20B)	109.5
H(20A)-C(20)-H(20B)	109.5
C(16)-C(20)-H(20C)	109.5
H(20A)-C(20)-H(20C)	109.5
H(20B)-C(20)-H(20C)	109.5
C(18)-C(21)-H(21A)	109.5
C(18)-C(21)-H(21B)	109.5

H(21A)-C(21)-H(21B)	109.5
C(18)-C(21)-H(21C)	109.5
H(21A)-C(21)-H(21C)	109.5
H(21B)-C(21)-H(21C)	109.5
C(22)-N(3)-W(1)	150.5(6)
C(23)-C(22)-C(27)	117.1(5)
C(23)-C(22)-N(3)	120.5(7)
C(27)-C(22)-N(3)	122.5(6)
C(22)-C(23)-C(24)	122.7(5)
C(22)-C(23)-Cl(1)	120.1(6)
C(24)-C(23)-Cl(1)	117.2(6)
C(25)-C(24)-C(23)	118.5(6)
C(25)-C(24)-H(24)	120.8
C(23)-C(24)-H(24)	120.8
C(26)-C(25)-C(24)	119.9(6)
C(26)-C(25)-H(25)	120.0
C(24)-C(25)-H(25)	120.0
C(25)-C(26)-C(27)	120.7(6)
C(25)-C(26)-H(26)	119.6
C(27)-C(26)-H(26)	119.6
C(22)-C(27)-C(26)	121.1(6)
C(22)-C(27)-Cl(2)	119.6(6)
C(26)-C(27)-Cl(2)	119.3(6)
C(33)-C(32)-W(1)	146.0(7)
C(33)-C(32)-H(32)	107.0
W(1)-C(32)-H(32)	107.0
C(38)-C(33)-C(34)	117.9(6)
C(38)-C(33)-C(32)	119.1(8)
C(34)-C(33)-C(32)	123.0(8)
C(35)-C(34)-C(33)	120.0(6)
C(35)-C(34)-H(34)	120.0
C(33)-C(34)-H(34)	120.0
C(36)-C(35)-C(34)	120.3(6)
C(36)-C(35)-H(35)	119.8
C(34)-C(35)-H(35)	119.8
C(35)-C(36)-C(37)	120.6(6)
C(35)-C(36)-H(36)	119.7
C(37)-C(36)-H(36)	119.7
C(36)-C(37)-C(38)	120.0(7)
C(36)-C(37)-H(37)	120.0
C(38)-C(37)-H(37)	120.0
C(37)-C(38)-C(33)	121.1(6)
C(37)-C(38)-H(38)	119.4
C(33)-C(38)-H(38)	119.4
C(22A)-N(3A)-W(1)	141.9(11)
C(27A)-C(22A)-C(23A)	118.4(6)

C(27A)-C(22A)-N(3A)	122.5(9)
C(23A)-C(22A)-N(3A)	119.1(9)
C(24A)-C(23A)-C(22A)	121.6(7)
C(24A)-C(23A)-Cl(1A)	119.8(9)
C(22A)-C(23A)-Cl(1A)	118.6(8)
C(23A)-C(24A)-C(25A)	119.5(7)
C(23A)-C(24A)-H(24A)	120.3
C(25A)-C(24A)-H(24A)	120.3
C(24A)-C(25A)-C(26A)	119.7(7)
C(24A)-C(25A)-H(25A)	120.1
C(26A)-C(25A)-H(25A)	120.1
C(27A)-C(26A)-C(25A)	119.9(8)
C(27A)-C(26A)-H(26A)	120.0
C(25A)-C(26A)-H(26A)	120.0
C(26A)-C(27A)-C(22A)	120.9(7)
C(26A)-C(27A)-Cl(2A)	120.0(10)
C(22A)-C(27A)-Cl(2A)	119.1(10)
C(33A)-C(32A)-W(1)	155(2)
C(33A)-C(32A)-H(32A)	102.3
W(1)-C(32A)-H(32A)	102.3
C(34A)-C(33A)-C(38A)	119.6(7)
C(34A)-C(33A)-C(32A)	130(2)
C(38A)-C(33A)-C(32A)	110(2)
C(33A)-C(34A)-C(35A)	120.3(7)
C(33A)-C(34A)-H(34A)	119.8
C(35A)-C(34A)-H(34A)	119.8
C(36A)-C(35A)-C(34A)	119.7(7)
C(36A)-C(35A)-H(35A)	120.2
C(34A)-C(35A)-H(35A)	120.2
C(37A)-C(36A)-C(35A)	120.0(7)
C(37A)-C(36A)-H(36A)	120.0
C(35A)-C(36A)-H(36A)	120.0
C(38A)-C(37A)-C(36A)	120.0(8)
C(38A)-C(37A)-H(37A)	120.0
C(36A)-C(37A)-H(37A)	120.0
C(37A)-C(38A)-C(33A)	120.2(8)
C(37A)-C(38A)-H(38A)	119.9
C(33A)-C(38A)-H(38A)	119.9
O(1)-C(28)-C(30)	109.3(6)
O(1)-C(28)-C(29)	110.5(6)
C(30)-C(28)-C(29)	110.7(7)
O(1)-C(28)-C(31)	107.4(6)
C(30)-C(28)-C(31)	109.6(6)
C(29)-C(28)-C(31)	109.3(7)
C(28)-C(29)-H(29A)	109.5
C(28)-C(29)-H(29B)	109.5

H(29A)-C(29)-H(29B)	109.5
C(28)-C(29)-H(29C)	109.5
H(29A)-C(29)-H(29C)	109.5
H(29B)-C(29)-H(29C)	109.5
C(28)-C(30)-H(30A)	109.5
C(28)-C(30)-H(30B)	109.5
H(30A)-C(30)-H(30B)	109.5
C(28)-C(30)-H(30C)	109.5
H(30A)-C(30)-H(30C)	109.5
H(30B)-C(30)-H(30C)	109.5
C(28)-C(31)-H(31A)	109.5
C(28)-C(31)-H(31B)	109.5
H(31A)-C(31)-H(31B)	109.5
C(28)-C(31)-H(31C)	109.5
H(31A)-C(31)-H(31C)	109.5
H(31B)-C(31)-H(31C)	109.5
C(40)-C(39)-W(1)	114.2(5)
C(40)-C(39)-H(39A)	108.7
W(1)-C(39)-H(39A)	108.7
C(40)-C(39)-H(39B)	108.7
W(1)-C(39)-H(39B)	108.7
H(39A)-C(39)-H(39B)	107.6
C(45)-C(40)-C(41)	117.2(7)
C(45)-C(40)-C(39)	121.3(7)
C(41)-C(40)-C(39)	121.4(7)
C(40)-C(41)-C(42)	120.3(9)
C(40)-C(41)-H(41)	119.8
C(42)-C(41)-H(41)	119.8
C(43)-C(42)-C(41)	120.5(9)
C(43)-C(42)-H(42)	119.8
C(41)-C(42)-H(42)	119.8
C(44)-C(43)-C(42)	119.1(8)
C(44)-C(43)-H(43)	120.4
C(42)-C(43)-H(43)	120.4
C(45)-C(44)-C(43)	120.8(10)
C(45)-C(44)-H(44)	119.6
C(43)-C(44)-H(44)	119.6
C(44)-C(45)-C(40)	122.1(9)
C(44)-C(45)-H(45)	119.0
C(40)-C(45)-H(45)	119.0

Table S4. Anisotropic displacement parameters ($Å^2 \times 10^3$) for **2**. The anisotropic displacement factor exponent takes the form: $-2\pi^2 [h^2 a^{*2} U_{11} + ... + 2 h k a^* b^* U_{12}]$.

	U11	U22	U33	U23	U13	U12
W(1)	33(1)	82(1)	26(1)	6(1)	3(1)	4(1)
C(1)	35(3)	65(4)	20(3)	4(3)	2(2)	4(4)
N(1)	26(3)	68(4)	34(3)	2(3)	0(2)	1(3)
O(1)	44(3)	92(4)	30(2)	14(2)	9(2)	4(3)
N(2)	33(3)	72(4)	35(3)	-7(3)	0(2)	3(3)
C(2)	33(4)	68(5)	53(4)	1(4)	-5(3)	-7(3)
C(3)	34(4)	82(6)	51(4)	-2(4)	-8(3)	13(4)
C(4)	27(3)	66(5)	50(4)	7(3)	2(3)	-8(3)
C(5)	40(4)	66(5)	67(5)	-15(4)	3(4)	-3(4)
C(6)	47(5)	70(6)	111(7)	-5(5)	-3(5)	-12(5)
C(7)	38(5)	65(6)	159(11)	-14(7)	-13(6)	-8(4)
C(8)	31(5)	111(9)	128(9)	63(7)	-15(5)	-19(5)
C(9)	33(4)	68(5)	77(5)	24(4)	-6(4)	-4(4)
C(10)	87(6)	91(6)	43(4)	-11(4)	18(4)	-18(5)
C(11)	88(7)	84(6)	170(8)	3(6)	-15(6)	-7(6)
C(12)	48(5)	128(8)	50(4)	33(5)	5(4)	-5(5)
C(13)	28(3)	69(5)	44(4)	-7(3)	6(3)	2(3)
C(14)	41(4)	77(5)	44(4)	4(4)	0(3)	5(4)
C(15)	46(4)	66(6)	61(5)	15(4)	0(4)	4(4)
C(16)	40(4)	71(5)	58(5)	-9(4)	5(3)	5(4)
C(17)	47(4)	74(5)	43(4)	-7(4)	5(3)	6(4)
C(18)	37(4)	67(5)	47(4)	-1(3)	5(3)	8(4)
C(19)	81(6)	87(6)	46(4)	2(4)	1(4)	-4(5)
C(20)	63(5)	62(5)	82(6)	-7(4)	4(4)	8(4)
C(21)	61(5)	78(6)	44(4)	-8(4)	11(4)	1(4)
Cl(1)	49(1)	93(2)	41(1)	4(1)	1(1)	-7(1)
Cl(2)	91(2)	109(2)	35(1)	21(1)	11(1)	1(2)
N(3)	32(4)	73(5)	40(4)	-8(3)	1(3)	2(4)
C(22)	47(3)	75(3)	49(2)	20(2)	-3(2)	-3(2)
C(23)	48(3)	75(3)	51(2)	20(2)	-4(2)	-4(2)
C(24)	51(3)	76(3)	53(2)	19(2)	-4(2)	-3(2)
C(25)	51(3)	77(3)	54(3)	20(2)	-4(2)	-3(2)
C(26)	50(3)	77(3)	51(2)	21(2)	-4(2)	-3(2)
C(27)	48(3)	77(3)	50(2)	21(2)	-3(2)	-3(2)
C(32)	58(3)	82(3)	46(3)	-3(3)	0(3)	2(3)
C(33)	61(3)	82(3)	47(2)	-6(2)	0(2)	2(2)
C(34)	62(3)	81(3)	49(2)	-8(2)	0(2)	2(2)
C(35)	65(3)	83(3)	50(2)	-9(2)	-1(2)	2(3)
C(36)	66(3)	84(3)	50(2)	-9(2)	-2(2)	1(3)

C(37)	66(3)	84(3)	49(2)	-8(2)	-2(2)	0(3)
C(38)	64(3)	84(3)	48(2)	-6(2)	-1(2)	1(2)
Cl(1A)	43(5)	84(6)	128(8)	43(6)	-8(5)	-7(4)
Cl(2A)	111(8)	120(8)	35(4)	-7(4)	5(4)	12(6)
N(3A)	26(8)	39(9)	58(9)	22(7)	2(7)	6(7)
C(22A)	75(9)	82(9)	74(9)	-8(7)	-1(7)	1(7)
C(23A)	33(10)	45(11)	41(10)	-7(8)	-9(8)	5(8)
C(24A)	104(19)	103(19)	111(19)	-6(10)	2(10)	-1(10)
C(25A)	170(30)	160(30)	160(30)	1(10)	0(10)	-2(10)
C(26A)	110(20)	110(20)	110(20)	-7(10)	-2(10)	4(10)
C(27A)	75(9)	82(9)	75(9)	-8(7)	-1(7)	1(7)
C(32A)	63(5)	70(5)	61(5)	8(4)	0(4)	-1(4)
C(33A)	64(5)	69(5)	61(5)	8(4)	0(4)	-1(4)
C(34A)	64(5)	69(5)	61(5)	9(4)	0(4)	-1(4)
C(35A)	64(5)	69(5)	61(5)	9(4)	0(4)	-1(4)
C(36A)	65(5)	70(5)	61(5)	9(4)	0(4)	-1(4)
C(37A)	65(5)	70(5)	61(5)	9(4)	0(4)	-1(4)
C(38A)	64(5)	70(5)	61(5)	9(4)	0(4)	-1(4)
C(28)	57(5)	80(6)	22(3)	8(3)	13(3)	1(4)
C(29)	87(6)	112(8)	39(4)	17(4)	8(4)	-27(6)
C(30)	98(7)	98(7)	29(3)	2(4)	13(4)	17(6)
C(31)	70(6)	109(7)	32(4)	17(4)	4(4)	9(5)
C(39)	33(3)	74(5)	45(4)	14(3)	-1(3)	-2(4)
C(40)	25(3)	77(5)	40(4)	3(3)	-2(3)	-9(3)
C(41)	39(4)	102(7)	55(5)	6(4)	2(3)	-10(4)
C(42)	52(5)	151(10)	47(5)	-7(6)	0(4)	-19(6)
C(43)	46(5)	171(12)	55(5)	46(7)	-9(4)	-13(7)
C(44)	43(5)	120(8)	89(7)	55(6)	-2(5)	4(5)
C(45)	37(4)	90(6)	62(5)	17(4)	2(4)	8(4)

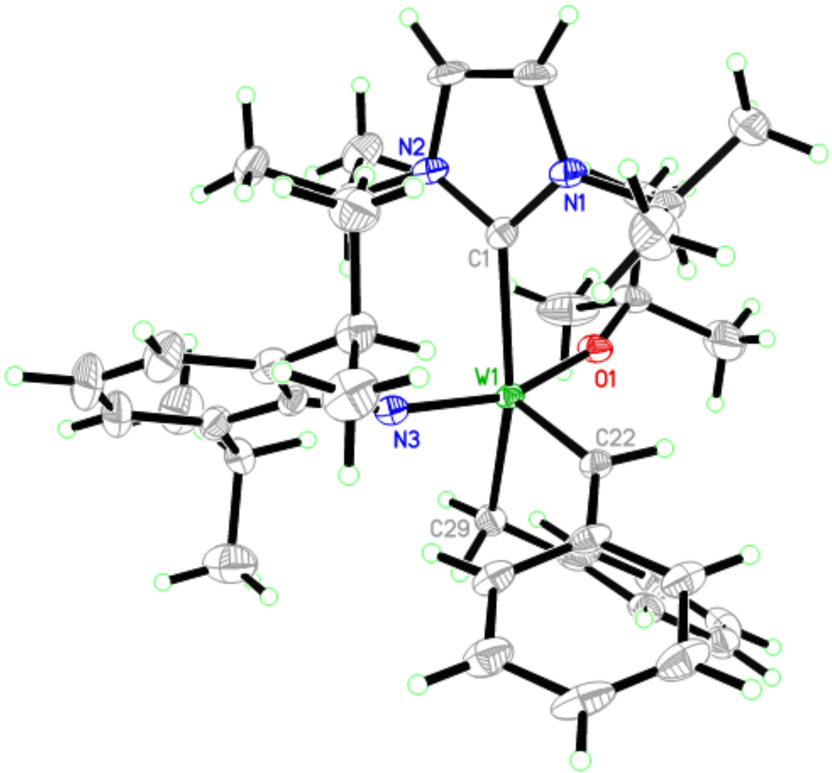

Figure S50. Single crystal X-ray structure of **11** displayed as a thermal ellipsoid plot (50% probability). Relevant bond lengths (pm) and angles (°): W(1)-N(3) 1.7756(19), W(1)-O(1) 1.9049(16), W(1)-C(22) 1.912(2), W(1)-C(29) 2.225(2), W(1)-C(1) 2.279(2), C(1)-N(1) 1.359(3), C(1)-N(2), N(3)-W(1)-O(1) 154.72(8), N(3)-W(1)-C(22) 101.39(10), O(1)-W(1)-C(22) 103.70(9), N(3)-W(1)-C(29) 94.02(9), O(1)-W(1)-C(29) 86.79(8), C(22)-W(1)-C(29) 95.31(10), N(3)-W(1)-C(1) 87.05(8), O(1)-W(1)-C(1) 82.20(8), C(22)-W(1)-C(1) 108.30(9), C(29)-W(1)-C(1) 155.70(9), N(1)-C(1)-N(2) 103.7(2).

Table S5. Crystal data and structure refinement for **11**.

Empirical formula	$C_{39}H_{55}N_3OW$
Formula weight	765.71
Temperature	135(2) K
Wavelength	0.71073 Å
Crystal system, space group	Monoclinic, $P2_1/c$
Unit cell dimensions	a = 20.5371(9) Å, α = 90°<br>b = 9.6156(4) Å, β = 112.431(2)°<br>c = 20.2487(9) Å, γ = 90°
Volume	3696.1(3) A^3
Z, Calculated density	4, 1.376 mg/m^3
Absorption coefficient	3.157 mm^{-1}
F(000)	1568
Crystal size	0.266 x 0.211 x 0.144 mm
Theta range for data collection	2.146 to 30.551 deg.
Limiting indices	-29<=h<=19, -13<=k<=11, -14<=l<=28
Reflections collected / unique	51241 / 11284 [R(int) = 0.0476]
Completeness to Θ = 25.242	99.80%
Absorption correction	Numerical
Max. and min. transmission	0.7277 and 0.5052
Refinement method	Full-matrix least-squares on F^2
Data / restraints / parameters	11284 / 0 / 408
Goodness-of-fit on F^2	1.003
Final R indices [I>2σ(I)]	R1 = 0.0304, wR2 = 0.0506

R indices (all data)	R1 = 0.0540, wR2 = 0.0542
Extinction coefficient	n/a
Largest diff. peak and hole	0.784 and -0.775 e.Å^{-3}

Table S6. Atomic coordinates (x 10^4) and equivalent isotropic displacement parameters (Å^2 x 10^3) for **11**. U(eq) is defined as one third of the trace of the orthogonalized U_{ij} tensor.

	x	y	z	U(eq)
W(1)	2705(1)	3134(1)	2058(1)	14(1)
C(1)	2561(1)	825(3)	1788(1)	18(1)
O(1)	3423(1)	2449(2)	2907(1)	21(1)
N(1)	2944(1)	-39(2)	1544(1)	20(1)
N(2)	2059(1)	-22(2)	1857(1)	20(1)
C(2)	2680(2)	-1378(3)	1461(2)	28(1)
N(3)	1823(1)	3362(2)	1447(1)	16(1)
C(3)	2132(1)	-1370(3)	1652(2)	26(1)
C(4)	3565(1)	377(3)	1388(1)	23(1)
C(5)	4119(1)	-765(3)	1599(2)	35(1)
C(6)	3339(2)	754(3)	606(2)	38(1)
C(7)	1525(1)	386(3)	2142(1)	24(1)
C(8)	789(1)	36(3)	1614(2)	31(1)
C(9)	1678(2)	-300(3)	2864(2)	33(1)
C(10)	1110(1)	3258(2)	1020(1)	18(1)
C(11)	903(1)	2731(3)	319(1)	23(1)
C(12)	184(2)	2651(3)	-94(2)	34(1)
C(13)	-316(2)	3070(3)	159(2)	41(1)
C(14)	-105(1)	3573(3)	850(2)	32(1)
C(15)	599(1)	3676(3)	1290(1)	23(1)
C(16)	1422(1)	2211(3)	14(1)	26(1)
C(17)	1340(2)	2934(3)	-686(2)	38(1)
C(18)	1366(2)	631(3)	-99(2)	37(1)
C(19)	817(1)	4247(3)	2040(1)	24(1)
C(20)	387(2)	3668(3)	2446(2)	42(1)
C(21)	778(2)	5829(3)	2025(2)	47(1)
C(22)	3218(1)	3945(2)	1541(1)	19(1)
C(23)	3087(1)	4839(2)	918(1)	21(1)
C(24)	3647(2)	5251(3)	728(2)	27(1)
C(25)	3541(2)	6134(3)	154(2)	34(1)

C(26)	2882(2)	6632(3)	-239(2)	36(1)
C(27)	2323(2)	6246(3)	-58(2)	32(1)
C(28)	2423(1)	5371(3)	514(1)	24(1)
C(29)	2703(1)	5044(2)	2678(1)	21(1)
C(30)	3388(1)	5635(3)	3166(1)	21(1)
C(31)	3581(1)	5634(3)	3903(1)	25(1)
C(32)	4222(2)	6154(3)	4365(2)	32(1)
C(33)	4683(2)	6713(3)	4099(2)	36(1)
C(34)	4504(1)	6763(3)	3362(2)	32(1)
C(35)	3867(1)	6225(3)	2904(2)	25(1)
C(36)	3787(1)	1706(3)	3541(1)	24(1)
C(37)	3805(2)	165(3)	3382(2)	32(1)
C(38)	3416(2)	1943(3)	4055(2)	49(1)
C(39)	4530(2)	2288(3)	3847(2)	49(1)

Table S7. Bond lengths [Å] and angles [°] for **11**.

W(1)-N(3)	1.7756(19)
W(1)-O(1)	1.9049(16)
W(1)-C(22)	1.912(2)
W(1)-C(29)	2.225(2)
W(1)-C(1)	2.279(2)
C(1)-N(1)	1.359(3)
C(1)-N(2)	1.362(3)
O(1)-C(36)	1.409(3)
N(1)-C(2)	1.382(3)
N(1)-C(4)	1.482(3)
N(2)-C(3)	1.387(3)
N(2)-C(7)	1.474(3)
C(2)-C(3)	1.321(4)
C(2)-H(2)	0.95
N(3)-C(10)	1.391(3)
C(3)-H(3)	0.95
C(4)-C(6)	1.513(4)
C(4)-C(5)	1.519(3)
C(4)-H(4)	1
C(5)-H(5A)	0.98
C(5)-H(5B)	0.98
C(5)-H(5C)	0.98
C(6)-H(6A)	0.98
C(6)-H(6B)	0.98
C(6)-H(6C)	0.98
C(7)-C(8)	1.520(3)
C(7)-C(9)	1.523(4)
C(7)-H(7)	1
C(8)-H(8A)	0.98

C(8)-H(8B)	0.98
C(8)-H(8C)	0.98
C(9)-H(9A)	0.98
C(9)-H(9B)	0.98
C(9)-H(9C)	0.98
C(10)-C(11)	1.412(3)
C(10)-C(15)	1.414(3)
C(11)-C(12)	1.393(4)
C(11)-C(16)	1.506(4)
C(12)-C(13)	1.371(4)
C(12)-H(12)	0.95
C(13)-C(14)	1.383(4)
C(13)-H(13)	0.95
C(14)-C(15)	1.384(3)
C(14)-H(14)	0.95
C(15)-C(19)	1.512(4)
C(16)-C(17)	1.529(4)
C(16)-C(18)	1.535(4)
C(16)-H(16)	1
C(17)-H(17A)	0.98
C(17)-H(17B)	0.98
C(17)-H(17C)	0.98
C(18)-H(18A)	0.98
C(18)-H(18B)	0.98
C(18)-H(18C)	0.98
C(19)-C(20)	1.522(4)
C(19)-C(21)	1.523(4)
C(19)-H(19)	1
C(20)-H(20A)	0.98
C(20)-H(20B)	0.98
C(20)-H(20C)	0.98
C(21)-H(21A)	0.98
C(21)-H(21B)	0.98
C(21)-H(21C)	0.98
C(22)-C(23)	1.465(3)
C(22)-H(22)	0.95
C(23)-C(28)	1.394(3)
C(23)-C(24)	1.401(3)
C(24)-C(25)	1.388(4)
C(24)-H(24)	0.95
C(25)-C(26)	1.368(4)
C(25)-H(25)	0.95
C(26)-C(27)	1.384(4)
C(26)-H(26)	0.95
C(27)-C(28)	1.382(3)
C(27)-H(27)	0.95

C(28)-H(28)	0.95
C(29)-C(30)	1.489(3)
C(29)-H(29A)	0.99
C(29)-H(29B)	0.99
C(30)-C(31)	1.390(3)
C(30)-C(35)	1.404(4)
C(31)-C(32)	1.384(4)
C(31)-H(31)	0.95
C(32)-C(33)	1.367(4)
C(32)-H(32)	0.95
C(33)-C(34)	1.393(4)
C(33)-H(33)	0.95
C(34)-C(35)	1.384(3)
C(34)-H(34)	0.95
C(35)-H(35)	0.95
C(36)-C(39)	1.518(4)
C(36)-C(37)	1.520(3)
C(36)-C(38)	1.522(4)
C(37)-H(37A)	0.98
C(37)-H(37B)	0.98
C(37)-H(37C)	0.98
C(38)-H(38A)	0.98
C(38)-H(38B)	0.98
C(38)-H(38C)	0.98
C(39)-H(39A)	0.98
C(39)-H(39B)	0.98
C(39)-H(39C)	0.98
N(3)-W(1)-O(1)	154.72(8)
N(3)-W(1)-C(22)	101.39(10)
O(1)-W(1)-C(22)	103.70(9)
N(3)-W(1)-C(29)	94.02(9)
O(1)-W(1)-C(29)	86.79(8)
C(22)-W(1)-C(29)	95.31(10)
N(3)-W(1)-C(1)	87.05(8)
O(1)-W(1)-C(1)	82.20(8)
C(22)-W(1)-C(1)	108.30(9)
C(29)-W(1)-C(1)	155.70(9)
N(1)-C(1)-N(2)	103.7(2)
N(1)-C(1)-W(1)	129.98(17)
N(2)-C(1)-W(1)	126.28(17)
C(36)-O(1)-W(1)	163.05(16)
C(1)-N(1)-C(2)	111.0(2)
C(1)-N(1)-C(4)	125.2(2)
C(2)-N(1)-C(4)	123.7(2)
C(1)-N(2)-C(3)	110.8(2)

C(1)-N(2)-C(7)	125.9(2)
C(3)-N(2)-C(7)	123.2(2)
C(3)-C(2)-N(1)	107.3(2)
C(3)-C(2)-H(2)	126.4
N(1)-C(2)-H(2)	126.4
C(10)-N(3)-W(1)	167.60(17)
C(2)-C(3)-N(2)	107.1(2)
C(2)-C(3)-H(3)	126.4
N(2)-C(3)-H(3)	126.4
N(1)-C(4)-C(6)	110.0(2)
N(1)-C(4)-C(5)	111.0(2)
C(6)-C(4)-C(5)	111.5(2)
N(1)-C(4)-H(4)	108.1
C(6)-C(4)-H(4)	108.1
C(5)-C(4)-H(4)	108.1
C(4)-C(5)-H(5A)	109.5
C(4)-C(5)-H(5B)	109.5
H(5A)-C(5)-H(5B)	109.5
C(4)-C(5)-H(5C)	109.5
H(5A)-C(5)-H(5C)	109.5
H(5B)-C(5)-H(5C)	109.5
C(4)-C(6)-H(6A)	109.5
C(4)-C(6)-H(6B)	109.5
H(6A)-C(6)-H(6B)	109.5
C(4)-C(6)-H(6C)	109.5
H(6A)-C(6)-H(6C)	109.5
H(6B)-C(6)-H(6C)	109.5
N(2)-C(7)-C(8)	110.8(2)
N(2)-C(7)-C(9)	110.5(2)
C(8)-C(7)-C(9)	111.4(2)
N(2)-C(7)-H(7)	108
C(8)-C(7)-H(7)	108
C(9)-C(7)-H(7)	108
C(7)-C(8)-H(8A)	109.5
C(7)-C(8)-H(8B)	109.5
H(8A)-C(8)-H(8B)	109.5
C(7)-C(8)-H(8C)	109.5
H(8A)-C(8)-H(8C)	109.5
H(8B)-C(8)-H(8C)	109.5
C(7)-C(9)-H(9A)	109.5
C(7)-C(9)-H(9B)	109.5
H(9A)-C(9)-H(9B)	109.5
C(7)-C(9)-H(9C)	109.5
H(9A)-C(9)-H(9C)	109.5
H(9B)-C(9)-H(9C)	109.5
N(3)-C(10)-C(11)	119.5(2)

N(3)-C(10)-C(15)	120.0(2)
C(11)-C(10)-C(15)	120.5(2)
C(12)-C(11)-C(10)	117.8(3)
C(12)-C(11)-C(16)	119.3(2)
C(10)-C(11)-C(16)	122.9(2)
C(13)-C(12)-C(11)	122.2(3)
C(13)-C(12)-H(12)	118.9
C(11)-C(12)-H(12)	118.9
C(12)-C(13)-C(14)	119.4(3)
C(12)-C(13)-H(13)	120.3
C(14)-C(13)-H(13)	120.3
C(13)-C(14)-C(15)	121.5(3)
C(13)-C(14)-H(14)	119.2
C(15)-C(14)-H(14)	119.2
C(14)-C(15)-C(10)	118.5(3)
C(14)-C(15)-C(19)	120.6(2)
C(10)-C(15)-C(19)	120.9(2)
C(11)-C(16)-C(17)	112.6(2)
C(11)-C(16)-C(18)	111.4(2)
C(17)-C(16)-C(18)	109.7(2)
C(11)-C(16)-H(16)	107.6
C(17)-C(16)-H(16)	107.6
C(18)-C(16)-H(16)	107.6
C(16)-C(17)-H(17A)	109.5
C(16)-C(17)-H(17B)	109.5
H(17A)-C(17)-H(17B)	109.5
C(16)-C(17)-H(17C)	109.5
H(17A)-C(17)-H(17C)	109.5
H(17B)-C(17)-H(17C)	109.5
C(16)-C(18)-H(18A)	109.5
C(16)-C(18)-H(18B)	109.5
H(18A)-C(18)-H(18B)	109.5
C(16)-C(18)-H(18C)	109.5
H(18A)-C(18)-H(18C)	109.5
H(18B)-C(18)-H(18C)	109.5
C(15)-C(19)-C(20)	113.5(2)
C(15)-C(19)-C(21)	110.5(2)
C(20)-C(19)-C(21)	109.8(2)
C(15)-C(19)-H(19)	107.6
C(20)-C(19)-H(19)	107.6
C(21)-C(19)-H(19)	107.6
C(19)-C(20)-H(20A)	109.5
C(19)-C(20)-H(20B)	109.5
H(20A)-C(20)-H(20B)	109.5
C(19)-C(20)-H(20C)	109.5
H(20A)-C(20)-H(20C)	109.5

H(20B)-C(20)-H(20C)	109.5
C(19)-C(21)-H(21A)	109.5
C(19)-C(21)-H(21B)	109.5
H(21A)-C(21)-H(21B)	109.5
C(19)-C(21)-H(21C)	109.5
H(21A)-C(21)-H(21C)	109.5
H(21B)-C(21)-H(21C)	109.5
C(23)-C(22)-W(1)	138.81(19)
C(23)-C(22)-H(22)	110.6
W(1)-C(22)-H(22)	110.6
C(28)-C(23)-C(24)	117.2(2)
C(28)-C(23)-C(22)	122.5(2)
C(24)-C(23)-C(22)	120.1(2)
C(25)-C(24)-C(23)	121.3(3)
C(25)-C(24)-H(24)	119.3
C(23)-C(24)-H(24)	119.3
C(26)-C(25)-C(24)	120.4(3)
C(26)-C(25)-H(25)	119.8
C(24)-C(25)-H(25)	119.8
C(25)-C(26)-C(27)	119.2(3)
C(25)-C(26)-H(26)	120.4
C(27)-C(26)-H(26)	120.4
C(28)-C(27)-C(26)	120.9(3)
C(28)-C(27)-H(27)	119.6
C(26)-C(27)-H(27)	119.6
C(27)-C(28)-C(23)	121.0(3)
C(27)-C(28)-H(28)	119.5
C(23)-C(28)-H(28)	119.5
C(30)-C(29)-W(1)	119.01(16)
C(30)-C(29)-H(29A)	107.6
W(1)-C(29)-H(29A)	107.6
C(30)-C(29)-H(29B)	107.6
W(1)-C(29)-H(29B)	107.6
H(29A)-C(29)-H(29B)	107
C(31)-C(30)-C(35)	116.8(2)
C(31)-C(30)-C(29)	121.6(2)
C(35)-C(30)-C(29)	121.5(2)
C(32)-C(31)-C(30)	122.3(3)
C(32)-C(31)-H(31)	118.8
C(30)-C(31)-H(31)	118.8
C(33)-C(32)-C(31)	119.9(3)
C(33)-C(32)-H(32)	120.1
C(31)-C(32)-H(32)	120.1
C(32)-C(33)-C(34)	119.8(3)
C(32)-C(33)-H(33)	120.1
C(34)-C(33)-H(33)	120.1

C(35)-C(34)-C(33)	120.0(3)
C(35)-C(34)-H(34)	120
C(33)-C(34)-H(34)	120
C(34)-C(35)-C(30)	121.2(3)
C(34)-C(35)-H(35)	119.4
C(30)-C(35)-H(35)	119.4
O(1)-C(36)-C(39)	106.6(2)
O(1)-C(36)-C(37)	110.4(2)
C(39)-C(36)-C(37)	110.1(2)
O(1)-C(36)-C(38)	108.5(2)
C(39)-C(36)-C(38)	110.7(3)
C(37)-C(36)-C(38)	110.5(2)
C(36)-C(37)-H(37A)	109.5
C(36)-C(37)-H(37B)	109.5
H(37A)-C(37)-H(37B)	109.5
C(36)-C(37)-H(37C)	109.5
H(37A)-C(37)-H(37C)	109.5
H(37B)-C(37)-H(37C)	109.5
C(36)-C(38)-H(38A)	109.5
C(36)-C(38)-H(38B)	109.5
H(38A)-C(38)-H(38B)	109.5
C(36)-C(38)-H(38C)	109.5
H(38A)-C(38)-H(38C)	109.5
H(38B)-C(38)-H(38C)	109.5
C(36)-C(39)-H(39A)	109.5
C(36)-C(39)-H(39B)	109.5
H(39A)-C(39)-H(39B)	109.5
C(36)-C(39)-H(39C)	109.5
H(39A)-C(39)-H(39C)	109.5
H(39B)-C(39)-H(39C)	109.5

Table S8. Anisotropic displacement parameters (Å^2 x 10^3) for **11**. The anisotropic displacement factor exponent takes the form: $-2\pi^2$ [$h^2 a^{*2} U_{11}$ + ... + 2 h k a* b* U_{12}].

	U11	U22	U33	U23	U13	U12
W(1)	15(1)	12(1)	13(1)	-1(1)	3(1)	-2(1)
C(1)	18(1)	20(1)	13(1)	-1(1)	3(1)	-2(1)
O(1)	22(1)	16(1)	17(1)	2(1)	-1(1)	0(1)
N(1)	24(1)	15(1)	20(1)	-3(1)	7(1)	-3(1)
N(2)	25(1)	14(1)	20(1)	-2(1)	8(1)	-3(1)
C(2)	34(2)	13(1)	33(2)	-8(1)	9(1)	-2(1)
N(3)	21(1)	16(1)	12(1)	1(1)	6(1)	-2(1)

C(3)	32(2)	12(1)	31(2)	-2(1)	8(1)	-6(1)
C(4)	25(1)	19(1)	28(2)	-4(1)	13(1)	-3(1)
C(5)	36(2)	26(2)	50(2)	-2(1)	24(2)	4(1)
C(6)	44(2)	47(2)	34(2)	4(2)	26(2)	2(2)
C(7)	25(1)	20(1)	28(2)	-2(1)	13(1)	-10(1)
C(8)	24(1)	35(2)	33(2)	1(1)	10(1)	-8(1)
C(9)	37(2)	38(2)	28(2)	1(1)	15(1)	-11(1)
C(10)	17(1)	17(1)	17(1)	4(1)	3(1)	-4(1)
C(11)	26(1)	22(1)	18(1)	2(1)	3(1)	-5(1)
C(12)	28(2)	44(2)	21(2)	-5(1)	0(1)	-8(1)
C(13)	20(1)	57(2)	35(2)	-1(2)	-3(1)	-4(2)
C(14)	19(1)	40(2)	31(2)	2(1)	4(1)	1(1)
C(15)	19(1)	23(1)	24(2)	5(1)	5(1)	-2(1)
C(16)	27(1)	29(2)	17(1)	-5(1)	3(1)	-4(1)
C(17)	46(2)	44(2)	19(2)	-5(1)	8(1)	-8(2)
C(18)	40(2)	32(2)	30(2)	-4(1)	5(1)	1(1)
C(19)	20(1)	28(2)	23(1)	2(1)	8(1)	4(1)
C(20)	43(2)	54(2)	34(2)	4(2)	22(2)	-4(2)
C(21)	61(2)	31(2)	40(2)	-1(2)	11(2)	-2(2)
C(22)	20(1)	15(1)	21(1)	-3(1)	8(1)	-2(1)
C(23)	31(1)	15(1)	18(1)	-6(1)	12(1)	-8(1)
C(24)	36(2)	22(2)	29(2)	-3(1)	17(1)	-8(1)
C(25)	48(2)	29(2)	34(2)	-3(1)	26(2)	-14(1)
C(26)	60(2)	25(2)	21(2)	0(1)	14(1)	-20(1)
C(27)	36(2)	29(2)	25(2)	5(1)	4(1)	-10(1)
C(28)	29(1)	22(1)	22(1)	1(1)	10(1)	-9(1)
C(29)	20(1)	19(1)	21(1)	-5(1)	5(1)	0(1)
C(30)	25(1)	10(1)	23(1)	-4(1)	3(1)	3(1)
C(31)	32(2)	16(1)	24(1)	-5(1)	6(1)	4(1)
C(32)	37(2)	27(2)	22(2)	-8(1)	-1(1)	9(1)
C(33)	24(2)	25(2)	41(2)	-15(1)	-6(1)	5(1)
C(34)	25(1)	19(1)	47(2)	-7(1)	7(1)	-2(1)
C(35)	26(1)	17(1)	26(2)	-3(1)	4(1)	2(1)
C(36)	29(1)	17(1)	18(1)	2(1)	-1(1)	3(1)
C(37)	48(2)	21(2)	23(2)	2(1)	8(1)	12(1)
C(38)	93(3)	26(2)	40(2)	-4(2)	39(2)	-3(2)
C(39)	28(2)	35(2)	58(2)	-4(2)	-12(2)	4(1)

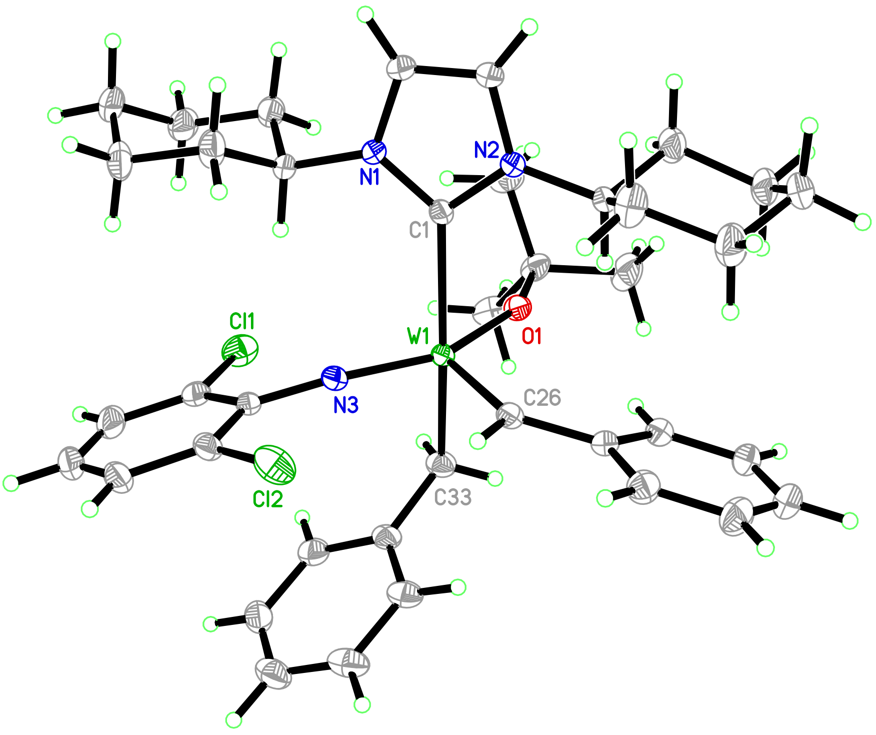

Figure S51. Single crystal X-ray structure of **5** displayed as a thermal ellipsoid plot (50% probability). Relevant bond lengths (pm) and angles (°): W(1)-N(3) 1.7749(18), W(1)-O(1) 1.9119(15), W(1)-C(26) 1.931(2), W(1)-C(33) 2.227(2), W(1)-C(1) 2.305(2), CL1-C(21) 1.733(3), CL2-C(17) 1.730(3), O(1)-C(22) 1.422(3), C(1)-N(1) 1.363(3), C(1)-N(2) 1.363(3), N(1)-C(2) 1.382(3), N(3)-W(1)-O(1) 150.42(8), N(3)-W(1)-C(26) 99.29(9), O(1)-W(1)-C(26) 110.24(8), N(3)-W(1)-C(33) 91.81(9), O(1)-W(1)-C(33) 84.10(8), C(26)-W(1)-C(33) 95.19(9), N(3)-W(1)-C(1) 93.70(8), O(1)-W(1)-C(1) 82.63(7), C(26)-W(1)-C(1) 100.88(8), C(33)-W(1)-C(1) 161.93(8), C(22)-O(1)-W(1) 154.88(15), N(1)-C(1)-N(2) 103.73(18), N(1)-C(1)-W(1) 130.05(15), N(2)-C(1)-W(1) 126.20(15), C(1)-N(1)-C(2) 111.35(18).

Table S9. Crystal data and structure refinement for **5**.

Empirical formula	$C_{41}H_{53}Cl_6N_3OW$
Formula weight	1000.41
Temperature	135(2) K
Wavelength	0.71073 Å
Crystal system, space group	Monoclinic, $P2_1/c$
Unit cell dimensions	a = 10.4540(3) Å, α = 90°<br>b = 21.3839(7) Å, β = 96.509(2)°<br>c = 19.0703(6) Å, γ = 90°
Volume	4235.6(2) $Å^3$
Z, Calculated density	4, 1.569 mg/m^3
Absorption coefficient	3.141 mm^{-1}
F(000)	2016
Crystal size	0.318 x 0.253 x 0.136 mm
Theta range for data collection	1.905 to 33.196 deg.
Limiting indices	-16<=h<=16, -32<=k<=32, -17<=l<=29
Reflections collected / unique	86552 / 16165 [R(int) = 0.0316]
Completeness to Θ = 25.242	100.00%
Absorption correction	Numerical
Max. and min. transmission	0.7551 and 0.5200
Refinement method	Full-matrix least-squares on F^2
Data / restraints / parameters	16165 / 6 / 484
Goodness-of-fit on F^2	1.042
Final R indices [I>2σ(I)]	R1 = 0.0296, wR2 = 0.0673

R indices (all data)	R1 = 0.0382, wR2 = 0.0698
Extinction coefficient	n/a
Largest diff. peak and hole	2.348 and -1.617 e. Å^{-3}

Table S10. Atomic coordinates (x 10^4) and equivalent isotropic displacement parameters (Å^2 x 10^3) for **5**. U(eq) is defined as one third of the trace of the orthogonalized U_{ij} tensor.

	x	y	z	U(eq)
W(1)	4288(1)	3734(1)	2106(1)	14(1)
CL1	7801(1)	3508(1)	2848(1)	30(1)
CL2	3576(1)	3454(1)	4230(1)	32(1)
O(1)	4444(2)	3834(1)	1123(1)	19(1)
C(1)	3819(2)	2717(1)	1770(1)	16(1)
N(1)	4482(2)	2179(1)	1941(1)	18(1)
N(2)	2786(2)	2523(1)	1323(1)	18(1)
C(2)	3876(2)	1668(1)	1608(1)	23(1)
C(3)	2822(2)	1885(1)	1217(1)	23(1)
N(3)	4960(2)	3521(1)	2970(1)	18(1)
C(4)	5720(2)	2120(1)	2389(1)	19(1)
C(5)	5580(3)	1693(1)	3021(1)	27(1)
C(6)	6873(3)	1608(2)	3472(1)	32(1)
C(7)	7898(3)	1364(1)	3034(2)	31(1)
C(8)	8038(2)	1800(1)	2414(2)	28(1)
C(9)	6756(2)	1880(1)	1955(1)	23(1)
C(10)	1732(2)	2918(1)	1002(1)	18(1)
C(11)	494(3)	2781(1)	1324(2)	30(1)
C(12)	-602(3)	3197(2)	997(2)	33(1)
C(13)	-781(3)	3157(1)	198(2)	33(1)
C(14)	474(3)	3295(2)	-102(1)	33(1)
C(15)	1535(3)	2848(1)	205(1)	29(1)
C(16)	5749(2)	3458(1)	3586(1)	19(1)
C(17)	5232(3)	3426(1)	4238(1)	23(1)
C(18)	5999(3)	3384(1)	4878(1)	30(1)
C(19)	7317(3)	3366(1)	4886(2)	35(1)
C(20)	7872(3)	3395(1)	4262(2)	32(1)
C(21)	7090(2)	3441(1)	3622(1)	23(1)
C(22)	5100(2)	3814(1)	510(1)	22(1)

C(23)	6346(3)	4187(1)	647(2)	32(1)
C(24)	4213(3)	4105(2)	-87(1)	36(1)
C(25)	5395(3)	3135(1)	349(1)	28(1)
C(26)	2621(2)	4021(1)	2316(1)	18(1)
C(27)	1622(2)	4395(1)	1906(1)	18(1)
C(28)	1790(2)	4665(1)	1252(1)	21(1)
C(29)	829(3)	5023(1)	887(1)	26(1)
C(30)	-323(3)	5122(1)	1164(2)	30(1)
C(31)	-509(3)	4864(1)	1814(2)	31(1)
C(32)	459(2)	4507(1)	2182(1)	25(1)
C(33)	5215(3)	4673(1)	2205(1)	22(1)
C(34)	5447(2)	4928(1)	2935(1)	20(1)
C(35)	4435(3)	5112(1)	3307(1)	25(1)
C(36)	4660(3)	5314(1)	4002(1)	31(1)
C(37)	5892(3)	5338(1)	4348(1)	32(1)
C(38)	6908(3)	5169(1)	3988(1)	31(1)
C(39)	6690(3)	4971(1)	3290(1)	26(1)
C(1X)	2119(3)	2284(2)	3252(2)	37(1)
CL1X	1671(1)	1525(1)	2988(1)	61(1)
CL2X	779(1)	2757(1)	3361(1)	36(1)
C(1Y)	1303(5)	5072(3)	4139(3)	81(2)
CL1Y	346(2)	5670(1)	3717(1)	118(1)
CL2Y	395(1)	4429(1)	4305(1)	71(1)

Table S11. Bond lengths [Å] and angles [°] for **5**.

W(1)-N(3)	1.7749(18)
W(1)-O(1)	1.9119(15)
W(1)-C(26)	1.931(2)
W(1)-C(33)	2.227(2)
W(1)-C(1)	2.305(2)
CL1-C(21)	1.733(3)
CL2-C(17)	1.730(3)
O(1)-C(22)	1.422(3)
C(1)-N(1)	1.363(3)
C(1)-N(2)	1.363(3)
N(1)-C(2)	1.382(3)
N(1)-C(4)	1.473(3)
N(2)-C(3)	1.380(3)
N(2)-C(10)	1.467(3)
C(2)-C(3)	1.341(3)
C(2)-H(2)	0.95
C(3)-H(3)	0.95
N(3)-C(16)	1.363(3)
C(4)-C(9)	1.525(3)

C(4)-C(5)	1.531(3)
C(4)-H(4)	1
C(5)-C(6)	1.529(4)
C(5)-H(5A)	0.99
C(5)-H(5B)	0.99
C(6)-C(7)	1.524(4)
C(6)-H(6A)	0.99
C(6)-H(6B)	0.99
C(7)-C(8)	1.525(4)
C(7)-H(7A)	0.99
C(7)-H(7B)	0.99
C(8)-C(9)	1.525(3)
C(8)-H(8A)	0.99
C(8)-H(8B)	0.99
C(9)-H(9A)	0.99
C(9)-H(9B)	0.99
C(10)-C(15)	1.519(3)
C(10)-C(11)	1.522(3)
C(10)-H(10)	1
C(11)-C(12)	1.528(4)
C(11)-H(11A)	0.99
C(11)-H(11B)	0.99
C(12)-C(13)	1.518(4)
C(12)-H(12A)	0.99
C(12)-H(12B)	0.99
C(13)-C(14)	1.518(4)
C(13)-H(13A)	0.99
C(13)-H(13B)	0.99
C(14)-C(15)	1.530(4)
C(14)-H(14A)	0.99
C(14)-H(14B)	0.99
C(15)-H(15A)	0.99
C(15)-H(15B)	0.99
C(16)-C(21)	1.397(3)
C(16)-C(17)	1.412(3)
C(17)-C(18)	1.385(3)
C(18)-C(19)	1.377(5)
C(18)-H(18)	0.95
C(19)-C(20)	1.383(5)
C(19)-H(19)	0.95
C(20)-C(21)	1.393(4)
C(20)-H(20)	0.95
C(22)-C(24)	1.518(4)
C(22)-C(25)	1.522(3)
C(22)-C(23)	1.524(4)
C(23)-H(23A)	0.98

C(23)-H(23B)	0.98
C(23)-H(23C)	0.98
C(24)-H(24A)	0.98
C(24)-H(24B)	0.98
C(24)-H(24C)	0.98
C(25)-H(25A)	0.98
C(25)-H(25B)	0.98
C(25)-H(25C)	0.98
C(26)-C(27)	1.468(3)
C(26)-H(26)	0.97(3)
C(27)-C(32)	1.399(3)
C(27)-C(28)	1.403(3)
C(28)-C(29)	1.386(3)
C(28)-H(28)	0.95
C(29)-C(30)	1.386(4)
C(29)-H(29)	0.95
C(30)-C(31)	1.390(4)
C(30)-H(30)	0.95
C(31)-C(32)	1.392(4)
C(31)-H(31)	0.95
C(32)-H(32)	0.95
C(33)-C(34)	1.489(3)
C(33)-H(33A)	0.95(3)
C(33)-H(33B)	0.90(3)
C(34)-C(35)	1.396(3)
C(34)-C(39)	1.399(3)
C(35)-C(36)	1.388(4)
C(35)-H(35)	0.95
C(36)-C(37)	1.381(4)
C(36)-H(36)	0.95
C(37)-C(38)	1.377(4)
C(37)-H(37)	0.95
C(38)-C(39)	1.391(4)
C(38)-H(38)	0.95
C(39)-H(39)	0.95
C(1X)-CL1X	1.746(3)
C(1X)-CL2X	1.760(3)
C(1X)-H(1X1)	0.99
C(1X)-H(1X2)	0.99
C(1Y)-CL2Y	1.720(5)
C(1Y)-CL1Y	1.761(7)
C(1Y)-H(1Y1)	0.99
C(1Y)-H(1Y2)	0.99
N(3)-W(1)-O(1)	150.42(8)
N(3)-W(1)-C(26)	99.29(9)

O(1)-W(1)-C(26)	110.24(8)
N(3)-W(1)-C(33)	91.81(9)
O(1)-W(1)-C(33)	84.10(8)
C(26)-W(1)-C(33)	95.19(9)
N(3)-W(1)-C(1)	93.70(8)
O(1)-W(1)-C(1)	82.63(7)
C(26)-W(1)-C(1)	100.88(8)
C(33)-W(1)-C(1)	161.93(8)
C(22)-O(1)-W(1)	154.88(15)
N(1)-C(1)-N(2)	103.73(18)
N(1)-C(1)-W(1)	130.05(15)
N(2)-C(1)-W(1)	126.20(15)
C(1)-N(1)-C(2)	111.35(18)
C(1)-N(1)-C(4)	126.94(18)
C(2)-N(1)-C(4)	121.64(18)
C(1)-N(2)-C(3)	111.13(18)
C(1)-N(2)-C(10)	126.12(18)
C(3)-N(2)-C(10)	122.72(19)
C(3)-C(2)-N(1)	106.6(2)
C(3)-C(2)-H(2)	126.7
N(1)-C(2)-H(2)	126.7
C(2)-C(3)-N(2)	107.1(2)
C(2)-C(3)-H(3)	126.4
N(2)-C(3)-H(3)	126.4
C(16)-N(3)-W(1)	164.08(18)
N(1)-C(4)-C(9)	110.24(18)
N(1)-C(4)-C(5)	110.59(19)
C(9)-C(4)-C(5)	111.29(19)
N(1)-C(4)-H(4)	108.2
C(9)-C(4)-H(4)	108.2
C(5)-C(4)-H(4)	108.2
C(6)-C(5)-C(4)	110.9(2)
C(6)-C(5)-H(5A)	109.5
C(4)-C(5)-H(5A)	109.5
C(6)-C(5)-H(5B)	109.5
C(4)-C(5)-H(5B)	109.5
H(5A)-C(5)-H(5B)	108
C(7)-C(6)-C(5)	111.3(2)
C(7)-C(6)-H(6A)	109.4
C(5)-C(6)-H(6A)	109.4
C(7)-C(6)-H(6B)	109.4
C(5)-C(6)-H(6B)	109.4
H(6A)-C(6)-H(6B)	108
C(6)-C(7)-C(8)	110.6(2)
C(6)-C(7)-H(7A)	109.5
C(8)-C(7)-H(7A)	109.5

C(6)-C(7)-H(7B)	109.5
C(8)-C(7)-H(7B)	109.5
H(7A)-C(7)-H(7B)	108.1
C(7)-C(8)-C(9)	111.0(2)
C(7)-C(8)-H(8A)	109.4
C(9)-C(8)-H(8A)	109.4
C(7)-C(8)-H(8B)	109.4
C(9)-C(8)-H(8B)	109.4
H(8A)-C(8)-H(8B)	108
C(4)-C(9)-C(8)	111.0(2)
C(4)-C(9)-H(9A)	109.4
C(8)-C(9)-H(9A)	109.4
C(4)-C(9)-H(9B)	109.4
C(8)-C(9)-H(9B)	109.4
H(9A)-C(9)-H(9B)	108
N(2)-C(10)-C(15)	111.68(19)
N(2)-C(10)-C(11)	110.73(19)
C(15)-C(10)-C(11)	111.1(2)
N(2)-C(10)-H(10)	107.7
C(15)-C(10)-H(10)	107.7
C(11)-C(10)-H(10)	107.7
C(10)-C(11)-C(12)	110.8(2)
C(10)-C(11)-H(11A)	109.5
C(12)-C(11)-H(11A)	109.5
C(10)-C(11)-H(11B)	109.5
C(12)-C(11)-H(11B)	109.5
H(11A)-C(11)-H(11B)	108.1
C(13)-C(12)-C(11)	112.2(2)
C(13)-C(12)-H(12A)	109.2
C(11)-C(12)-H(12A)	109.2
C(13)-C(12)-H(12B)	109.2
C(11)-C(12)-H(12B)	109.2
H(12A)-C(12)-H(12B)	107.9
C(12)-C(13)-C(14)	110.7(2)
C(12)-C(13)-H(13A)	109.5
C(14)-C(13)-H(13A)	109.5
C(12)-C(13)-H(13B)	109.5
C(14)-C(13)-H(13B)	109.5
H(13A)-C(13)-H(13B)	108.1
C(13)-C(14)-C(15)	110.8(2)
C(13)-C(14)-H(14A)	109.5
C(15)-C(14)-H(14A)	109.5
C(13)-C(14)-H(14B)	109.5
C(15)-C(14)-H(14B)	109.5
H(14A)-C(14)-H(14B)	108.1
C(10)-C(15)-C(14)	109.3(2)

C(10)-C(15)-H(15A)	109.8
C(14)-C(15)-H(15A)	109.8
C(10)-C(15)-H(15B)	109.8
C(14)-C(15)-H(15B)	109.8
H(15A)-C(15)-H(15B)	108.3
N(3)-C(16)-C(21)	123.5(2)
N(3)-C(16)-C(17)	120.6(2)
C(21)-C(16)-C(17)	115.9(2)
C(18)-C(17)-C(16)	122.5(3)
C(18)-C(17)-CL2	119.3(2)
C(16)-C(17)-CL2	118.21(18)
C(19)-C(18)-C(17)	119.4(3)
C(19)-C(18)-H(18)	120.3
C(17)-C(18)-H(18)	120.3
C(18)-C(19)-C(20)	120.4(2)
C(18)-C(19)-H(19)	119.8
C(20)-C(19)-H(19)	119.8
C(19)-C(20)-C(21)	119.7(3)
C(19)-C(20)-H(20)	120.2
C(21)-C(20)-H(20)	120.2
C(20)-C(21)-C(16)	122.1(2)
C(20)-C(21)-CL1	119.1(2)
C(16)-C(21)-CL1	118.71(18)
O(1)-C(22)-C(24)	107.1(2)
O(1)-C(22)-C(25)	108.87(18)
C(24)-C(22)-C(25)	111.1(2)
O(1)-C(22)-C(23)	109.0(2)
C(24)-C(22)-C(23)	110.6(2)
C(25)-C(22)-C(23)	110.2(2)
C(22)-C(23)-H(23A)	109.5
C(22)-C(23)-H(23B)	109.5
H(23A)-C(23)-H(23B)	109.5
C(22)-C(23)-H(23C)	109.5
H(23A)-C(23)-H(23C)	109.5
H(23B)-C(23)-H(23C)	109.5
C(22)-C(24)-H(24A)	109.5
C(22)-C(24)-H(24B)	109.5
H(24A)-C(24)-H(24B)	109.5
C(22)-C(24)-H(24C)	109.5
H(24A)-C(24)-H(24C)	109.5
H(24B)-C(24)-H(24C)	109.5
C(22)-C(25)-H(25A)	109.5
C(22)-C(25)-H(25B)	109.5
H(25A)-C(25)-H(25B)	109.5
C(22)-C(25)-H(25C)	109.5
H(25A)-C(25)-H(25C)	109.5

H(25B)-C(25)-H(25C)	109.5
C(27)-C(26)-W(1)	131.84(16)
C(27)-C(26)-H(26)	109.8(19)
W(1)-C(26)-H(26)	117.9(19)
C(32)-C(27)-C(28)	117.7(2)
C(32)-C(27)-C(26)	119.3(2)
C(28)-C(27)-C(26)	123.0(2)
C(29)-C(28)-C(27)	121.2(2)
C(29)-C(28)-H(28)	119.4
C(27)-C(28)-H(28)	119.4
C(28)-C(29)-C(30)	120.3(2)
C(28)-C(29)-H(29)	119.9
C(30)-C(29)-H(29)	119.9
C(29)-C(30)-C(31)	119.6(2)
C(29)-C(30)-H(30)	120.2
C(31)-C(30)-H(30)	120.2
C(30)-C(31)-C(32)	120.0(2)
C(30)-C(31)-H(31)	120
C(32)-C(31)-H(31)	120
C(31)-C(32)-C(27)	121.2(2)
C(31)-C(32)-H(32)	119.4
C(27)-C(32)-H(32)	119.4
C(34)-C(33)-W(1)	115.56(15)
C(34)-C(33)-H(33A)	109.6(19)
W(1)-C(33)-H(33A)	106.3(19)
C(34)-C(33)-H(33B)	112(2)
W(1)-C(33)-H(33B)	104(2)
H(33A)-C(33)-H(33B)	109(3)
C(35)-C(34)-C(39)	116.8(2)
C(35)-C(34)-C(33)	121.8(2)
C(39)-C(34)-C(33)	121.4(2)
C(36)-C(35)-C(34)	121.2(3)
C(36)-C(35)-H(35)	119.4
C(34)-C(35)-H(35)	119.4
C(37)-C(36)-C(35)	121.1(3)
C(37)-C(36)-H(36)	119.5
C(35)-C(36)-H(36)	119.5
C(38)-C(37)-C(36)	118.8(2)
C(38)-C(37)-H(37)	120.6
C(36)-C(37)-H(37)	120.6
C(37)-C(38)-C(39)	120.4(3)
C(37)-C(38)-H(38)	119.8
C(39)-C(38)-H(38)	119.8
C(38)-C(39)-C(34)	121.7(3)
C(38)-C(39)-H(39)	119.1
C(34)-C(39)-H(39)	119.1

CL1X-C(1X)-CL2X	112.15(17)
CL1X-C(1X)-H(1X1)	109.2
CL2X-C(1X)-H(1X1)	109.2
CL1X-C(1X)-H(1X2)	109.2
CL2X-C(1X)-H(1X2)	109.2
H(1X1)-C(1X)-H(1X2)	107.9
CL2Y-C(1Y)-CL1Y	111.6(3)
CL2Y-C(1Y)-H(1Y1)	109.3
CL1Y-C(1Y)-H(1Y1)	109.3
CL2Y-C(1Y)-H(1Y2)	109.3
CL1Y-C(1Y)-H(1Y2)	109.3
H(1Y1)-C(1Y)-H(1Y2)	108

Table S12. Anisotropic displacement parameters ($Å^2 \times 10^3$) for **5**. The anisotropic displacement factor exponent takes the form: $-2 \pi^2 [h^2 a^{*2} U_{11} + ... + 2 h k a^* b^* U_{12}]$.

W(1)	17(1)	13(1)	13(1)	0(1)	0(1)	0(1)
CL1	25(1)	27(1)	39(1)	-2(1)	6(1)	0(1)
CL2	33(1)	38(1)	24(1)	-4(1)	7(1)	-8(1)
O(1)	25(1)	17(1)	15(1)	1(1)	4(1)	2(1)
C(1)	18(1)	16(1)	13(1)	0(1)	-1(1)	0(1)
N(1)	21(1)	13(1)	18(1)	0(1)	-3(1)	1(1)
N(2)	21(1)	14(1)	17(1)	-1(1)	-3(1)	1(1)
C(2)	26(1)	14(1)	28(1)	-2(1)	-5(1)	1(1)
C(3)	24(1)	16(1)	26(1)	-4(1)	-6(1)	0(1)
N(3)	23(1)	15(1)	16(1)	-2(1)	-1(1)	0(1)
C(4)	20(1)	16(1)	19(1)	0(1)	-4(1)	3(1)
C(5)	27(1)	33(1)	20(1)	6(1)	1(1)	6(1)
C(6)	35(1)	38(2)	22(1)	6(1)	-6(1)	8(1)
C(7)	27(1)	28(1)	34(1)	3(1)	-9(1)	8(1)
C(8)	21(1)	28(1)	35(1)	0(1)	-1(1)	4(1)
C(9)	22(1)	24(1)	23(1)	2(1)	1(1)	3(1)
C(10)	20(1)	16(1)	17(1)	-2(1)	-4(1)	2(1)
C(11)	24(1)	38(2)	29(1)	8(1)	4(1)	5(1)
C(12)	22(1)	41(2)	34(1)	2(1)	-1(1)	6(1)
C(13)	28(1)	32(1)	36(1)	-6(1)	-14(1)	5(1)
C(14)	39(2)	37(2)	20(1)	2(1)	-8(1)	12(1)
C(15)	34(1)	35(1)	17(1)	-1(1)	-2(1)	11(1)
C(16)	26(1)	11(1)	18(1)	-2(1)	-5(1)	0(1)
C(17)	33(1)	16(1)	18(1)	-1(1)	-3(1)	-3(1)
C(18)	48(2)	21(1)	18(1)	0(1)	-7(1)	-1(1)
C(19)	47(2)	24(1)	28(1)	-2(1)	-18(1)	4(1)
C(20)	29(1)	24(1)	40(2)	-2(1)	-14(1)	3(1)
C(21)	27(1)	13(1)	27(1)	-2(1)	-3(1)	0(1)
C(22)	31(1)	19(1)	20(1)	5(1)	8(1)	4(1)

C(23)	37(1)	25(1)	37(1)	0(1)	18(1)	-3(1)
C(24)	49(2)	38(2)	22(1)	11(1)	6(1)	13(1)
C(25)	38(1)	21(1)	25(1)	-2(1)	9(1)	5(1)
C(26)	20(1)	17(1)	16(1)	-1(1)	1(1)	0(1)
C(27)	20(1)	15(1)	19(1)	-2(1)	0(1)	1(1)
C(28)	24(1)	20(1)	19(1)	-1(1)	0(1)	2(1)
C(29)	29(1)	22(1)	24(1)	2(1)	-2(1)	4(1)
C(30)	28(1)	27(1)	34(1)	-1(1)	-5(1)	8(1)
C(31)	22(1)	35(1)	35(1)	-1(1)	4(1)	8(1)
C(32)	23(1)	28(1)	25(1)	-1(1)	4(1)	2(1)
C(33)	27(1)	18(1)	20(1)	-1(1)	3(1)	-3(1)
C(34)	27(1)	13(1)	21(1)	-1(1)	2(1)	-4(1)
C(35)	30(1)	19(1)	27(1)	-5(1)	5(1)	-5(1)
C(36)	43(2)	23(1)	28(1)	-5(1)	12(1)	-6(1)
C(37)	54(2)	21(1)	21(1)	-1(1)	0(1)	-7(1)
C(38)	39(2)	21(1)	29(1)	1(1)	-10(1)	-1(1)
C(39)	28(1)	18(1)	30(1)	-3(1)	-1(1)	-1(1)
C(1X)	28(1)	42(2)	42(2)	4(1)	7(1)	-3(1)
CL1X	55(1)	52(1)	79(1)	-24(1)	19(1)	-6(1)
CL2X	29(1)	44(1)	34(1)	2(1)	3(1)	1(1)
C(1Y)	55(2)	123(4)	67(3)	-9(3)	17(2)	-39(3)
CL1Y	190(2)	96(1)	61(1)	26(1)	-19(1)	-77(1)
CL2Y	74(1)	63(1)	78(1)	-6(1)	17(1)	-5(1)

References

1. Gibson, V. C.; Kee, T. P.; Shaw, A., *Polyhedron* **1988,** *7*, 579-580.
2. Schrock, R. R.; DePue, R. T.; Feldman, J.; Yap, K. B.; Yang, D. C.; Davis, W. M.; Park, L.; DiMare, M.; Schofield, M., *Organometallics* **1990,** *9*, 2262-2275.
3. Tsang, W. C. P.; Hultzsch, K. C.; Alexander, J. B.; Bonitatebus, P. J.; Schrock, R. R.; Hoveyda, A. H., *J. Am. Chem. Soc.* **2003,** *125*, 2652-2666.
4. Kharasch, M. S.; Brown, H. C., *J. Am. Chem. Soc.* **1939,** *61*, 2142-2150.
5. Gal, J., *Acta Pharm Suec* **1977,** *14*, 335-344.
6. Solgadi, A.; Jean, L.; Lasne, M.-C.; Rouden, J.; Courtieu, J.; Meddour, A., *Tetrahedron: Asymm.* **2007,** *18*, 1511-1516.
7. Griffiths, D. C.; Young, G. B., *Organometallics* **1989,** *8*, 875-886.
8. Love, B. E.; Jones, E. G., *J. Org. Chem.* **1999,** *64*, 3755-3756.
